# Bioorthogonal, Bifunctional Linker for Engineering Synthetic Glycoproteins

**DOI:** 10.1021/jacsau.2c00312

**Published:** 2022-08-26

**Authors:** Ryan McBerney, Jonathan P. Dolan, Emma E. Cawood, Michael E. Webb, W. Bruce Turnbull

**Affiliations:** School of Chemistry and Astbury Centre for Structural Molecular Biology, University of Leeds, Leeds LS2 9JT, United Kingdom

**Keywords:** biorthogonal, conjugation, glycosylation, glycoprotein, bacterial toxin

## Abstract

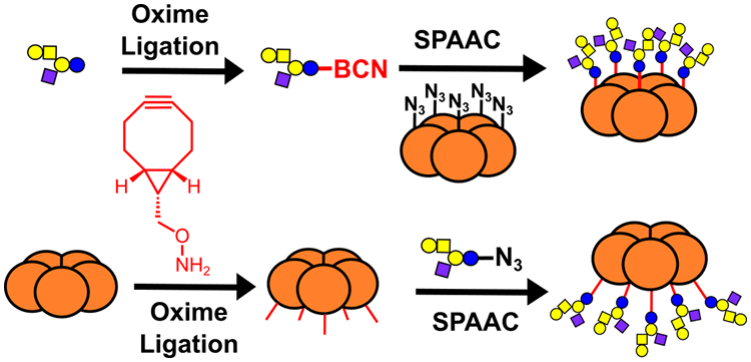

Post-translational glycosylation of proteins results in complex mixtures of heterogeneous protein glycoforms. Glycoproteins have many potential applications from fundamental studies of glycobiology to potential therapeutics, but generating homogeneous recombinant glycoproteins using chemical or chemoenzymatic reactions to mimic natural glycoproteins or creating homogeneous synthetic neoglycoproteins is a challenging synthetic task. In this work, we use a site-specific bioorthogonal approach to produce synthetic homogeneous glycoproteins. We develop a bifunctional, bioorthogonal linker that combines oxime ligation and strain-promoted azide–alkyne cycloaddition chemistry to functionalize reducing sugars and glycan derivatives for attachment to proteins. We demonstrate the utility of this minimal length linker by producing neoglycoprotein inhibitors of cholera toxin in which derivatives of the disaccharide lactose and **GM1os** pentasaccharide are attached to a nonbinding variant of the cholera toxin B-subunit that acts as a size- and valency-matched multivalent scaffold. The resulting neoglycoproteins decorated with GM1 ligands inhibit cholera toxin B-subunit adhesion with a picomolar IC_50_.

## Introduction

It is estimated that half of all proteins undergo glycosylation making it the most common post-translational modification.^1,2^ The complex, nontemplated biosynthetic pathways that introduce N- and O-linked glycans lead to high glycoprotein diversity, incorporating large numbers of highly complex oligosaccharides.^3−5^ While total chemical synthesis of glycoproteins has recently gained in popularity,^6−9^ methods for non-native glycan conjugation to make neoglycoproteins (a glycoprotein with a non-native linkage)^10^ remain important for glycoscience and glycotechnology.^11^ For many years, nucleophilic lysine side chains have been exploited through reductive amination^12^ for the production of glycoconjugate vaccines.^13−15^ However, this approach offers little control over the site or abundance of glycosylation due to the high prevalence of lysine on the surface of proteins.^16^ Cysteine residues have instead been used to gain more control, as their natural low surface abundance^17^ makes them useful reactive handles in site-specific protein engineering. Davis and co-workers, among others, have developed many ways in which cysteine can be exploited for the production of synthetic glycoproteins, including disulfide formation and chemical mutagenesis *via* a dehydroalanine intermediate.^18−20^ However, adding additional cysteine residues into a protein can introduce potential challenges, including disruption of protein folding, lowering expression levels.^21^

Advances in expanding the genetic code to introduce noncanonical amino acids into proteins^22−25^ have opened up new chemistries for protein modification, including copper-catalyzed azide–alkyne cycloaddition (CuAAC).^26,27^ However, despite the widespread application of the CuAAC reaction, including in the glycosylation of engineered proteins,^19,28−31^ the presence of copper catalyst used in CuAAC is not without its pitfalls.^32^ The use of cyclooctynes, rather than unstrained alkynes, in azide–alkyne reactions offers an alternative, copper-free biorthogonal route to covalent protein modification reactions.^33,34^ Such strain-promoted alkyne–azide cycloaddition (SPAAC) reactions^33,34^ have proved effective for the synthesis of neoglycoproteins through genetic incorporation of a bicyclononyne amino acid^35^ or through the use of cyclooctyne groups in complex multistep linker systems.^36^

Another important objective for neoglycoprotein preparation is the derivatization of unprotected glycans with suitable reactive groups for their attachment to the proteins.^37,38^ In this regard, oxime chemistry allows the latent aldehyde function of reducing sugars to be exploited to make a hydrolytically stable linker.^39,40^ The reaction can be catalyzed by anilines,^41−43^ and is also applicable to the modification of proteins containing aldehyde groups, for example, through N-terminal oxidation.^44−47^ We have previously used oxime ligation to a protein-derived aldehyde in the preparation of neoglycoproteins,^44^ in a case where CuAAC ligations with the protein proved problematic. However, this approach required extensive chemoenzymatic synthesis to generate complex derivatized glycans with a reactive handle.

**Figure 1:**
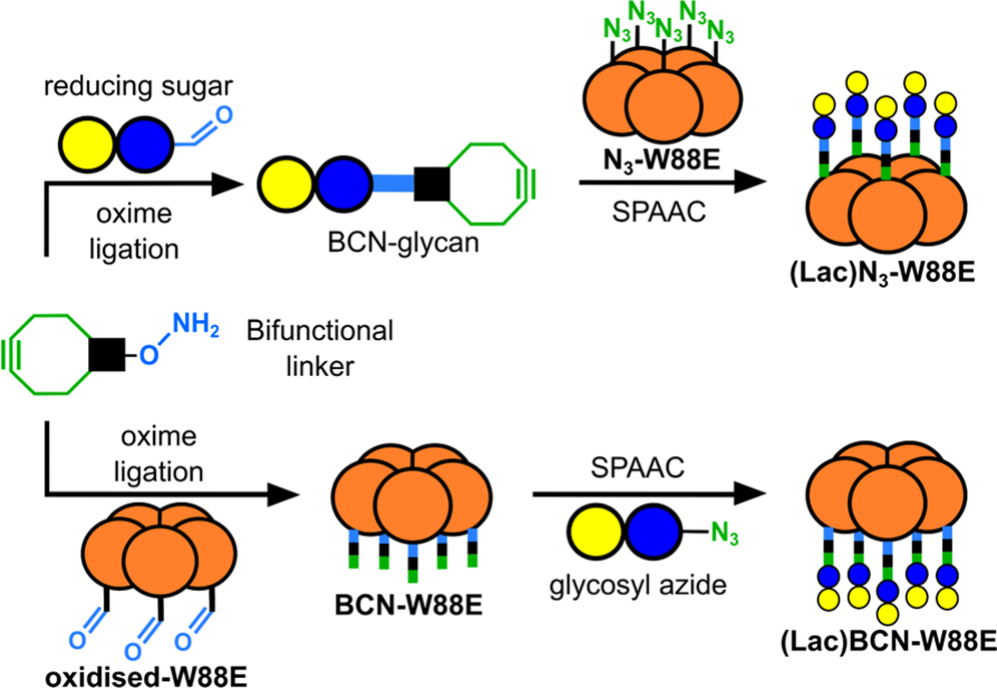
Cartoon representation of a bifunctional linker containing cyclooctyne and oxyamine functionalities that can be used to attach carbohydrates (yellow and blue circles) to proteins (orange) through oxime and SPAAC ligations.

In the work that we report here, our aim was to develop a simpler, flexible approach to neoglycoprotein synthesis that could be used for the functionalization of either chemically derivatized or reducing sugars. Our approach is to use a heterobifunctional linker, which combines an alkoxyamine for oxime formation with a strained alkyne for SPAAC conjugation (Figure 1). Thus, a single linker could be used either to connect a reducing sugar to an azide-functionalized protein or an azido-sugar to a protein with an N-terminal serine or threonine residue (following oxidation to generate an N-terminal glyoxyl residue).

Our strategy is exemplified through the synthesis of neoglycoprotein inhibitors of cholera toxin adhesion. Cholera toxin produced by *Vibrio cholerae* is the archetypal example of the AB_5_ bacterial toxin family that also includes *E. coli* heat-labile toxins and shiga-like toxins that cause severe diarrheal diseases.^48^ Cholera toxin has a single toxic A-subunit, which is an ADP-ribosyl transferase that is delivered into cells by a pentameric B-subunit (CTB) that is a sugar-binding protein that recognizes the glycolipid ganglioside GM1 (monosialotetrahexosylganglioside) and fucosylated structures.^49−51^ Inhibition of these protein–carbohydrate interactions can prevent the toxin from entering cells and thus prevent its toxic effects. Multivalent glycoconjugates have frequently been investigated as inhibitors of CTB adhesion.^52−55^ We have previously reported that neoglycoproteins based on a nonbinding mutant of the CTB (**W88E**, Figure 1) that have glycans matching the spacing and valency of the CTB binding sites are potent inhibitors of CT adhesion.^44^ Here, we investigate the effect of linker length and site of attachment to the protein scaffold on the activity of such inhibitors.

## Results and Discussion

### Synthesis of a New Bifunctional Linker

**Scheme 1:**
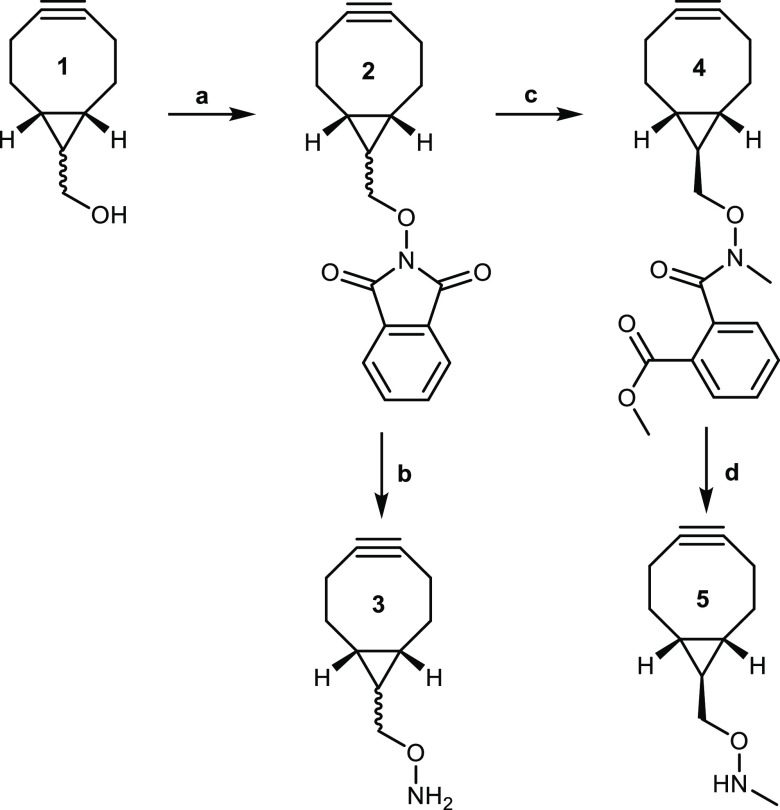
Synthesis of Novel Bifunctional Linkers **3** and **5** Starting with Either **1-*Exo*** Or **1-*Endo*** (a) *N*-hydroxyphthalimide, DIAD, PPh_3_, DCM, room temperature (rt), 5 h, **2-*exo*** 71%; **2-*endo*** 86%. (b) Methanolic methylamine, rt, 5 min; **3-*endo*** was isolated in 64%; **3-*exo*** was always used directly in subsequent reactions without isolation. (c) Only the *exo*-isomer of **2** was used for this reaction (i) NaOMe, MeOH, 30 min. (ii) MeI, MeOH, 1 h, quantitative. (d) *i*PrMgBr, toluene, rt, 2 h, 60% yield.

Among the many strained alkynes that have been extensively reviewed,^24,56,57^ bicyclo-[3.0.0]-nonyne (BCN) reported by Dommerholt et al.^58^ offered a desirable combination of symmetry and short linker length while maintaining a good balance between reaction kinetics and hydrophobicity.^34^ The known *exo-* and *endo*-isomers of bicyclononyne alcohol **1**(58) were each converted to phthalimide-protected oxyamines **2** under Mitsunobu conditions in 71–86% yield (Scheme 1). The free oxyamines **3** could be accessed following deprotection of **2** by MeNH_2_ in anhydrous methanol. Oxyamines **3** were found to be particularly susceptible to reaction with any traces of aldehydes or ketones present within the laboratory, and so all further reactions involving **3** were performed following *in situ* deprotection of **2** in laboratories in which acetone was not in use.

While condensation of sugars with primary *O*-alkyloxyamines typically leads to products with the open-chain oxime configuration, condensation with *N,O*-dialkyloxyamines will lead to glycosylamines that preserve the pyranose ring of the original sugar.^59^ Therefore, the *N*-methyl derivative **5** was synthesized from the *exo*-isomer of intermediate **2** using a different deprotection strategy. The phthalimide protecting group was first subjected to ring-opening with excess sodium methoxide followed by *in situ* methylation of the nitrogen using methyl iodide to yield the Weinreb amide **4**. Cleavage of Weinreb amide **4** using isopropyl Grignard reagent (step d in Scheme 1) yielded linker **5** in 60% yield. The development of two differentiating deprotection methods provides a facile route to both linkers **3** and **5** from the same intermediate.

### BCN Derivatization of Reducing Sugars

**Scheme 2:**
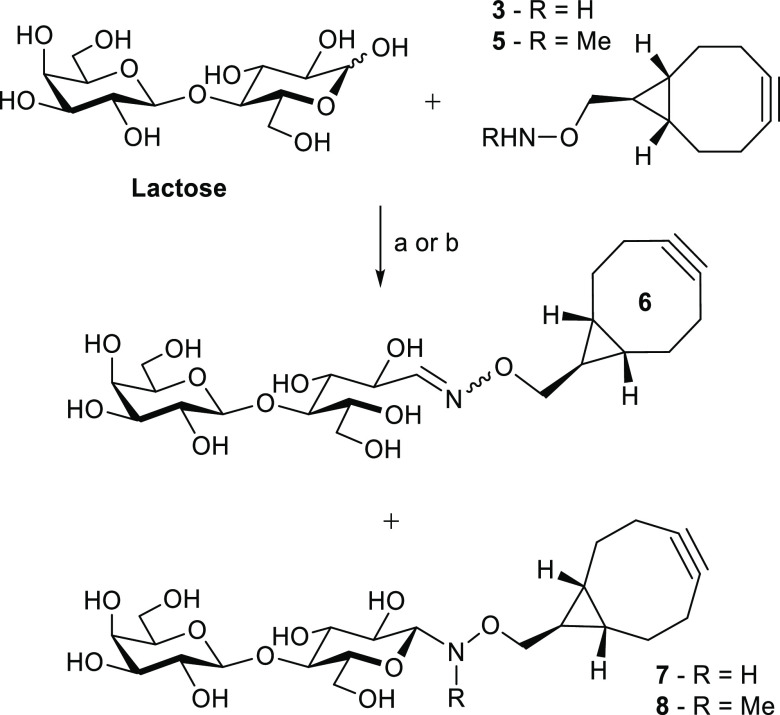
Oxime Ligation of Exo-Linker **3** or **5** to Unprotected Lactose Conditions: (a) CHCl_3_–MeOH (1:1), 50 °C, 24 h (69% **6** + **7**); (b) 1 M sodium acetate buffer, pH 5, 50 °C, 24–48 h (73% **6** + **7**; 40% **8**).

Using lactose as a model oligosaccharide (Scheme 2), oxime ligation was carried out with linker **3-*exo*** using either a methanol/chloroform solvent mixture^60^ or sodium acetate buffer (pH 5).^61^ In line with previous reports, reactant concentrations of at least 250 mM were required for effective conversion to oxime products.^62^ The oxime products were isolated in 69 and 73% yield, respectively. Conveniently, the hydrophobic properties of the BCN group allow it to be exploited as a “purification tag” for reverse-phase chromatography of the conjugation product, thus separating it from the excess lactose. NMR analysis of the glycoconjugate resulting from a reaction of lactose and linker **3-*exo*** showed it exists predominately in the ring-open form with a ratio of 20:5:14 for isomers **6**(E)/**6**(Z)/**7** (Lac-BCN). Reaction of methylaminooxy linker **5** with lactose was also achieved under the same conditions to produce exclusively the ring-closed isomer **8** in 40% yield.

### Site-Specific SPAAC Glycosylation of Azido-CTB

**Figure 2:**
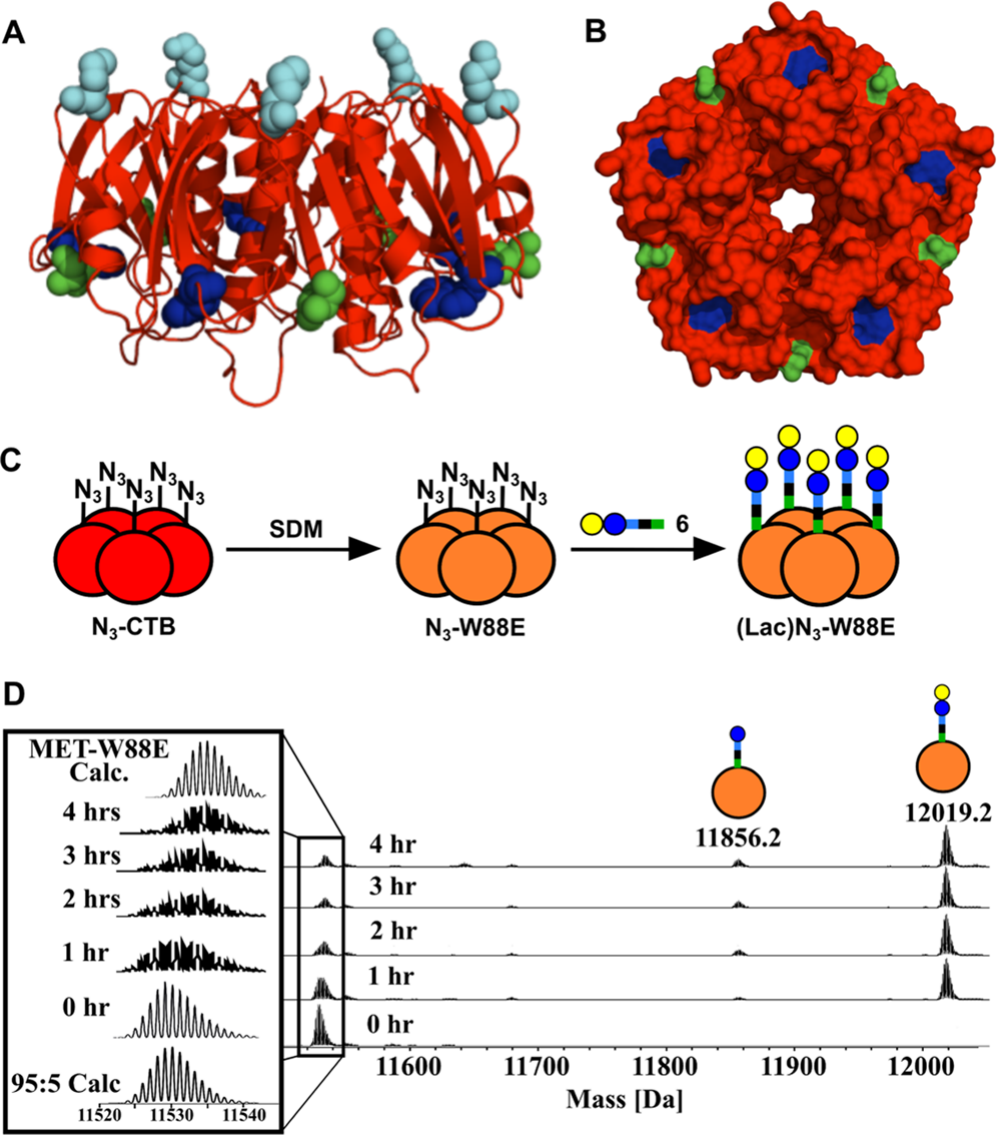
(A) Cartoon (side view) and (B) surface representation (bottom view) of the cholera toxin B-subunit (CTB) showing lysine 43 (cyan), which is the site for introduction of azidohomoalanine (Aha); also highlighted are tryptophan 88 (blue), which is mutated to glutamate to provide a nonbinding CTB variant **CTB-W88E**, and threonine 1 (green), which can be oxidized by periodate to introduce an aldehyde for oxime ligation. (C) Generation of **N**_**3**_**-W88E** by site-directed mutagenesis of azido-CTB, followed by SPAAC ligation of lactose derivative **6** and **N**_**3**_**-W88E**. (D) Electrospray ionization mass spectrometry (ESI-MS) time course of SPAAC ligation of **N**_**3**_**-W88E** and lactose derivative **6** over first 4 h shows complete conversion of the azido-CTB to leave a mass spectrometry signal matching that for the **Met-W88E** protein.

We next sought to use the strained alkyne derivative **6**, generated from lactose, to generate a neoglycoprotein. We used a nonbinding CTB variant containing azidohomoalanine (Aha) in place of lysine 43 (Figure 2A, cyan). We have previously reported an azido-CTB protein,^63^ in which Aha was introduced through isosteric replacement of methionine. In this protein, all native methionine residues had been mutated to leucine, and a single methionine residue introduced in place of lysine 43 (M37L-M68L-M101L-K43M). For this work, an additional mutation of tryptophan 88 (Figure 2A,B, blue) to glutamate was required to remove the protein’s native GM1 binding capability.^64^ Methionine-auxotrophic *E. coli* (B834) were transformed with a plasmid (pSAB2.4) harboring the gene for M37L-M68L-M101L-K43M-W88E CTB (**Met-W88E**). The protein was overexpressed in a defined growth medium containing Aha in place of methionine so that the expressed protein contained Aha at position 43 (M37L-M68L-M101L-K43Aha-W88E CTB; **N**_**3**_**-W88E**).^65,66^ Comparison of the deconvolved electrospray ionization (ESI) mass spectra with simulated isotope patterns for the Aha and Met variants of the protein confirmed successful incorporation of Aha over methionine in approximately 95% of the CTB monomers (**N**_**3**_**-W88E**) (Figure S5). Circular dichroism spectroscopy confirmed **N**_**3**_**-W88E** was identical to wild-type CTB, indicating that the protein was correctly folded (Figure S6).

Modification of **N**_**3**_**-W88E** with strained alkyne **6** by SPAAC (Figure 2C) under optimized conditions (500 μM **N**_**3**_**-W88E**, 10 equiv of **6**, 37 °C, phosphate-buffered saline (PBS) (pH 7.2)) led to the quantitative conversion of Aha residues to the triazole derivative within 4 h (**(Lac)N**_**3**_**-W88E**) (Figure 2D). Analysis by ESI-MS also revealed a peak corresponding to the mass of **Met-W88E**, consistent with the estimated 5% proportion of the unmodified protein substrate (Figure 2D). Analysis by sodium dodecyl sulfate poly(acrylamide) gel electrophoresis (SDS-PAGE) showed only a single band for the neoglycoprotein (Figure S15). Quantitative modification of the Aha residues could also be achieved at lower protein concentrations of **N**_**3**_**-W88E** (100 μM) and 1.5 equiv of **6**; however, reaction times of 10–24 h were required.

### Site-Specific N-Terminal Glycosylation of CTB

**Figure 3:**
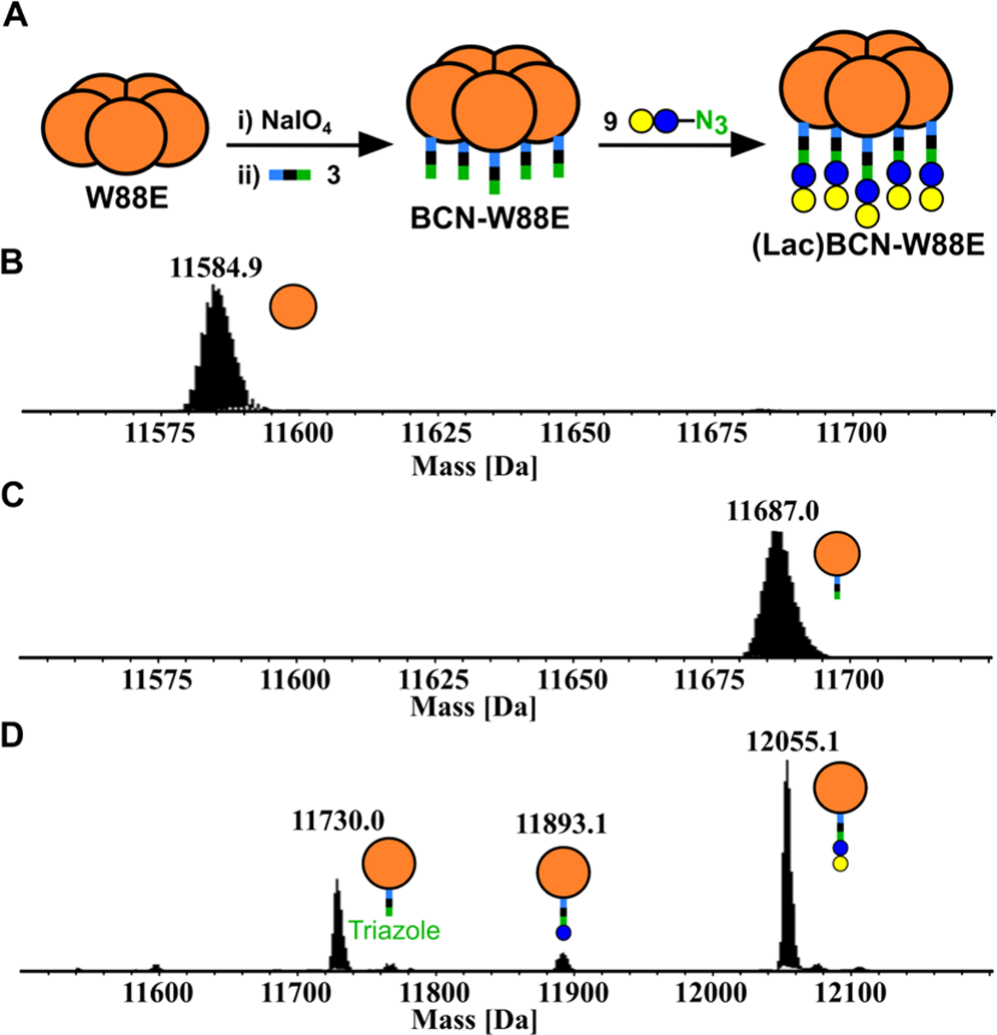
(A) Generation of **BCN-W88E** by N-terminal oxime ligation of endo-BCN-ONH_2_**3** to **W88E**, followed by SPAAC ligation of lactosyl azide **9** and **BCN-W88E**. (B) ESI-MS of **W88E** before treatment with NaIO_4_. (C) ESI-MS of **W88E** following oxidation with periodate, desalting and treatment of **oxidized-W88E** with ***endo-*3** and aniline, showing the quantitive formation of **BCN-W88E**. (D) ESI-MS of SPAAC ligation of **BCN-W88E** and lactosyl azide **9** after 8 h. Formation of **triazole-W88E** (11 730.0 Da) and **(Glc)BCN-W88E** (11 893.1 Da) as a result of fragmentation within the MS.

Having demonstrated that linker 3 can be used to attach a reducing sugar to an azide-functionalized protein, we then sought to apply the linker for attaching an azide-functionalized sugar to a protein aldehyde. For this study, we first functionalized CTB(W88E) with the *endo* isomer of oxyamine **3**, which could then be labeled using SPAAC ligation with a glycosyl azide (Figure 3A). The N-terminal threonine residue of **CTB-W88E** (Figure 2A, green) was oxidized using 5 equiv of sodium periodate in sodium phosphate buffer. This reaction typically reaches completion within 5 min in sodium phosphate, whereas the reaction does not reach completion in phosphate-buffered saline containing potassium ions, as these are known to hinder periodate reactivity.^67^ Oxime ligation of the **oxidized-W88E** (450 μM) with 10 equiv linker **3-*****endo*** in the presence of aniline (1% v/v) at 37 °C reached completion within 16 h (Figure 3C). When the **oxidized-W88E** was used at a concentration below 200 μM, a mixture of labeled product and N-terminal cyclization was observed.^68^ For optimal oxime ligation reactions, an **oxidized-W88E** concentration between 400 and 500 μM was required to obtain quantitative labeling while minimizing precipitation of the product **BCN-W88E**.

Lactosyl azide **9** was prepared from lactose through peracetylation and α-bromination at its reducing terminus,^69^ followed by S_N_2 displacement with sodium azide. After removal of the acetyl groups with sodium methoxide, lactosyl azide **9** was conjugated to **BCN-W88E** using a SPAAC reaction to give **(Lac)BCN-CTB** in quantitative yield in under 8 h. Analysis post-reaction by ESI-MS showed fragmentation peaks corresponding to the loss of terminal galactose and lactose (Figure 3D). The reaction of **BCN-W88E** in the presence of sodium azide (10 equiv) confirmed that no reaction occurs over the same time scale (Figure S12), demonstrating that the signal at 11 730 Da (Figure 3D) was the result of ion fragmentation rather than degradation of the lactosyl azide prior to reaction with the protein. Furthermore, the observation of a single band for the product in SDS-PAGE (Figure S16) was also consistent with the complete reaction of the protein to give **(Lac)BCN-W88E**.

### SPAAC Glycosylation for Synthesis of Neoglycoprotein Inhibitors of Cholera Toxin Adhesion

**Figure 4:**
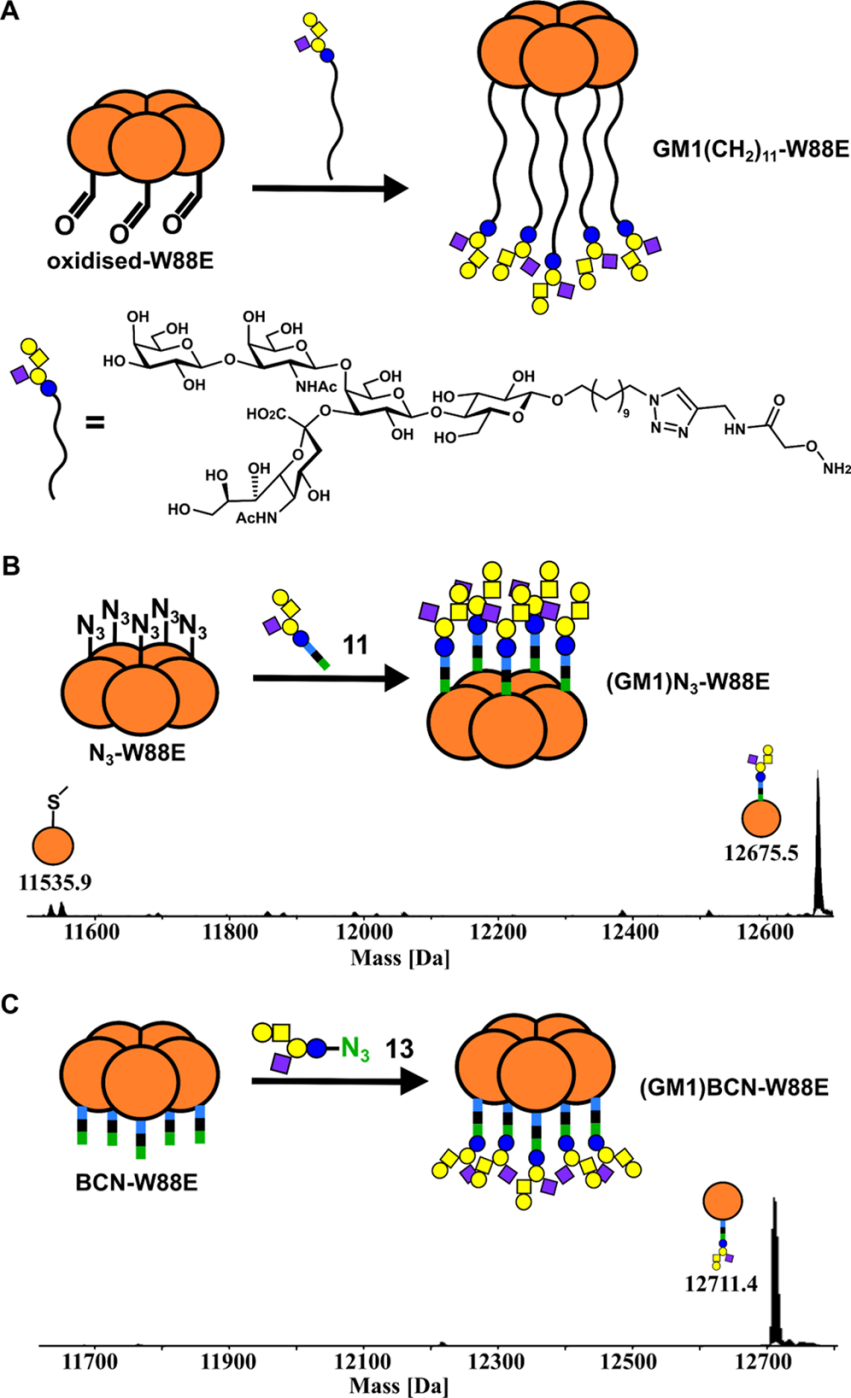
(A) Synthetic approach to a protein-based pentavalent neoglycoprotein inhibitor by Branson et al. involved oxime ligation of a GM1 derivative to the N-terminus of a nonbinding CTB variant.^44^ (B) SPAAC ligation of a BCN-GM1 **11** to the nonbinding CTB variant with azidohomoalanine incorporated at position 43. (C) SPAAC ligation of β-GM1 azide **13** to the **BCN-W88E** where linker **3** was site-specifically ligated to the N-terminus of **W88E** using oxime ligation.

Having shown that the nonbinding CTB variant **W88E** could be functionalized with simple disaccharides using linker **3**, these glycosylation methods were applied to the synthesis of a neoglycoprotein inhibitor of the cholera toxin. Branson et al. used the **W88E** nonbinding variant of CTB as a protein scaffold to develop the first neoglycoprotein-based inhibitor of CTB that matched the geometry of each GM1 ligand with the pentagonal symmetry of the target protein (Figure 4A).^44^ The neoglycoprotein was determined to be a highly potent inhibitor of cholera toxin adhesion, with an IC_50_ of 104 pM. This evidence supports the idea that the optimal design for multivalent inhibitors is to match the distance and geometry of the target binding sites, a hypothesis that also has support from theoretical studies on enhancing potency.^70^ However, we were curious to investigate if changing the site of glycosylation on the scaffold and length of linker might affect the activity of such neoglycoproteins. Therefore, we sought to attach the GM1 oligosaccharide to different sites on the CTB-W88E variants using bioorthogonal linker **3** (Figure 4B,C).

**Scheme 3:**
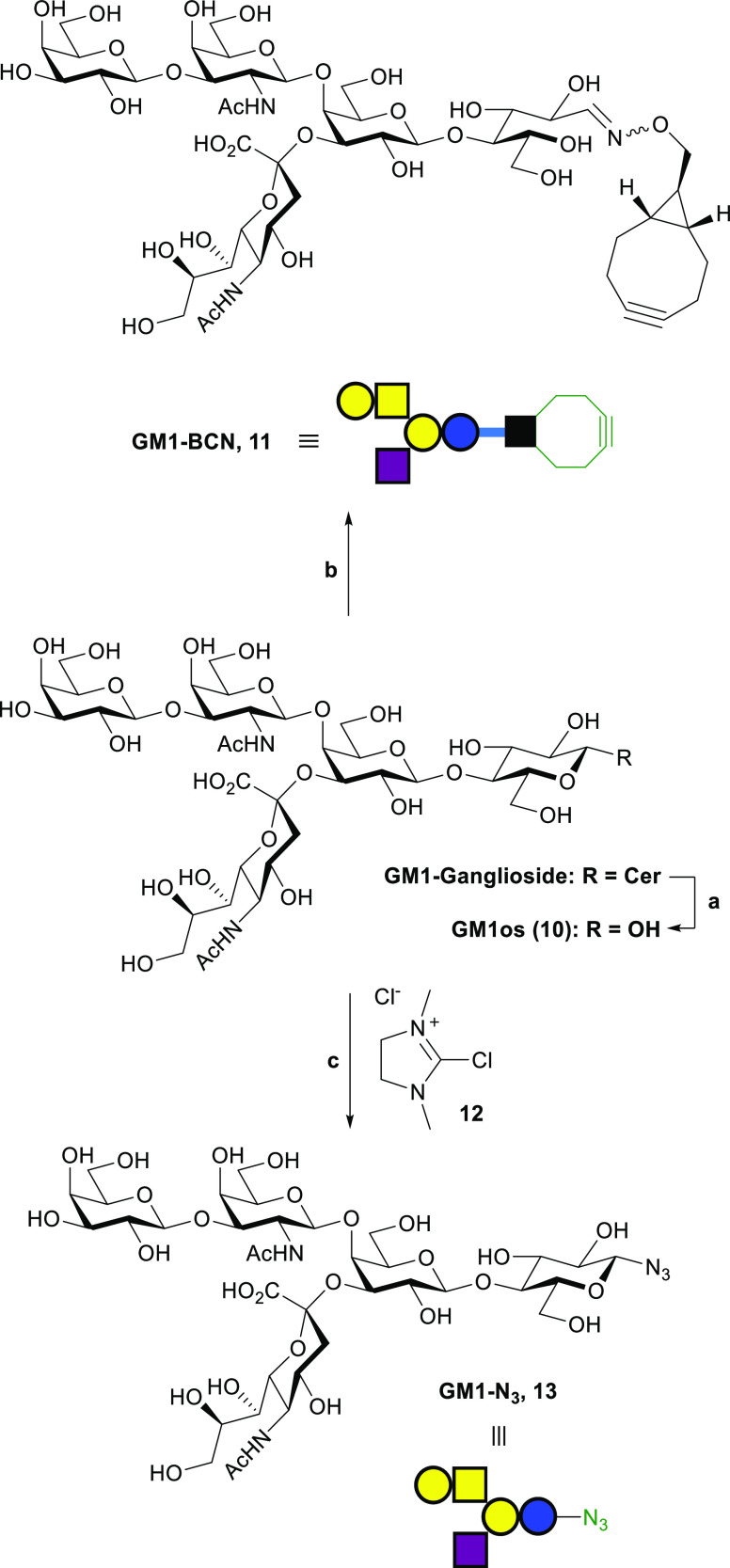
Enzymatic Hydrolysis of GM1 Ganglioside and Derivatization of GM1 Oligosaccharide **10** (A) EGCase II, 50 mM NaOAc buffer (pH 5), triton X-100 (0.2%), 37 °C, 5 days, 77% yield. (B) **3-*exo***, 1 M NaOAc buffer (pH 5), 50 °C, 48 h, 24% yield. (C) NaN_3_, TEA, H_2_O, 37 °C, 48 h, *ca.* 90% conversion to glycosyl azide.

GM1os pentasaccharide **10** was produced by enzymatic hydrolysis of GM1 ganglioside in a 77% yield using *Rhodococcus* sp. endoglycoceramidase II (EGCase II, Scheme 3).^71^ Enzymatic hydrolysis of GM1 ganglioside avoided the lengthy chemoenzymatic synthesis used previously.^44,72^ GM1os **10** was then derivatized with linker **3-*****exo*** by oxime ligation at 50 °C for 48 h, to make **GM1-BCN 11** (Scheme 3). The ^1^H NMR spectra for **GM1-BCN 11** indicated the presence of several isomers corresponding to the *E*- and *Z*-oximes and β-*N*-glycoside. Additional oxime signals increased in size over a period of 2 months, which we tentatively assign as the manno-configured C-2 epimer of **GM1-BCN 11** (see Supporting Information Figure S0).

Once again, the BCN group acted as a purification tag, allowing simple purification by reverse-phase chromatography and easy recovery of the unreacted starting oligosaccharide. While the presence of the ring-open form of reducing glucose unit differs from the structure of native GM1, it is unlikely to have any impact on inhibition as the glucose residue is not engaged in any interactions in the GM1 binding site.^73,74^ Furthermore, multivalent glycoconjugates of GM1 prepared by reductive amination, and thus fixed in the ring-open form, are known to be effective inhibitors.^75−77^ Nevertheless, even if the ring-closed form were advantageous for binding, the oxime derivatives are in equilibrium with the ring-closed glycosyloxyamines and so the system could adopt that configuration if preferable.

BCN-derivatized GM1 **11** (10 equiv) was attached to **N**_**3**_**-W88E** (500 μM), and ESI-HRMS confirmed quantitative labeling of the Aha residues in 5 h to give neoglycoprotein **(GM1)N**_**3**_**-W88E** (Figure 4B). SDS-PAGE analysis was similar to that for **(Lac)N**_**3**_**-W88E** (Figure S15) confirming that this ligation method is unaffected by increasing glycan complexity from a simple disaccharide to a branched pentasaccharide.

Complex glycosyl azides can be prepared from their reducing sugars using 2-chloro-1,3-dimethylimidazolinium chloride (DMC, **12**).^35,78^ DMC has previously been reported for derivatization of sialylated N-linked glycans, but not apparently for ganglioside-derived glycans. One-step conversion of **GM1os** pentasaccharide **10** to β-GM1 azide **13** was performed using DMC **12** (10 equiv) and sodium azide (44 equiv) in the presence of TEA (18 equiv) at 37 °C (Scheme 3). NMR and thin-layer chromatography (TLC) indicated that the reaction stopped after 90% conversion. Extending reaction time to over 48 h and adding further equivalents of sodium azide, DMC, or TEA had no further effect on the progress of the reaction. Therefore, the crude β-GM1 azide **13** was used for conjugation to **BCN-W88E**, resulting in complete conversion of the protein to **(GM1)BCN-W88E** using the optimized SPAAC conditions previously used for lactosyl azide **9** (Figure 4C).

### Inhibitor Potency against CTB Binding to GM1

**Figure 5:**
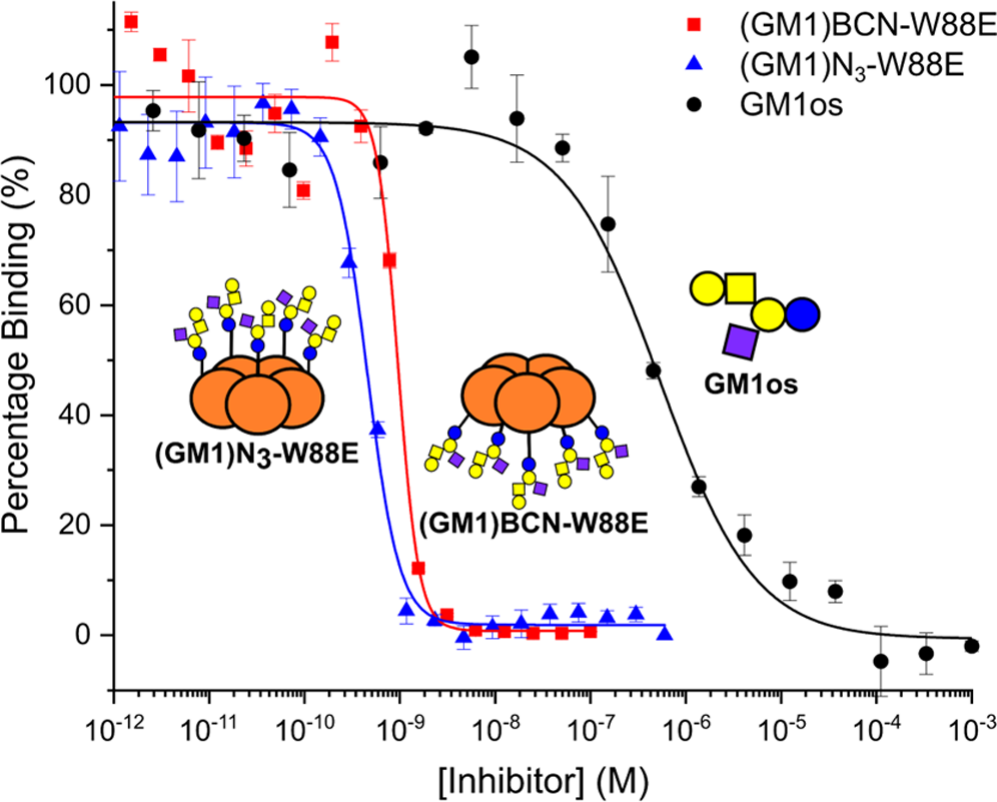
Inhibition of CTB–HRP conjugate binding to GM1-coated microtiter plates by **GM1os** (data reproduced from ref (44) under the terms of a Creative Commons Attribution License) and neoglycoproteins **(GM1)N**_**3**_**-W88E** and **(GM1)BCN-W88E**, determined by enzyme-linked lectin assay (ELLA). Error bars indicate the standard error of three measurements.

An enzyme-linked lectin assay (ELLA) was used to determine the IC_50_ of neoglycoproteins **(GM1)N**_**3**_**-W88E** and **(GM1)BCN-W88E** for the inhibition of wild-type CTB adhering to ganglioside GM1-coated microtiter plates. The **(GM1)N**_**3**_**-W88E** and **(GM1)BCN-W88E** neoglycoproteins were determined to have an IC_50_ of 457 and 924 pM, respectively (Figure 5 and Table 1). The analogous pentavalent lactose neoglycoprotein, **(Lac)N**_**3**_**-W88E**, produced by SPAAC of compound **6** and **N**_**3**_**-W88E**, demonstrated no inhibition of wild-type CTB binding to GM1 at concentrations up to 50 μM (data not shown).

**Table 1:** Inhibitory Potential of the **(GM1)N**_**3**_**-W88E** and **(GM1)BCN-W88E** Neoglycoproteins in Comparison to **GM1os**, as Determined by ELLA

inhibitor	valency	log(IC_50_)	IC_50_ (nM)^a^	relative potency (per GM1)^b^
**GM1os**^c^	1	–6.27 ± 0.04	530	1 (1)
**GM1(CH**_**2**_**)**_**11**_**-W88E**^c^	5	–9.98 ± 0.08	0.104	5096 (1019)
**(GM1)N**_**3**_**-W88E**	5	–9.34 ± 0.02	0.457	1160 (231)
**(GM1)BCN-W88E**	5	–9.03 ± 0.07	0.924	574 (115)

a As curve fitting was performed using log(IC_50_) as *x* values, calculated uncertainties in IC_50_ are asymmetric about the mean and have thus been omitted.

b Relative potency values are quoted compared to monovalent **GM1os**.

c Data reported by Branson et al.^44^ for monovalent **GM1os** and the previous neoglycoprotein inhibitor **GM1(CH**_**2**_**)**_**11**_**-W88E**.

Both GM1os-based neoglycoprotein inhibitors demonstrate over 100-fold enhancement in potency compared to monovalent **GM1os** (based on equivalent **GM1os** concentrations). Both neoglycoproteins were comparable in potency to the **GM1(CH**_**2**_**)**_**11**_**-W88E** reported previously (Table 1).^44^ Maintaining the same site of attachment while decreasing the length of the linker between the pentavalent scaffold and glycan or changing the site of glycosylation to the opposite face of the protein scaffold led to IC_50_ values that were within an order of magnitude of the **GM1(CH**_**2**_**)**_**11**_**-W88E** neoglycoprotein-based inhibitor. The fact that sub-nM IC_50_ values can be achieved across a range of neoglycoprotein designs demonstrates the robustness of this strategy for the preparation of multivalent inhibitors; the difference in potency between the neoglycoproteins presented in this work and the previously reported neoglycoprotein is minimal compared to the overall improvement observed over monovalent **GM1os** (Table 1). Nonetheless, the strategy for modification of **GM1os** presented here was performed in a single step prior to protein conjugation, compared to the lengthy chemoenzymatic synthesis of the inhibitor utilizing **11**-azidoundecyl GM1.^44,72^

## Conclusions

Here, we have developed a dual biorthogonal glycosylation strategy for the site-specific engineering of synthetic glycoproteins. The combination of two bioorthogonal reactive functional groups, oxyamine and cyclooctyne, into a single linker has demonstrated this to be a powerful method for synthetic glycosylation of proteins. The use of a divergent deprotection strategy in the synthesis of the bifunctional linkers **3** and **5** provides the opportunity to access both ring-open and ring-closed glycoconjugates. Functionalization of reducing oligosaccharides with linker **3** also facilitates efficient reverse-phase purification of the products. While CuAAC has often been used for the preparation of neoglycoproteins,^28−31^ SPAAC has been rarely used for this application; with fast reaction kinetics and high yields achieved, it offers a very convenient method for the site-specific production of homogeneous glycoproteins. SPAAC ligation of the BCN-glycan derivatives to azido-CTB(W88E) or functionalization of CTB(W88E) with linker **3***via* oxime ligation followed by SPAAC reaction with glycosyl azides both provide efficient routes to the preparation of neoglycoproteins that are potent multivalent inhibitors of cholera toxin adhesion. Altering the glycan attachment sites on the protein scaffold leads to only small variations in activity, demonstrating the robustness of a neoglycoprotein-based inhibitor strategy for effective inhibition of lectin adhesion. The bifunctional linkers described here could also be applied to the preparation of other synthetic protein conjugates, *e.g.*, antibody–drug conjugates, or for glycosylation of other biomolecules, *e.g.*, lipids or nucleic acids.

## Methods

### Synthesis of *O*-((1*R,*8*S,*9*s*)*-*Bicyclo[6.1.0]non-4-yn-9-ylmethyl)hydroxylamine (**3-*endo***) and Ganglioside GM1 Conjugates

#### 2-((1*R*,8*S*,9*r*)-Bicyclo[6.1.0]non-4-yn-9-ylmethoxy) Isolindine-1-3-dione (**2-*endo***)

Compound **1-*****endo*** ((1*R*,8*S*,9*s*)-bicyclo[6.1.0]non-4-yn-9-ylmethanol (20 mg, 0.13 mmol)), triphenylphosphine (38 mg, 0.15 mmol), and *N*-hydroxyphthalimide (24 mg, 0.15 mmol) were dissolved in anhydrous CH_2_Cl_2_ (1.2 mL) under a N_2_ atmosphere and chilled to 0 °C. Diisopropylazodicarboxylate (29 μL, 0.15 mmol) was added dropwise, and the solution was stirred for 10 min at 0 °C, after which the reaction was allowed to warm at room temperature over 4 h. The reaction mixture was concentrated in vacuo to yield a yellow residue. Purification by flash column chromatography (1:9 EtOAc/hexane) yielded the title compound (**2-*****endo***) as a white crystalline solid (37 mg, 94%). *R*_f_ 0.58 (3:7 EtOAc/hexane); ^1^H NMR (500 MHz; CDCl_3_) δ 1.09–1.02 (m, 2H), 1.56 (app. pent, *J* = 8.5 Hz, 1H), 1.69–1.60 (m, 2H), 2.25–2.18 (m, 2H), 2.34–2.26 (m, 4H), 4.31 (d, *J* = 8.0 Hz, 2H), 7.75 (dd, *J* = 5.4, 3.1 Hz, 2H), 7.83 (dd, *J* = 5.4, 3.1, Hz, 2H); ^13^C NMR (125 MHz; CDCl_3_) δ 17.3, 20.8, 21.5, 29.3, 76.4, 99.0, 123.6, 129.1, 134.6, 163.8; high-resolution mass spectrometry (HRMS) [ES+] C_18_H_17_NO_3_Na requires 318.1101. Found [M + Na]^+^ = 318.1099.

#### *O*-((1*R*,8*S*,9*s*)-Bicyclo[6.1.0]non-4-yn-9-ylmethyl)hydroxylamine (**3-*endo***)

Compound **2-*****endo*** (20 mg, 68 μmol) was added to anhydrous 2 M methanolic methylamine (0.169 mL, 339 μmol) under nitrogen in an oven-dried flask. The reaction was monitored by TLC and was found to be complete within 2 min. The product was diluted in 1:9 EtOAc/hexane and purified by flash column chromatography (1:9 EtOAc/hexane) to yield a colorless oil (7.2 mg, 64%). *R*_f_ 0.25 (3:7 EtOAc/hexane); ^1^H NMR (500 MHz; CDCl_3_) δ 0.96–0.86 (m, 2H), 1.35–1.25 (m, 1H), 1.62–1.52 (m, 2H), 2.34–2.16 (m, 6H), 3.75 (d, *J* = 7.7 Hz, 2H), 5.41 (br. s, 2H); ^13^C NMR (100 MHz; CDCl_3_) δ 17.6, 20.0, 21.6, 29.4, 73.2, 99.1; HRMS [ES+] C_10_H_16_NO requires 166.1226. Found [M + H]^+^ 166.1225.

#### β-d-Galactopyranosyl-(1→3)-2-acetamido-2-deoxy-β-d-galactopyranosyl-(1→4)-[(5-acetamido-3,5-dideoxy-d-glycero-α-d-galacto-non-2-ulopyranosylonic acid)-(2→3)]-β-d-galactopyranosyl-(1→4)-d-glucopyranose (1*R*,8*S*,9*r*)-bicyclo[6.1.0]non-4-yn-9-ylmethyloxime (GM1-BCN Oxime, **11**)

##### Method 1

β-d-Galactopyranosyl-(1→3)-2-acetamido-2-deoxy-β-d-galactopyranosyl-(1→4)-[(5-acetamido-3,5-dideoxy-d-glycero-α-d-galacto-non-2-ulopyranosylonic acid)-(2→3)]-β-d-galactopyranosyl-(1→4)-d-glucopyranose (**GM1os**, 1 mg, 1 μmol) was suspended in 1:1 CHCl_3_/MeOH (2 μL) in a 100 μL PCR tube. Linker **3-*exo*** (3 μL of 390 mM stock solution in 1:1 CHCl_3_/MeOH) was added to this suspension, and the reaction was heated at 50 °C for 48 h using a PCR thermocycler with a heated lid (105 °C). The crude mixture was diluted to 100 μL with water, and the product was isolated by reverse-phase extraction using a C18 SPE cartridge: excess **GM1os** was removed by extensive washing with water before elution of the product in 20% aq methanol. The title compound **11**, comprising a mixture of open-chain and cyclized isomers, was obtained as a white solid by lyophilization (0.38 mg, 33%).

##### Method 2

**GM1os** (1 mg, 1 μmol) was dissolved in H_2_O (2 μL) in a 100 μL PCR tube, to which NaOAc buffer (1 μL of a 5 M stock solution, pH 5) and linker **3-*exo*** (2 μL of a 390 mM stock solution in 1:1 CHCl_3_/MeOH) were added, and the reaction was heated at 50 °C for 48 h using a PCR thermocycler with a heated lid (105 °C). Completion of the reaction was confirmed by TLC (2:2:1 BuOH/MeOH/H_2_O). The crude mixture was diluted to 100 μL with water, and the product was isolated by reverse-phase extraction using a C18 SPE cartridge: excess **GM1os** was removed by extensive washing with water before elution of the product in 20% aq methanol. The title compound **11**, comprising a mixture of open-chain and cyclized isomers, was obtained as a white solid by lyophilization (0.28 mg, 24%).

*R*_f_ 0.63 (2:2:1 BuOH/MeOH/H_2_O); ^1^H NMR (500 MHz; D_2_O) δ 0.71–0.84 (m), 1.36–1.46 (m), 1.93 (t, 11.8 Hz), 2.00–2.05 (m), 2.12–2.21 (m), 2.25–2.35 (m), 2.40–2.47 (m), 2.64–2.68 (m), 3.29–4.19 (m), 4.30–4.32 (m, H_1*N*-glycoside_), 4.48–4.59 (m, H_1″,1*⁗*,_ H_2*E*-oxime_), 4.74 (m), 4.93–4.99 (m, H_2*Z*-oxime_) 6.93 (d, *J* = 5.4 Hz, H_1*Z*-oxime_), 6.97 (d, *J* = 4.9 Hz, H_1ep*Z*-oxime_), 7.48 (d, *J* = 6.9 Hz, H_1ep*E*-oxime_), 7.59 (d, *J* = 5.9 Hz, H_1*E*-oxime_); HRMS C_47_H_75_N_3_O_29_ + H requires 1146.4564. Found *m*/*z* [M + H]^+^ = 1146.4554.

#### β-d-Galactopyranosyl-(1→3)-2-acetamido-2-deoxy-β-d-galactopyranosyl-(1→4)-[(5-acetamido-3,5-dideoxy-d-glycero-α-d-galacto-non-2-ulopyranosylonic acid)-(2→3)]-β-d-galactopyranosyl-(1→4)-β-d-glucopyranosyl azide (GM1 azide, **13**)

**GM1os** (9 mg, 9 μmol), 2-chloro-1,3-dimethylimidazolium (DMC, 15 mg, 90 μmol), and NaN_3_ (25 mg, 400 μmol) were dissolved in H_2_O (0.1 mL) in a 1.5 mL Eppendorf tube. Triethylamine (22.5 μL, 161 μmol) was added, mixed by vortex, and the solution was incubated at 37 °C for 48 h. The product was isolated by size exclusion chromatography (Biogel P2) eluting with 20 mM ammonium formate to yield the title compound **13** as a white foam after lyophilization (8.6 mg, *ca.* 90% conversion to azide product with *ca.* 10% hemiacetal remaining). *R*_f_ 0.58 (2:2:1 *n*-BuOH/MeOH/H_2_O); ^1^H NMR (500 MHz; D_2_O; 275 K) δ 1.94 (t, *J* = 12.3 Hz, 1H), 1.99 (s, 3H), 2.01 (s, 3H), 2.63 (dd, *J* = 12.3, 4.5 Hz, 1H), 3.34 (dd, *J* = 9.3, 8.2 Hz, 1H), 3.53–3.45 (m, 2H), 3.99–3.54 (m, 24H), 4.01 (dd, *J* = 10.8, 8.7 Hz, 1H), 4.19–4.11 (m, 3H), 4.52 (d, *J* = 7.8 Hz, 1H), 4.53 (d, *J* = 7.7 Hz, 1H), 4.75 (d, *J* = 8.8 Hz, 1H), 4.78 (d, *J* = 8.8 Hz, 1H); HRMS [ES+]: C_37_H_61_N_5_O_28_Na requires 1046.3401. Found [M + Na]^+^ = 1046.3417.

## Supporting Information Available

Full experimental procedures for the preparation of compounds **2**–**9**, **11**, and **13**; experimental procedures for the preparation of proteins and protein conjugates and experimental procedures for plate-based assay of candidate inhibitors; ^1^H and ^13^C NMR spectra for organic compounds; and mass spectrometry data for modified proteins (PDF)

## Notes

The raw data associated with this paper including NMR and mass spectra are openly available from the University of Leeds data repository. https://doi.org/10.5518/1195
