# Supplementary material for: Bioorthogonal, Bifunctional Linker for Engineering Synthetic Glycoproteins
Source: JACS Au. 2022 Aug 26;2(9):2038–47. doi: 10.1021/jacsau.2c00312 (PMC9516712; doi:10.1021/jacsau.2c00312)

Bioorthogonal, Bifunctional Linker for Engineering Synthetic Glycoproteins

Ryan McBerney, Jonathan P. Dolan, Emma E. Cawood, Michael E. Webb* and W. Bruce Turnbull*

School of Chemistry and Astbury Centre for Structural Molecular Biology, University of Leeds, Leeds, LS2 9JT, United Kingdom.

*Corresponding authors: m.e.webb@leeds.ac.uk; w.b.turnbull@leeds.ac.uk

Supporting Information

General Methods S3

Synthesis of Linker 3 & 5 .... S4

Enzymatic hydrolysis of GM1 ganglioside S7

Synthesis of BCN glycan derivatives 6, 7 & 11 .... S8

Synthesis of glycosyl azides 9 & 13 .... S12

Synthesis of azidohomoalanine S16

Protein Expression & Purification S16

General Procedures for Protein Modification S24

ELLA protocols S30

References S31

NMR & MS Spectra S33

The raw data associated with this paper including NMR and mass spectra are openly available from the University of Leeds data repository. https://doi.org/10.5518/1195

General Methods

Unless stated otherwise, all starting materials and reagents were purchased from commercial suppliers and used without further purification. All solvents used were dried prior to use, according to standard methods, unless otherwise stated. Reactions were performed under an N_2 atmosphere and within glassware which was oven dried. Completion of reactions was initially determined by TLC and visualized using shortwave ultraviolet light (254 nm) and/or charring with 5% H_2SO_4/MeOH or $KMNO_4$. TLC plates used were Merck Silica-Gel 60 F_{254} Aluminium backed. Silica chromatography columns prepared using Fisher 60Å 43–60 micron silica gel. Lyophilisation carried out using Virtis Benchtop K freeze dryer. Water was purified by ELGA PURLAB classic. InertSep® SLIM C18-C reverse SPE phase cartridges were purchased from BGB Analytik.

NMR spectroscopy was recorded using Bruker AV3HD-400 (400 MHz, BBO Probe), Bruker AV4 NEO (500 MHz, BBO, TXI & TBO Probe) and Bruker AV4 NEO (500 MHz, C/H cryoprobe) spectrometers. NMR data is reported in parts per million (ppm) referenced to residual solvent signal at room temperature. The following abbreviations are used in 1H NMR analysis: Ar = aromatic, s = singlet, d = doublet, t = triplet, q = quartet, m= multiplet, dd = double doublet, dt = doublet of triplets, td = triplet of doublets and ddd = double double doublet.

HRMS was performed using Bruker Daltonics MicroTOF mass spectrometer employing electrospray (ES+) ionisation. LC-MS analysis performed on Brucker AmaZon X series LC-MS spectrometer. HILIC-LC-MS was performed on the same spectrometer using a Kinetex 2.6 mm HILIC 100 Å, LC column 30 x 2.1 mm purchased from Phenomenex. HRMS of protein samples was performed using a Bruker Daltonics MicroTOF mass spectrometer. Protein samples were loaded at a concentration of 20-40 µM (made up with H_2O) into the instrument before being automatically diluted into 0.1% TFA/50% MeCN (v/v) in H_2O prior to analysis.

Gel filtration chromatography was performed using a Biogel P2 (16/60), Superdex S75 (26/60) or Superdex S200 (16/60) attached to a GE Pharmacia ÄKTA FPLC system or BioRad NGC FPLC system.

Synthesis of Linker 3

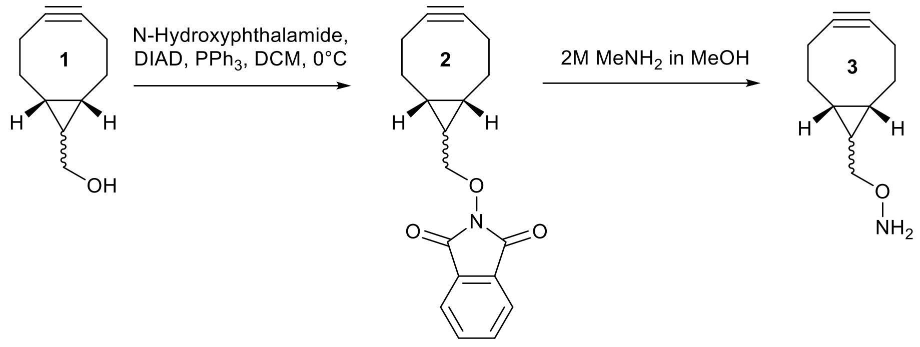

Compound 2: 2-((*1R,8S,9r*)-bicyclo[6.1.0]non-4-yn-9-ylmethoxy) isolindine-1-3-dione

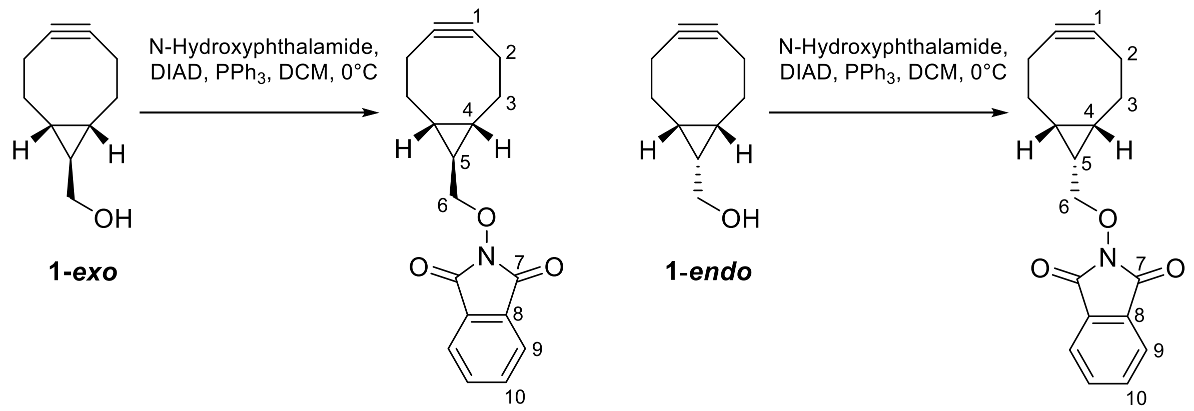

2-*exo* **2-*endo***

Compound **1-*exo*** (*1R,8S,9r*)-bicyclo[6.1.0]non-4-yn-9-ylmethanol[1] (240 mg, 1.6 mmol, 1 eq), triphenylphosphine (482 mg, 1.76 mmol, 1.1 eq) and *N*-hydroxyphthalamide (287 mg, 1.76 mmol, 1.1 eq) were dissolved in anhydrous DCM (16 mL, reaction concentration 0.1 M) and placed under a nitrogen atmosphere. The mixture was cooled to 0 °C, at which point DIAD (0.35 ml, 1.76 mmol, 1.1 eq) was added. The reaction was left stirring and allowed to warm to room temperature. After four hours the mixture was concentrated *in vacuo* to yield a crude residue, which was purified by flash column chromatography eluting in 10% EtOAc in hexane to 20% EtOAc in hexane to yield a white crystalline solid (281 mg, 60% yield)

Exo

R_f 0.42 (30% EtOAc in hexane); **^{1}H NMR (500 MHz; $CDCl_3$)** δ 0.78-0.88 (3H, m, **$H_{4,5}$**), 1.38-1.45 (2H, m, **H_3**), 2.14-2.20 (2H, m, **H_2**), 2.26-2.30 (2H, m, **$H_{2'}$**), 2.40 (2H, dd, *J* = 13.8 ,2.19 Hz, **$H_{3'}$**), 4.15 (2H, d, *J* = 7.0, **H_6**), 7.73-7.78 (2H, m, **H_{10}**) 7.82-7.87 (2H, m, **H_9**); **^{13}C NMR (125 MHz, $CDCl_3$)** δ 21.3 (**C_2**), 22.7 (**C_5**), 23.0 (**C_4**), 33.15 (**C_3**), 82.3 (**C_6**), 98.7 (**C_1**), 123.5 (**C_{10}**), 129.0 (**C_8**), 134.5 (**C_9**), 163.7 (**C_7**); **IR -** (v_{max}/cm^{-1}) **–** 1124 (C-O), 1720 (C=O); **HRMS –** $C_{18}H_{17}NO_3$+Na requires 318.1101; found $[M+Na]^+$ = 318.1101.

The same procedure starting from 20 mg **1-*endo*** yielded **2-*endo*** in a 94% yield.

Endo

R_f 0.58 (3:7 EtOAc/Hexane); **^{1}H NMR (500 MHz; $CDCl_3$)** δ 1.09–1.02 (2 H, m, **H_4**), 1.56 (pent J = 8.5 Hz 1H), 1.69–1.60 (2 H, m, **H_3**), 2.25–2.18 (2 H, m, **$H_{3'}$**), 2.34–2.26 (4 H, m, **H_2**), 4.31 (2 H, d, J = 8.0 Hz, **H_6**), 7.75 (2 H, dd, J = 5.4, 3.1 Hz, **H_{10}**), 7.83 (2 H, dd, J = 5.4, 3.1, Hz, **H_9**); **^{13}C NMR (125 MHz; $CDCl_3$)** δ 17.3 (**C_5**), 20.8 (**C_4**), 21.5 (**C_3**), 29.3 (**C_2**), 76.4 (**C_6**), 99.0 (**C_1**), 123.6 (**C_{10}**), 129.1 (**C_8**), 134.6 (**C_9**), 163.8 (**C_7**); **HRMS** [ES+] $C_{18}H_{17}NO_3Na$ requires 318.1101; found $[M+Na]^+$ 318.1099.

Compound 3: *O*-((*1R,8S,9s*)-bicyclo[6.1.0]non-4-yn-9-ylmethyl)hydroxylamine

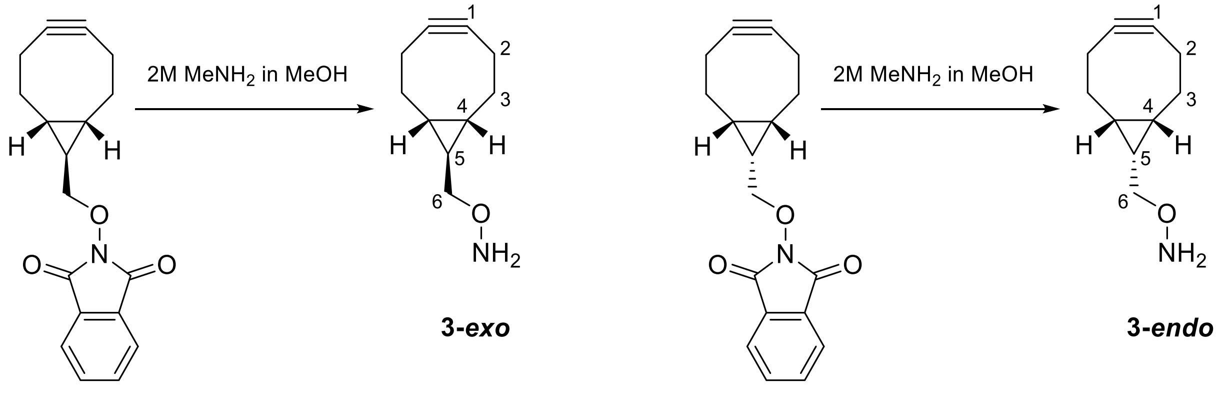

3-*exo*　**3-*endo***

2-*exo*　**2-*endo***

Compound **2-endo** (20 mg, 68 µmol, 1 eq) was added to anhydrous 2 M methanolic methylamine (0.169 ml, 339 µmol, 5 eq). The reaction was shown to be complete by TLC within 2 minutes. The product was diluted in 1:9 EtOAc/Hexane and purified by flash column chromatography (1:9 EtOAc/Hexane) to yield a colourless oil (7.2 mg, 64%).

Endo (isolated product)

R_f 0.25 (3:7 EtOAc/Hexane); **^{1}H NMR** (500 MHz; $CDCl_3$) δ 0.96–0.86 (2 H, m, **H_4**), 1.35–1.25 (1 H, m, **H_5**), 1.62–1.52 (2 H, m, **H_3**), 2.34–2.16 (6 H, m, **H_2, $H_{3'}$**), 3.75 (2 H, d, J = 7.7 Hz, **H_6**), 5.41 (2 H, br. s, NH_2); **^{13}C NMR** (100 MHz; $CDCl_3$) δ 17.6 (C_5), 20.0 (C_4), 21.6 (C_2), 29.4 (C_3), 73.2 (C_6), 99.1 (C_1); **HRMS** [ES+] $C_{10}H_{16}NO$ requires 166.1226; found $[M+H]^+$ 166.1225.

3-*exo* was generated in an analogous way from **2-*exo***, but with evaporation of the crude product mixture for immediate use in situ for subsequent reactions without isolation of the oxyamine.

Exo (deprotection reaction)

R_f 0.64 (50% EtOAc in hexane); **^{1}H NMR (500 MHz; CDCl3)** δ 0.50-0.70 (3H, m, **$H_{4,5}$**), 1.27-1.43 (2H, m, **H_3**), 2.07-2.43 (6H, m, **$H_{2,2',3'}$**), 3.56 (2H, d, *J* = 7.0, **H_6**), 5.22 (2H, bs, **NH_2**); **^{13}C NMR (125 MHz, $CDCl_3$)** δ 21.45 (**C_4**), 22.8 (**C_3**), 23.0 (**C_4**), 23.4 (**C_5**), 33.35 (**C_2**), 80.1 (**C_6**), 98.8 (**C_1**); **HRMS** [ES+] **-** $C_{10}H_{16}NO$ requires 166.1226; found m/z $[M+H]^+$ = 166.1226.

Synthesis of Linker 5

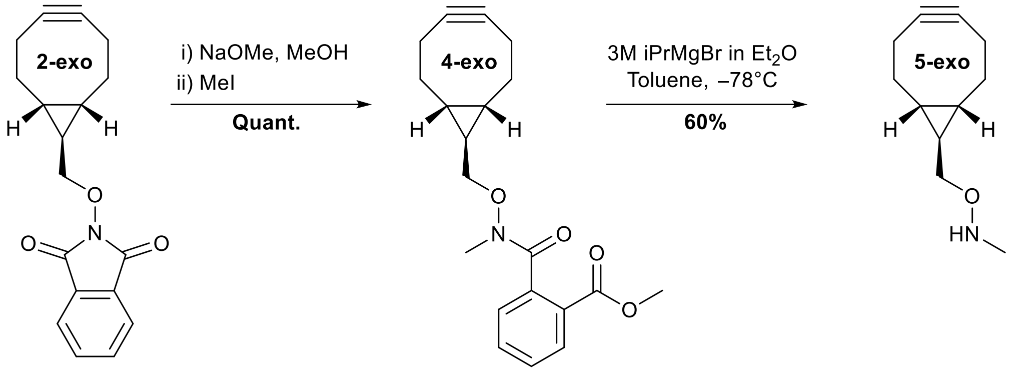

Compound 4-*exo*: Methyl 2-(((1R,8S,9r)-bicyclo[6.1.0]non-4-yn-9-yl)methoxy)carbamoyl)benzoate

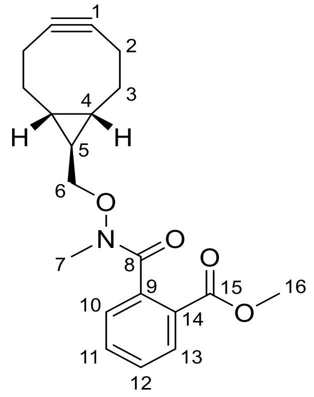

4-*exo*

Compound **2-*exo*** (111 mg, 0.38 mmol, 1eq) was dissolved in MeOH (1 ml). NaOMe (41 mg, 1.14 mmol, 3 eq) was added to the solution and the reaction was stirred for 30 minutes at room temperature until all starting material was observed by TLC (30% EtOAc in hexane) to be consumed. Methyl iodide (100 µl, 1.52 mmol, 4 eq) was added to the solution, and the reaction was monitored by HRMS. Once the methylation reaction was deemed to be complete (≈1 hour), the reaction was quenched with H_2O (5 ml) and the product was extracted with EtOAc (3 x 10 ml). The combined organic layers were dried over $MgSO_4$ and concentrated *in vacuo* to yield compound **4-*exo*** as a yellow residue (129 mg, 100%). **^{1}H NMR** (**500 MHz; $CDCl_3$**) δ 0.64 (2H, bt, **H_4**), 0.89-1.09 (3H, m, **$H_{3,5}$**), 1.81 (2H, bd, **$H_{3'}$**), 2.000-2.07 (2H, m, **H_2**), 2.10-2.20 (2H, m, **$H_{2'}$**), 3.34-3.41(3H, bs, **H_7**), 3.60 (2H, bd, **H_6**, *J* = 7.8 Hz), 3.90 (3H, s, **H_{16}**), 7.40-7.47 (2H, m, **$H_{11,12}$**), 7.67 (1H, dt, *J* = 7.6, 1.0 Hz, **H_{10}**,), 7.97 (1H, d, *J* = 7.6 Hz, **H_{13}**); **^{13}C NMR (125 MHz; $CDCl_3$)** δ 17.05 (**C_5**), 20.4 (**C_4**), 21.8 (**C_2**), 29.0 (**C_3**), 34.65 (**C_7**), 53.0 (**C_{16}**), 71.7 (**C_6**), 99.05 (**C_1**), 127.85 (**C_{11}**), 128.8 (**C_9**), 129.3 (**C_{10}**), 130.1 (**C_{13}**), 132.7 (**C_{12}**), 137.9 (**C_{15}**), 166.6 (**C_8**), 172.1 (**C_{15}**); **HRMS -** $C_{20}H_{23}NO_4$+H requires 342.1699; found $[M+H]^+$ = 342.1697.

Compound 5-*exo*: O-(bicyclo[6.1.0]non-4-yn-9-ylmethyl)-N-methylhydroxylamine

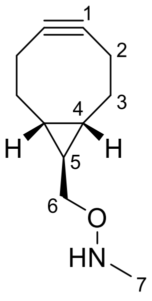

5-*exo*

Compound **4** (50 mg, 0.17 mmol, 1 eq) was dissolved in anhydrous toluene (1 ml) into an oven dried flask under a nitrogen atmosphere. The solution was cooled to -78 °C and a solution of 3M isopropyl magnesium bromide in diethyl ether (0.057 ml, 0.18 mmol, 3 eq) was added dropwise. The reaction was left to stir for 2 hours and allowed to warm to room temperature. The reaction was quenched with H_2O (10 ml) and the product extracted with EtOAc (3×10 ml). The combined organic layers were dried over $MgSO_4$ and concentrated *in vacuo* to yield a crude oil. The crude oil was purified by flash column chromatography eluting in 10% EtOAc in hexane to yield compound **5-*exo*** a clear residue (18 mg, 60%).

R_f - 0.15 (20% EtOAc in hexane); **^{1}H NMR (500 MHz; $CDCl_3$)** δ 0.75-0.89 (2H, m, **H_4**), 1.14-1.28 (1H, m, **H_5**), 1.43-1.63 (2H, m, **H_3**), 2.08-2.30 (6H, m, **$H_{2,2',3'}$**), 2.67 (3H, s, **H_7**), 3.56 (2H, d, *J* = 7.6 Hz, **H_6**); **^{13}C NMR (125 MHz; $CDCl_3$)** δ 17.65 (**C_5**), 19.8 (**C_4**), 21.5 (**C_3**), 29.2 (**C_2**), 39.3 (**C_7**), 70.35 (**C_6**), 98.9 (**C_1**); **HRMS** - $C_{11}H_{17}NO$+H requires 180.1382; found $[M+H]^+$ m/z =180.1388.

Enzymatic hydrolysis of GM1 ganglioside

Compound 10 – GM1 oligosaccharide

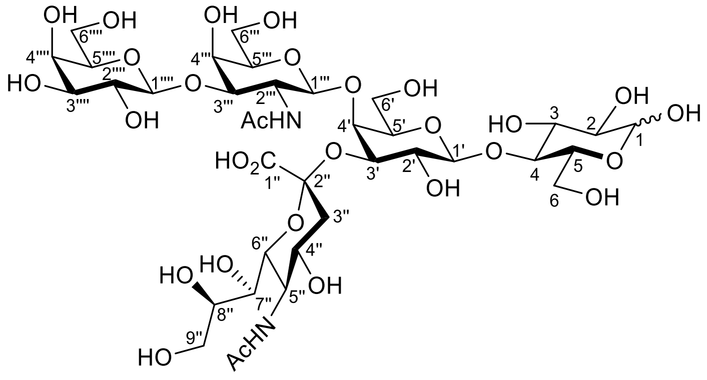

GM1-ceramide (5 mg, 5.01 µmol) was weighed into an Eppendorf tube, and dissolved in 450 µl 50 mM NaOAc buffer, 0.2% v/v Triton X-100 pH 5 in an Eppendorf tube. To the solution, 50 µl of EGCase II (68 µM; see Protein Expression & Purification section page 21) was added and the reaction was left incubating at 37 °C for between 3-5 days until the no GM1-ceramide was no longer observed by TLC (3:2:1 $CHCl_3$:MeOH:H_2O). Once the reaction was complete the solution was washed with diethyl ether

(3 x 30 ml). The aqueous layer was then filtered through a 0.22 μm filter, and the filtrate was then loaded onto a C18 reverse phase cartridge, GM1os was obtained by eluting in H_2O. Fractions containing GM1os were combined and concentrated by lyophilisation to obtain a white solid. Further purification was performed by gel filtration (LH20 or bio-gel P2) to remove any salts. Fractions containing GM1os were once again combined and concentrated by lyophilisation to yield a white solid (3.85 mg, 77%).

NMR data is in agreement with reported data.[2,3]

$\mathbf{R_f}$ – 0.06 (60:40:8 $CHCl_3$:MeOH:H_2O); **^{1}H NMR** (D_2O) δ 2.02 (3H, s, **Ac**), 2.05 (3H, s, **Ac**), 2.65-2.70 (1H, m, $\mathbf{H_{3''equatorial}}$), 3.27-3.31 (1H, m, $\mathbf{H_2}$), 3.35-3.41 (2H, m, $\mathbf{H_{2'',4}}$), 3.49-3.99 (46H, m, **not assigned**), 4.03-4.08 (2H, m, $\mathbf{H_{2'''}}$), 4.12-4.19 (5H, m, $\mathbf{H_{3',4',4''}}$), 4.53 (1H, d, *J* = 7.9 Hz, , $\mathbf{H_{1''''}}$), 4.56 (1H, d, *J* = 7.9 Hz, $\mathbf{H_{1'}}$), 4.69 (1H, d, *J* = 8 .0 Hz, $\mathbf{H_{1\beta}}$), 4.79 (1H, $\mathbf{H_{1'''}}$, lies under D_2O peak) 5.23 (1H, d, $\mathbf{H_{1\alpha}}$, *J* = 5.2 Hz).

Synthesis of BCN glycan derivatives 6, 7 & 11

Compound 6/7: BCN-Lac

6 (*E*)

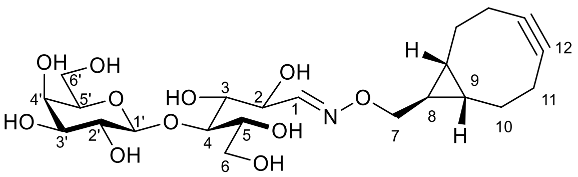

Oxime E Isomer

6 (*Z*)

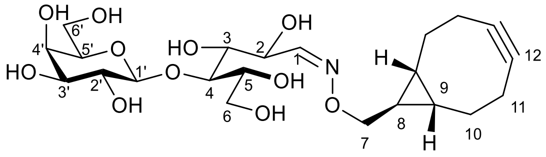

Oxime Z Isomer

7

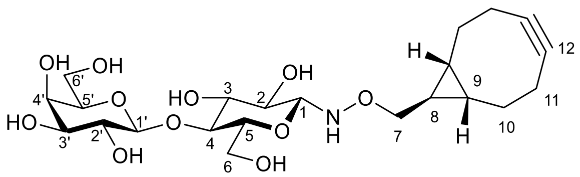

Oxime Ring Closed

Method 1: Oxime ligation in $CHCl_3$:MeOH

Lactose in H_2O (20 μl of 1.5 M stock, final conc. 150 mM) was added to 180 μl of 390 mM linker **3** in 1:1 $CHCl_3$:MeOH (final concentration 351 mM) in a PCR tube. The reaction was heated to 50 °C using a PCR machine with heated lid, and held at 50 °C for 24 hours. The organic solvent was allowed to evaporate, and the crude product was then diluted to 2 ml in water and loaded onto a C18 reverse phase cartridge. The cartridge was then washed with water until no carbohydrate derivatives were detected by orcinol TLC staining. Compounds **6+7** was then eluted from the cartridge in 20% aq. methanol until there was no product observed by orcinol TLC staining. Fractions in which compounds

6+7 were observed were combined and concentrated by lyophilisation to yield compounds **6+7** as a white solid. (10 mg, 69%)

Method 2: Oxime ligation in 1 M NaOAc pH 5

Lactose (80 μl of 1.5M, final concentration 300 mM) was added to 80 μl of 5 M NaOAc pH 5 in a PCR tube, along with 240 μl of 390 mM **3** in 1:1 $CHCl_3$:MeOH was added. The reaction was heated at 50 °C using a PCR machine with heated lid, and held at 50 °C for 24 hours. The crude mixture was then diluted to 2 ml in water and loaded onto a C18 reverse phase cartridge. The cartridge was then washed with water until no carbohydrate derivatives were detected by orcinol TLC staining. Compounds **6+7** was then eluted from the cartridge in 20% aq. methanol until there was no product observed by orcinol TLC staining. Fractions in which compounds **6+7** were observed were combined and concentrated by lyophilisation to yield compounds **6+7** as a white solid. (42.9 mg, 69%) in a ratio of 20:5:14, **6(*E*)**:**6(*Z*)**:**7**.

^{1}H NMR (500 MHz; D_2O) Oxime **6(*E*)** isomer - δ 0.69-0.82 (3H, m, $\mathbf{H_{8,9}}$), 1.33-1.46 (2H, m, $\mathbf{H_{10}}$), 2.16 (2H, bd, $\mathbf{H_{11}}$), 2.28 (2H, bt, $\mathbf{H_{11'}}$), 2.41 (2H, bd, $\mathbf{H_{10'}}$), 3.52-4.13 (12H, m, $\mathbf{H_{2',3,3',4,4',5,5',6,6',7}}$), 4.50 (1H, d, J = 7.8 Hz, $\mathbf{H_{1'}}$), 4.58 (1H, t, J = 6.4 Hz, $\mathbf{H_2}$), 7.66 (1H, d, J = 5.8 Hz, $\mathbf{H_1}$).

Oxime **6(*Z*)** isomer - δ 0.69-0.82 (3H, m, $\mathbf{H_{8,9}}$), 1.33-1.46 (2H, m, $\mathbf{H_{10}}$), 2.16 (2H, bd, $\mathbf{H_{11}}$), 2.28 (2H, bt, $\mathbf{H_{11'}}$), 2.41 (2H, bd, $\mathbf{H_{10'}}$), 3.52-4.13 (12H, m, $\mathbf{H_{2',3,3',4,4',5,5',6,6',7}}$), 4.45 (1H, d, J = 7.8 Hz, $\mathbf{H_{1'}}$) 4.99 (1H, dd, J = 5.3, 1.3 Hz, $\mathbf{H_{2,}}$), 6.97 (1H, d, J = 5.3 Hz, $\mathbf{H_{1,}}$).

Ring closed **7** - δ 0.69-0.82 (3H, m, $\mathbf{H_{8,9}}$), 1.33-1.46 (2H, m, $\mathbf{H_{10}}$), 2.16 (2H, bd, $\mathbf{H_{11}}$), 2.28 (2H, bt, $\mathbf{H_{11'}}$), 2.41 (2H, bd, $\mathbf{H_{10'}}$), 3.41 (1H, t, J = 9.0 Hz, $\mathbf{H_{2,}}$), 3.52-4.13 (12H, m, $\mathbf{H_{2',3,3',4,4',5,5',6,6',7}}$), 4.31 (1H, d, J = 8.9 Hz, $\mathbf{H_1}$), 4.54 (1H, d, J = 7.6 Hz, $\mathbf{H_{1'}}$).

^{13}C NMR (125 MHz; D_2O) δ 20.7, 22.35, 22.5, 22.5, 22.6, 22.6, 23.15 ($\mathbf{C_{11,10,9,8}}$), 32.7 ($\mathbf{C_7}$), 57.8, 60.9, 61.0, 62.1, 68.5, 68.5, 68.6, 69.3, 69.3, 71.0, 71.05, 71.2. 72.5, 72.6, 75.1, 75.4, 75.4, 75.95, 78.1, 78.3, 78.4, 79.3 ($\mathbf{C_{2,2',3,3',4,4',5,5',6,6'}}$), 90.1 ($\mathbf{C_{1(C)}}$), 103.8 ($\mathbf{C_{12}}$), 102.9 ($\mathbf{C_{1'(A)}}$), 130.0 ($\mathbf{C_{1'(B,C)}}$), 151.7 ($\mathbf{C_{1(A,B)}}$).

HRMS - $C_{22}H_{35}NO_{11}$+H requires 490.2282. Measured m/z $[M+H]^+$ = 490.2282.

<u>**Compound 8 - Lac-NMe-BCN**</u>

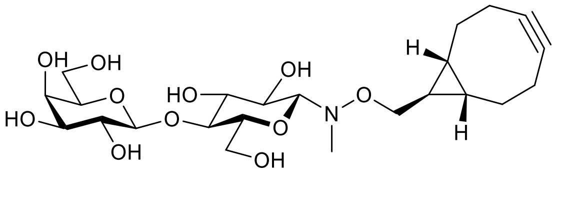

Lactose (20 μl of a 1 M stock in H_2O, final conc-400 mM) was added to 10 μl of 5 M NaOAc buffer pH 5 in a PCR tube, followed by 20 μl of a 1M solution of **5** in $CHCl_3$ (final concentration 400 mM). The reaction was heated to 50 °C using a PCR machine with heated lid and held at 50 °C for 48 hours. The

crude mixture was then diluted to 500 μl in ddH_2O and loaded onto a C18 reverse phase cartridge. The cartridge was then washed with water until no carbohydrate derivatives were detected by orcinol TLC staining. Compound **8** was then eluted from the cartridge in 50% aq. methanol until there was no product observed by orcinol TLC staining. Fractions in which compound **8** were observed were combined and concentrated by lyophilisation to yield compound **8** as a white solid. (1.6 mg, 40%).

HRMS - $C_{23}H_{37}NO_{11}$+H requires 504.2445. Measured m/z $[M+H]^+$ = 504.2445.

<u>**Compound 11 – GM1-BCN**</u>

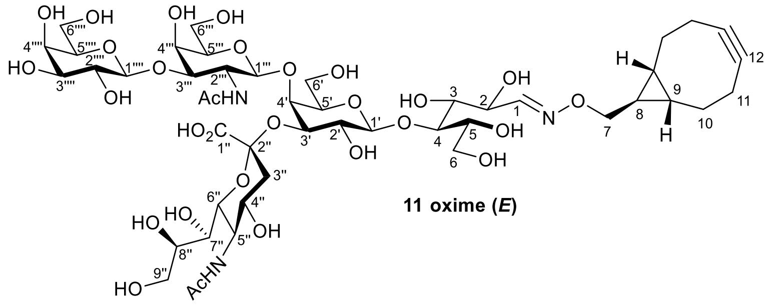

11 oxime (*E*)

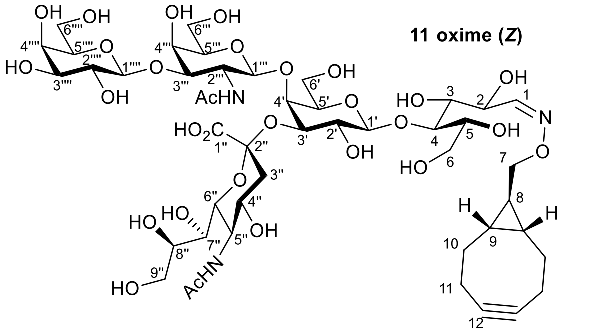

11 oxime (*Z*)

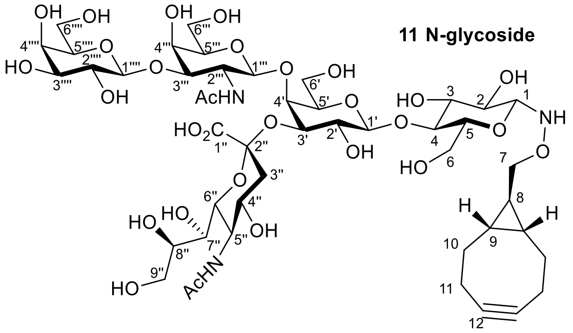

11 N-glycoside

<u>Method 1: Oxime ligation in $CHCl_3$:MeOH</u>

GM1os (1 mg, 1 μmol, 1 eq) was suspended in 1:1 $CHCl_3$:MeOH (2 μl) in a 100 μL PCR tube. Linker **3-exo** (3 μl of 390 mM stock solution in 1:1 $CHCl_3$:MeOH) was added to this suspension and the reaction was heated at 50 °C for 48 hr using a PCR thermocycler with a heated lid (105 °C). Completion of the reaction was confirmed by TLC (2:2:1 BuOH:MeOH:H_2O). The crude mixture was then diluted to 100 μl in water and loaded onto a C18 reverse phase cartridge, and the cartridge was then washed with water until no carbohydrate derivatives were detected by orcinol TLC staining. Compound **11** was then eluted from the cartridge in 20% aq. methanol until there was no product observed by orcinol TLC staining. Fractions in which **11** was observed were combined and concentrated by lyophilisation to yield compound **11** as a white solid comprising a mixture of open chain and cyclised isomers. (0.38 mg, 33%)

<u>Method 2: Oxime ligation in 1 M NaOAc pH 5</u>

GM1os (1 mg, 1 μmol, 1 eq) was dissolved in H_2O (2 μl) in a 100 μL PCR tube, to which NaOAc buffer (1 μl of a 5 M stock solution, pH 5) and linker **3-exo** (2 μl of a 390 mM stock solution in 1:1 $CHCl_3$:MeOH)

were added, and the reaction was heated at 50 °C for 48 hr using a PCR thermocycler with a heated lid (105 °C).Completion of the reaction was confirmed by TLC (2:2:1 BuOH:MeOH:H_2O). The crude mixture was diluted to 100 µl in H_2O and loaded onto a C18 reverse phase cartridge, the cartridge was then washed with H_2O until no carbohydrate derivatives were detected by orcinol TLC staining. Compound **11** was eluted from the cartridge in 20% aq. methanol until there was no product observed by orcinol TLC staining. Fractions in which compound **11** was observed were combined and concentrated by lyophilisation to yield **11** as a white solid comprising a mixture of open chain and cyclised isomers. (0.28 mg, 24%).

R$_f$ – 0.63 (2:2:1 BuOH:MeOH:H_2O); **^{1}H NMR (500 MHz; D_2O)** δ 0.71-0.84 (m, **H$_{8,9}$**), 1.36-1.46 (m, **H$_3$**), 1.93 (t, 11.8 Hz, **H$_{3''ax}$**), 2.00-2.05 (m, **Ac**), 2.12-2.21 (m, **H$_2$**), 2.25-2.35 (m, **H$_{2'}$**), 2.40-2.47 (m, **H$_3$**), 2.64-2.68 (m, **H$_{3''eq}$**), 3.29-4.19 (m, other sugar ring protons), 4.30-4.32 (m, **H$_{1\ N\text{-}glycoside}$**), 4.48-4.59 (m, **H$_{1'',1''''}$**, **H$_{2\ E\text{-}oxime}$**), 4.74 (m, **H$_{1'''}$**), 4.93-4.99 (m, **H$_{2\ Z\text{-}oxime}$**) 6.93 (1H, d, 5.4 Hz, **H$_{1\ Z\text{-}oxime}$**), 6.97 (1H, d, 4.9 Hz, **H$_{1ep\ Z\text{-}oxime}$**), 7.48 (1H, d, 6.9 Hz, **H$_{1ep\ E\text{-}oxime}$**), 7.59 (1H, d, 5.9 Hz, **H$_{1\ E\text{-}oxime}$**); **HRMS –** $C_{47}H_{75}N_3O_{29}$+H requires 1146.4564. Measured m/z $[M+H]^+$ = 1146.4554.

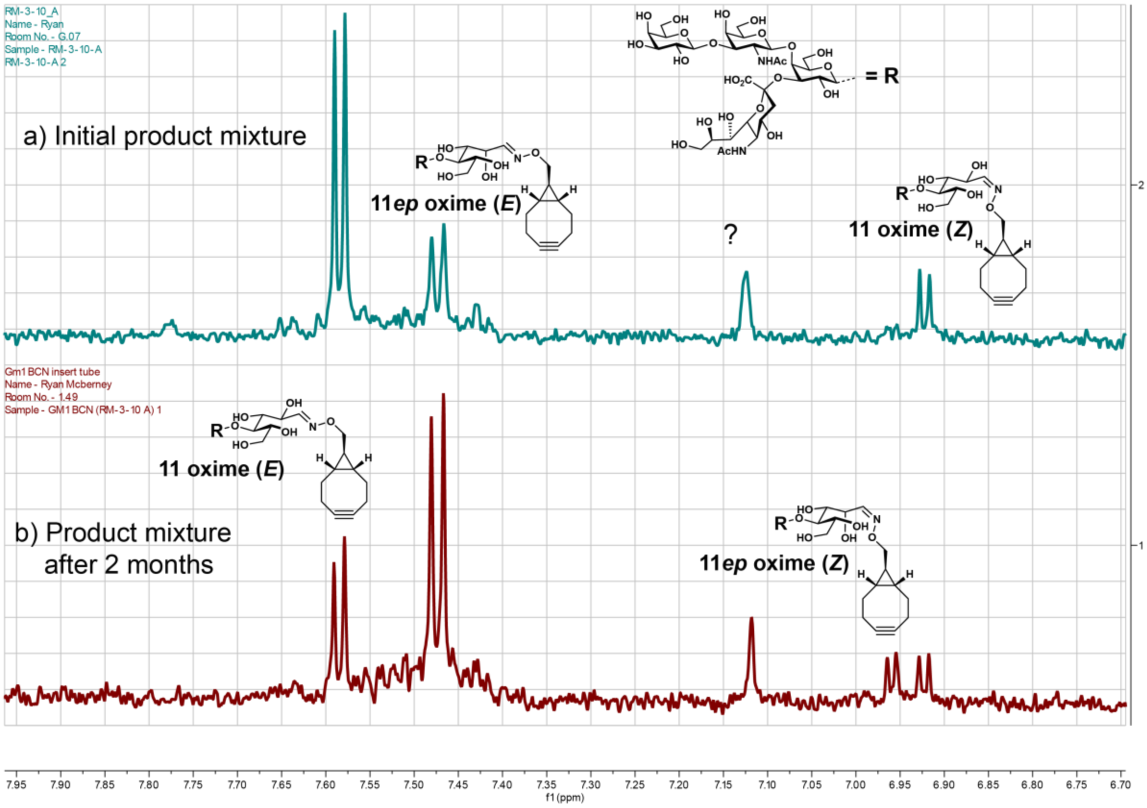

Figure S0. Oxime region of the ^{1}H NMR spectra of (a) the initial product mixture, and (b) the product mixture after 2 months. Additional oxime proton signals were observed for the GM1os-derived product. Initially, three doublets were observed at the end of the 48 h reaction at 50 °C, but over the course of 2 months, the intensities of these signals changed and a fourth doublet appeared at 6.97

ppm. As other NMR spectral features (relative integrations of the combined oxime and N-glycoside peaks, galactosyl anomeric peaks, NHAc peaks, Sialic acid H3eq, H3ax) and mass spectrometry data indicated the compounds remained intact, we tentatively assign the oxime peaks that grew over time as the manno-configured C-2 epimers of compound **11**.

Synthesis of glycosyl azides 9 & 13

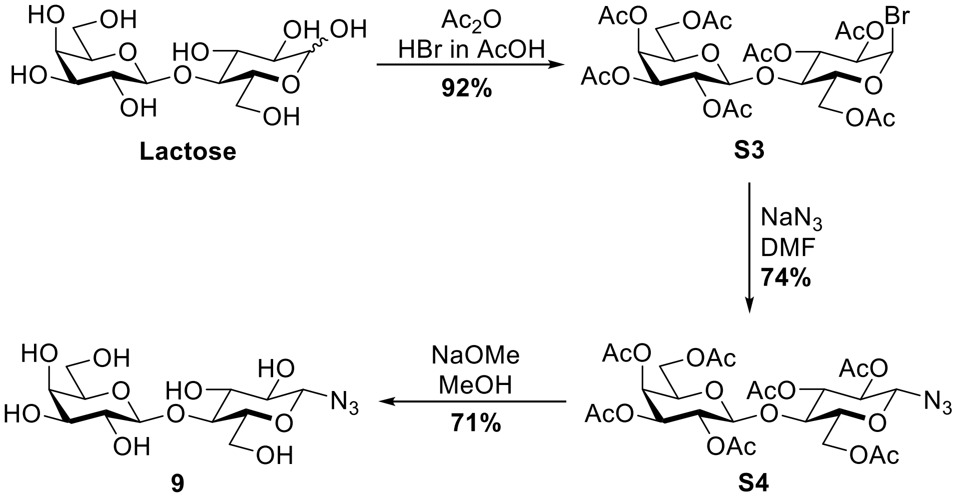

Compound S3: 2,3,6-Tri-O-acetyl-4-(tetra-O-acetyl-b-d-galactopyranosyl)-a-d-glucopyranosyl bromide[4]

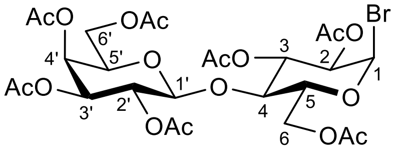

Lactose (5.00 g, 14.6 mmol, 1.0 eq) was added to acetic anhydride (17.0 mL, 175 mmol, 12.0 eq) under N_2, followed by 33% (*w/v*) HBr in AcOH (3.3 mL, 18.4 mmol, 1.2 eq) and stirred at room temperature. After 1 hours, HBr in AcOH (17.0 mL, 94.8 mmol, 6.2 eq) was added and the reaction stirred at room temperature for a further 3 hours. The solvent was removed *in vacuo* whilst ensuring the mixture did not exceed 30 °C. The resulting orange solid was redissolved in DCM (100 mL) and washed with H_2O (50 mL), sat. aq. $NaHCO_3$ (50 mL) and brine (50 mL). The organic layer was dried over $MgSO_4$, filtered and concentrated *in vacuo* to yield crude **S3** as a white solid (9.39 g, 92%).

NMR data is in agreement with reported data.[4]

$\mathbf{R_f}$ – 0.70 (3:7 EtOAc/DCM); **^{1}H NMR** (500 MHz; $CDCl_3$) δ 6.51 (1 H, d, J = 4.1 Hz, $\mathbf{H_1}$), 5.54 (1 H, t, J = 9.7 Hz, $\mathbf{H_3}$), 5.34 (1 H, dd, J = 3.5, 0.9 Hz, $\mathbf{H_{4'}}$), 5.11 (1 H, dd, J = 10.4, 8.0 Hz, $\mathbf{H_{2'}}$), 4.94 (1 H, dd, J = 10.4, 3.5 Hz, $\mathbf{H_{3'}}$), 4.74 (1 H, dd, J = 9.9, 4.1 Hz, $\mathbf{H_2}$), 4.50 (1 H, d, J = 7.9 Hz, $\mathbf{H_{1'}}$), 4.48 (1 H, d, J = 10.0 Hz, $\mathbf{H_{6a}}$), 4.21–4.04 (4 H, m, $\mathbf{H_5}$, $\mathbf{H_{6b}}$, $\mathbf{H_{6a'b'}}$), 3.90–3.82 (2 H, m, $\mathbf{H_4}$, $\mathbf{H_{5'}}$), 2.15 (3 H, s, Me), 2.12 (3 H, s, Me), 2.08

(3 H, s, Me), 2.05 (3 H, s, Me), 2.05 (3 H, s, Me), 2.04 (3 H, s, Me), 1.94 (3 H, s, Me); **^{13}C NMR** (125 MHz; $CDCl_3$) δ 170.5 (C=O), 170.3 (C=O), 170.3 (C=O), 170.2 (C=O), 170.1 (C=O), 169.3 (C=O), 169.1 (C=O), 100.9 ($C_{1'}$), 86.5 (C_1), 75.1 (C_4), 73.1 (C_5), 71.1 ($C_{3'}$), 71.0 (C_2), 70.9 ($C_{5'}$), 69.7 (C_3), 69.1 ($C_{2'}$), 66.7 ($C_{4'}$), 61.2 (C_6), 61.0 ($C_{6'}$), 20.9 (Me), 20.9 (Me), 20.8 (Me), 20.8 (Me), 20.8 (Me), 20.8 (Me), 20.6 (Me); **HRMS** [ES+] found $[M+Na]^+$ 721.0958, $C_{26}H_{35}BrO_{17}Na$ requires 721.0950.

Compound S4: 2,3,6-Tri-O-acetyl-4-(tetra-O-acetyl-β-D-galactopyranosyl)-β-D-glucopyranosyl azide[5]

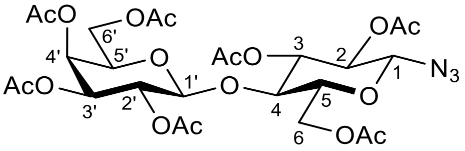

Peracetylated lactosyl bromide **S3** (9.2 g, 13.2 mmol, 1.0 eq) was dissolved in anhydrous DMF (120 mL). To the solution NaN_3 (0.94 g, 14.5 mmol, 1.1 eq) was added and the reaction stirred at room temperature for 16 hours. The reaction was diluted with DCM (400 mL) and washed with $NaHCO_3$ (3 × 200 mL), H_2O (200 mL), then brine (200 mL). The organic layer was dried over $MgSO_4$, filtered, concentrated *in vacuo* and purified by flash column chromatography (1:1 EtOAc/Hexane) to yield **S4** as a white crystalline solid (6.42 g, 74%).

NMR data is in agreement with reported data.[5]

R_f – 0.24 (1:1 EtOAc/Hex); **^{1}H NMR** (500 MHz; $CDCl_3$) δ 5.32 (1 H, dd, J = 3.5, 1.1 Hz, **$H_{4'}$**), 5.17 (1 H, t, J = 9.4 Hz, **H_3**), 5.06 (1 H, dd, J = 10.4, 7.9 Hz, **$H_{3'}$**), 4.93 (1 H, dd, J = 10.4, 3.5 Hz, **$H_{2'}$**), 4.82 (1 H, t, J = 9.0 Hz, **H_2**), 4.61 (1 H, d, J = 8.8 Hz, **H_1**), 4.47 (1 H, dd, J = 12.1, 2.1 Hz, **$H_{6a'}$**), 4.46 (1 H, d, J = 8.0 Hz, **$H_{1'}$**), 4.12–4.02 (3 H, m, **$H6_{ab}$, $H_{6b'}$**), 3.85 (1 H, td, J = 6.5, 1.0 Hz, **$H_{5'}$**), 3.79 (1 H, t, J = 9.5 Hz, **H_4**), 3.68 (1 H, ddd, J = 10.0, 5.0, 2.0 Hz, **H_5**), 2.12 (3H, s, Me), 2.10 (3H, s, Me), 2.04 (3H, s, Me), 2.03 (3H, s, Me), 2.01 (3H, s, Me), 2.01 (3H, s, Me), 1.93 (3H, s, Me); **^{13}C NMR** (125 MHz; $CDCl_3$) δ 170.4 (C=O), 170.4 (C=O), 170.2 (C=O), 170.1 (C=O), 169.7 (C=O), 169.6 (C=O), 169.1 (C=O), 101.2 ($C_{1'}$), 87.7 (C_1), 75.8 (C_4), 74.9 (C_5), 72.6 (C_3), 71.0 ($C_{2'}$), 71.0 (C_2), 70.8 ($C_{5'}$), 69.1 ($C_{3'}$), 66.7 ($C_{4'}$), 61.8 ($C_{6'}$), 60.9 (C_6), 20.9 (Me), 20.8 (Me), 20.7 (Me), 20.7 (Me), 20.7 (Me), 20.7 (Me), 20.6 (Me); **HRMS** [ES+] found $[M+Na]^+$ 684.1879, $C_{26}H_{35}N_3O_{17}Na$ requires 684.1859.

Compound 9: 4-(β-D-galactopyranosyl)-β-D-glucopyranosyl azide[5]

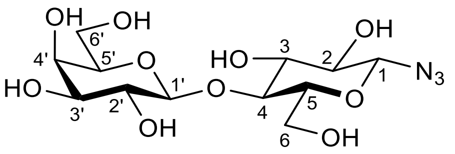

Peracetylated lactosyl azide **S4** (5.82 g, 8.8 mmol, 1.0 eq) was dissolved in MeOH (40 mL) and NaOMe (3.8 g, 70.4 mmol, 8.0 eq) was added. Once TLC confirmed the reaction had reached completion (~ 1 hour), washed DOWEX-50 H^+ was added until the reaction was neutral. The resin was removed by vacuum filtration through celite and the solution concentrated *in vacuo*. The resulting oil was redissolved in H_2O (20 mL) and lyophilised to yield crude **4.8**. The crude lactosyl azide **4.8** was purified by size exclusion chromatography using Biogel P2 column equilibrated with 20 mM ammonium formate. β-lactosyl azide **9** was obtained as a white foam (2.29 g, 71%).

NMR data is in agreement with reported data.[5]

R$_f$ – 0.20 (6:4:0.8 $CHCl_3$/MeOH/H_2O); **^{1}H NMR** (500 MHz; $CDCl_3$) δ 4.77 (1 H, d, J = 9.2 Hz, **H$_1$**), 4.45 (1 H, d, J = 7.7 Hz, **H$_{1'}$**), 4.00 (1 H, dd, J = 12.4, 1.6 Hz, **H$_{6a}$**), 3.92 (1 H, dd, J = 3.4, 0.6 Hz, **H$_{4'}$**), 3.86–3.64 (8 H, m, **H$_3$, H$_{3'}$, H$_4$, H$_5$, H$_{5'}$, H$_{6b}$, H$_{6a'b'}$**), 3.54 (1 H, dd, J = 10.0, 7.7 Hz, **H$_{2'}$**), 3.35–3.29 (1 H, m, **H$_2$**); **^{13}C NMR** (125 MHz; $CDCl_3$) δ 102.9 ($C_{1'}$), 89.9 (C_1), 77.7 (C_4), 76.7 (C_5), 75.3 ($C_{5'}$), 74.3 (C_3), 72.5 (C_2), 72.5 ($C_{3'}$), 70.9 ($C_{2'}$), 68.5 ($C_{4'}$), 61.0 (C_6), 59.8 ($C_{6'}$); **HRMS** [ES+] found $[M+Na]^+$ 390.1123, $C_{12}H_{21}N_3O_{10}Na$ requires 390.1119.

Compound 13 - β-GM1 azide

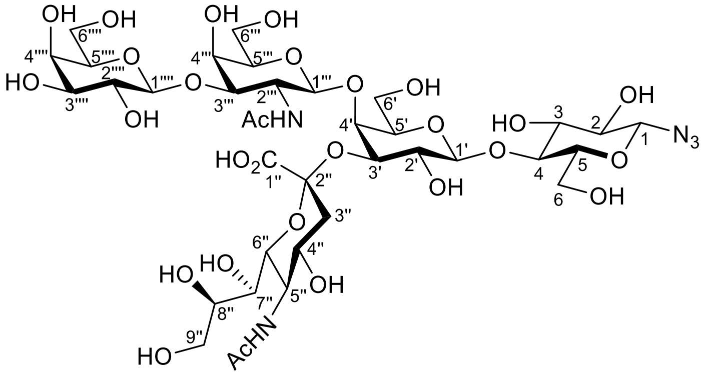

GM1os (9 mg, 9 µmol, 1 eq), DMC (15 mg, 90 µmol, 10 eq) and NaN_3 (25 mg, 400 µmol, 44 eq) were dissolved in H_2O (0.1 mL) in an Eppendorf tube. TEA (22.5 µL, 161 µmol, 18 eq) was added and mixed by vortex and left at 37 °C for 48 hours. The product was isolated by size exclusion using Biogel P2 column equilibrated with 20 mM ammonium formate to yield β-GM1 azide as a white foam after lyophilisation (8.6 mg, ca. 90% conversion to azide product with ca.10% hemiacetal remaining).

R$_f$ 0.58 (2:2:1 n-BuOH/MeOH/H_2O); **^{1}H NMR** (500 MHz; D_2O; 275K) δ 1.94 (1 H, t, J = 12.3 Hz, **H$_{3''axial}$**), 1.99 (3 H, s, **Ac**), 2.01 (3H, s, **Ac**), 2.63 (1 H, dd, J = 12.3, 4.5 Hz, **H$_{3''equatorial}$**), 3.34 (1 H, dd, J = 9.3, 8.2

Hz, **H_2**), 3.53-3.45 (2 H, m, **$H_{3''}$, H_4**), 3.99–3.54 (24 H, m, **not assigned**), 4.01 (1 H, dd, J = 10.8, 8.7 Hz, **$H_{3'}$**), 4.19–4.11 (3 H, m, **$H_{4'}$, $H_{2'''}$, $H_{4'''}$**), 4.52 (1 H, d, J = 7.8 Hz, **$H_{1''''}$**), 4.53 (1 H, d, J = 7.7 Hz, **$H_{1'}$**), 4.75 (1 H, d, J = 8.8 Hz, **$H_{1'''}$**), 4.78 (1 H, d, J = 8.8 Hz, **$H_{1\beta}$**); **HRMS –** $C_{37}H_{61}N_5O_{28}Na$ requires 1046.3401. Measured m/z $[M+Na]^+$ = 1046.3417.

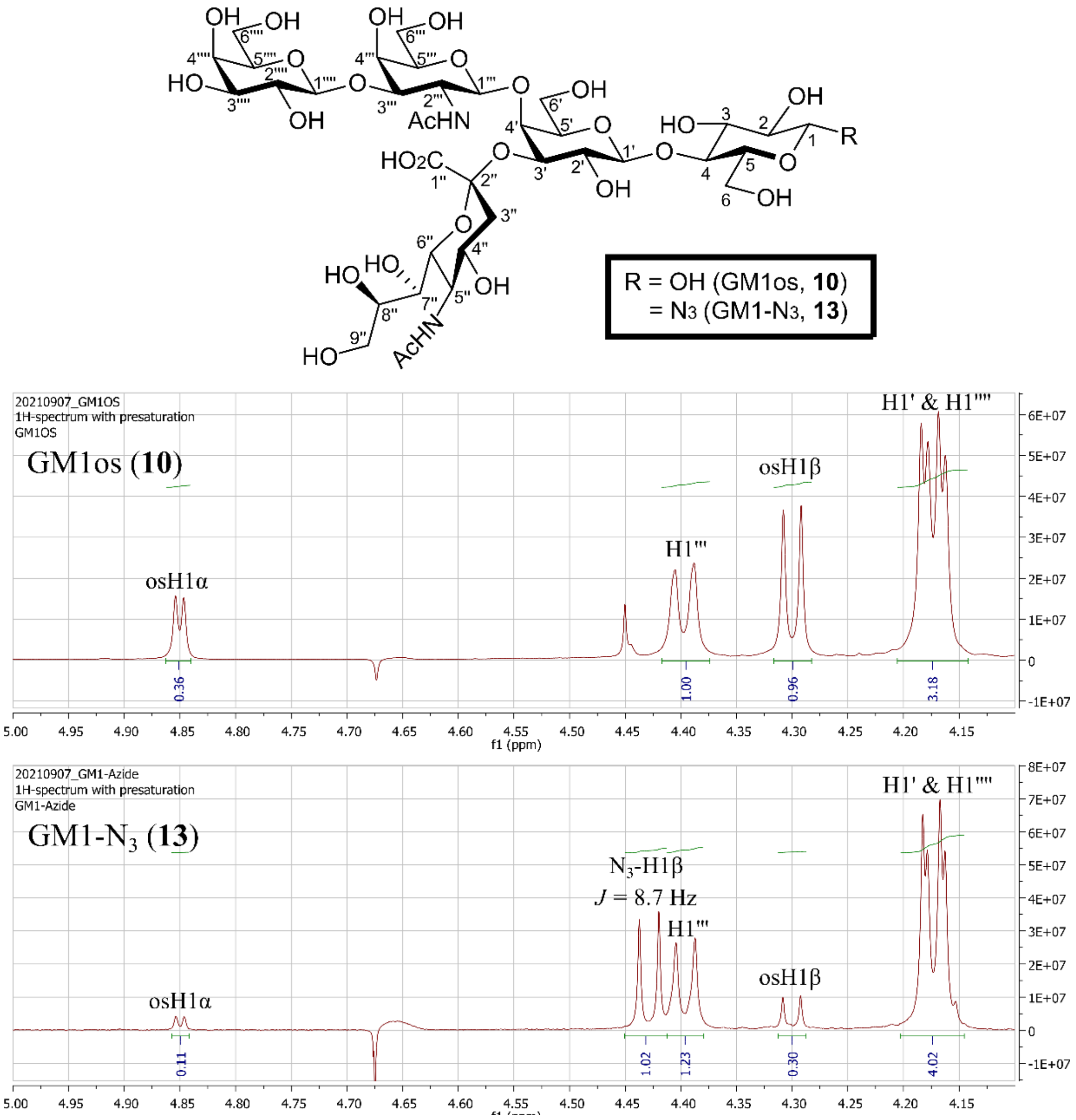

Figure S1. Expansion of the anomeric region of NMR spectra for GM1os (top, **10**) and GM1 azide (bottom, **13**). Spectra shown appearance of new peak for Glu N_3-H1β anomeric centre and overall reduction in signal intensity for peaks corresponding to osH1α/β compared to other anomeric centres (H1’, H1’’’, & H1’’’’). NMR performed at 275 K to shift residual H_2O peak to ~5.0 ppm; residual H_2O peak supressed via presaturation. While caution should be exercised when interpreting signal integrations from the presaturation experiment, the spectra are consistent with most of the GM1os being converted to the azide product.

Synthesis of azidohomoalanine

Azidohomoalanine was prepared according to reports by Lau *et al.*[6]. Data obtained was in support of the reported literature values.

Protein Expression & Purification

Buffers and Media

PBS buffer (pH 7.4): 10 mM Na_2HPO_4, 1.8 mM KH_2PO_4, 137 mM NaCl, 2.7 mM KCl.

PBS-T (pH 7.4): 10 mM Na_2HPO_4, 1.8 mM KH_2PO_4, 137 mM NaCl, 2.7 mM KCl, 0.1% (v/v) Tween-20, 0.1% (v/v) Bovine serum album.

Phosphate buffer (pH 7.2): 100 mM Sodium phosphate, 100 mM NaCl

Phosphate buffer, high-salt (pH 7.2): 50 mM Sodium phosphate, 500 mM NaCl

Lysogeny broth LB: 1% w/v tryptone, 0.5% w/v yeast extract, 1% w/v NaCl.

TYP broth: 1.6 g/L tryptone, 1.6 g/L yeast, 5 g/L NaCl, 2.5 g/L K_2HPO_4

Table S1. New minimal media (NMM)[7]

**Component**	**Final Concentration**
$(NH_4)SO_4$	7.5 mM
NaCl	8.5 mM
K_2HPO_4	22 mM
KH_2PO_4	50 mM
$MgSO_4$	1 mM
$CaCl_2$	1 mg/L
$FeCl_2$	1 mg/L
Canonical amino acids (excluding methionine) – note 1	50 mg/L each
Glucose	20 mM
Methionine	70 µM
Trace elements ($CuSO_4$, $ZnCl_2$, $MnCl_2$, $(NH_4)MoO_4$)	10 µg/L
Thiamine	10 mg/L
Biotin (note 2)	10 mg/L

Note 1: Required warming to 30 °C with stirring to dissolve.

Note 2: Addition of aq. NaOH was required to dissolve biotin

Plasmids

All genes were based on the sequence for El Tor CTB and expressed in plasmids derived from pMAL-p5x using a LTIIb periplasmic targeting leader sequence.

Table S2. A summary of the plasmids and mutant proteins coded for by each plasmid.

Plasmid identity	Mutant protein and its **descriptor**(s) in the current paper	reference
pTRB-W88E	CTB(W88E)	Branson *et al.*[8]
pSAB2.3-azido	CTB(M37L-M68L-M101L-K43M)	Haigh *et al.*[9]
pSAB2.4	CTB(M37L-M68L-M101L-K43M-W88E) aka **Met-W88E**; **N_3-W88E**	current paper

Sequence alignments

WT El Tor CTB (residues 1-52)	TPQNITDLCAEYHNTQIYTLNDKIFSYTESLAGKREMAIITFKNGAIFQVEV
**CTB(W88E)** (residues 1-52)	TPQNITDLCAEYHNTQIYTLNDKIFSYTESLAGKREMAIITFKNGAIFQVEV
**CTB(Met, W88E)** (residues 1-52)	TPQNITDLCAEYHNTQIYTLNDKIFSYTESLAGKRELAIITFMNGAIFQVEV
WT El Tor CTB (residues 53-103)	PGSQHIDSQKKAIERMKDTLRIAYLTEAKVEKLCVWNNKTPHAIAAISMAN
**CTB(W88E)** (residues 53-103)	PGSQHIDSQKKAIERMKDTLRIAYLTEAKVEKLCVENNKTPHAIAAISMAN
**CTB(Met, W88E)** (residues 53-103)	PGSQHIDSQKKAIERLKDTLRIAYLTEAKVEKLCVENNKTPHAIAAISLAN

Site directed Mutagenesis PCR

The primers used to introduce the W88E mutation into plasmid pSAB2.3-azido to create pSAB2.4 are shown below. Bases which were non-complementary to the template plasmid DNA are shown in red. Predicted T_m = 80.4 °C.

5'- GTCGAAAAGTTATGTGTA**GA**GAATAATAAAACGCCTCATGCGATTGCCG -3'

3'- CAGCTTTTCAATACACAT**CT**CTTATTATTTTGCGGAGTACGCTAACGGC -5'

Novogen enzymes and buffers were used, and all products were analysed by nucleic acid electrophoresis). Standard PCR conditions were set up in 50 µl reactions on ice, containing the following:

1 x KOD hot start buffer

140 nM forward primer

140 nM reverse primer

200 µM dNTPs

50 ng DNA template

1 unit KOD polymerase

1.5 µM $MgSO_4$

The solutions were made up to 50 µl with dd H_2O and subjected to the following PCR program:

20 cycles of: 2 minutes @ 95 °C; 20 seconds @ 95 °C; 20 seconds @ 55 °C; 2.5 minutes @ 70 °C; 10 minutes @ 72 °C

Restriction digest was carried out using DpnI restriction enzyme from New England Biolabs (NEB). DNA digestions were set up to a final volume of 20 µl, with approximately 1 unit of enzyme per µg of DNA. The digests were analysed by nucleic acid electrophoresis, following incubation for two hours at 37 °C.

Expression and purification of non-binding mutant W88E

A glycerol stock of BL21 Gold (DE3) cells containing the W88E mutant plasmid pTRB-W88E was used to inoculate 2 x 5 ml of LB media containing 100 µg/ml ampicillin. These cultures were incubated at 30 °C overnight, shaking at 160 rpm and then used to inoculate 4 x 1 L of LB media containing 100 µg/ml of ampicillin. These cultures were grown at 37 °C until an OD_{600} 0.6-0.8 was reached. Protein expression was induced by the addition of 0.5 ml IPTG (1M stock), and the cells were incubated for a further 20 hours at 30 °C. Cells were pelleted by centrifugation at 13,000 x *g* for 20 minutes, after which the supernatant was retained, and the pellet discarded.

Proteins from the supernatant were isolated by precipitation by the addition of solid ammonium sulfate to saturation (57% w/v), and the saturated solution was stirred for a further 3 hours at room temperature. The solution was then centrifuged at 13,000 x *g* for 30 minutes, the supernatant was then discarded, and the pellet was re-suspended in PBS pH 7.2. The suspension was centrifuged at 4000 rpm for 10 minutes, and sequentially filtered through 0.8 and 0.22 µm filters. The protein was purified by Ni-NTA affinity chromatography washing with PBS (no imidazole). CTB has native surface histidine residue meaning the pentamer is able to bind to Ni-NTA resin without the presence of a His-tag. The protein was then eluted from the column by washing with PBS containing 200 mM imidazole, before further purification by size exclusion chromatography on Superdex S75 in PBS.

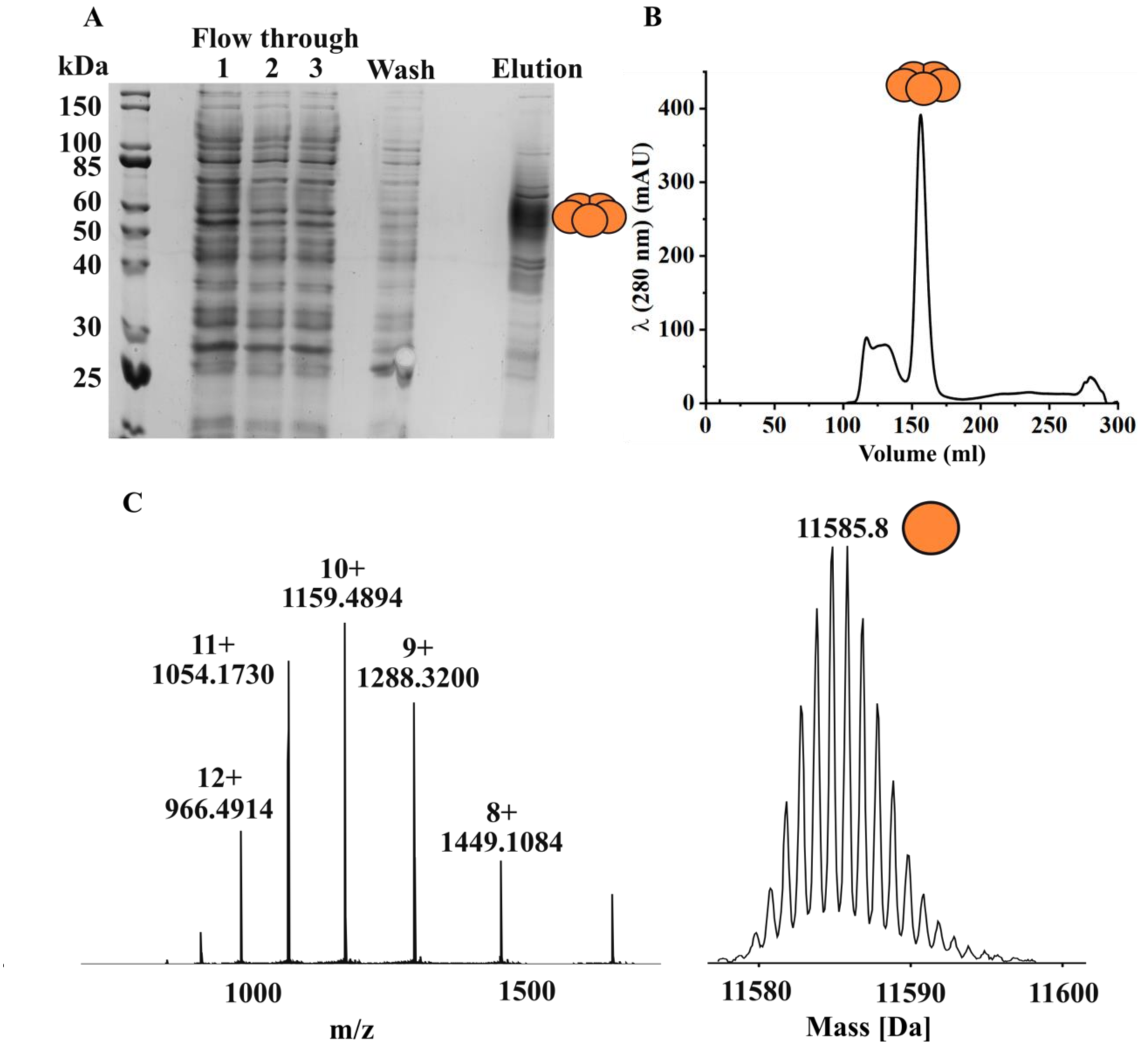

Figure S2. A) SDS-PAGE analysis of the Ni-NTA purification of W88E (samples were not boiled prior to loading onto the gel so that the protein would migrate as a pentamer – position of which is indicated by the orange cartoon). B) Size exclusion chromatogram of W88E sample obtained after elution from the Ni-NTA column. The peak corresponding to the intact pentamer is indicated. C) HRMS of W88E following size exclusion chromatography, with both the charge state and deconvoluted spectra shown. (Expected mass – 11585.9 Da. Calculated using UCSF prospector MS tool)

__Expression and purification of CTB(M37L-M68L-M101L-K43M-W88E) aka Met-W88E__

E.coli BL21 (DE3) cells transformed with plasmid pSAB2.4 harbouring the gene for Met-W88E, were used to inoculate 2 x 5 ml LB containing 100 μg/ml ampicillin). The starter cultures were incubated at 37 °C with shaking at 200 rpm overnight and then used to inoculate 2 x 100 ml of LB media containing 100 μg/ml of ampicillin. The cultures were incubated at 37 °C until an $O.D_{600}$ of 0.6 was reached. Expression was induced by addition of IPTG (final concentration 0.5 mM), and the cultures were left shaking at 30 °C overnight. The cells were harvested by centrifugation at 13,000 x *g* for 30 minutes. The cells were resuspended in 4 ml PBS pH 7.2 and lysed using 0.4 ml BugBuster (10x lysis reagent).

The suspension was agitated on a rocker at room temperature for 40 minutes. The cell fragments were pelleted by centrifugation at 13,000 x *g* for 10 minutes, and the pellet discarded. The protein was isolated using Ni-NTA chromatography, washing in PBS pH 7.2 and eluting in PBS pH 7.2 containing 200 mM imidazole. The protein was further purified by size exclusion chromatography using a Superdex 75 16/60 column in PBS.

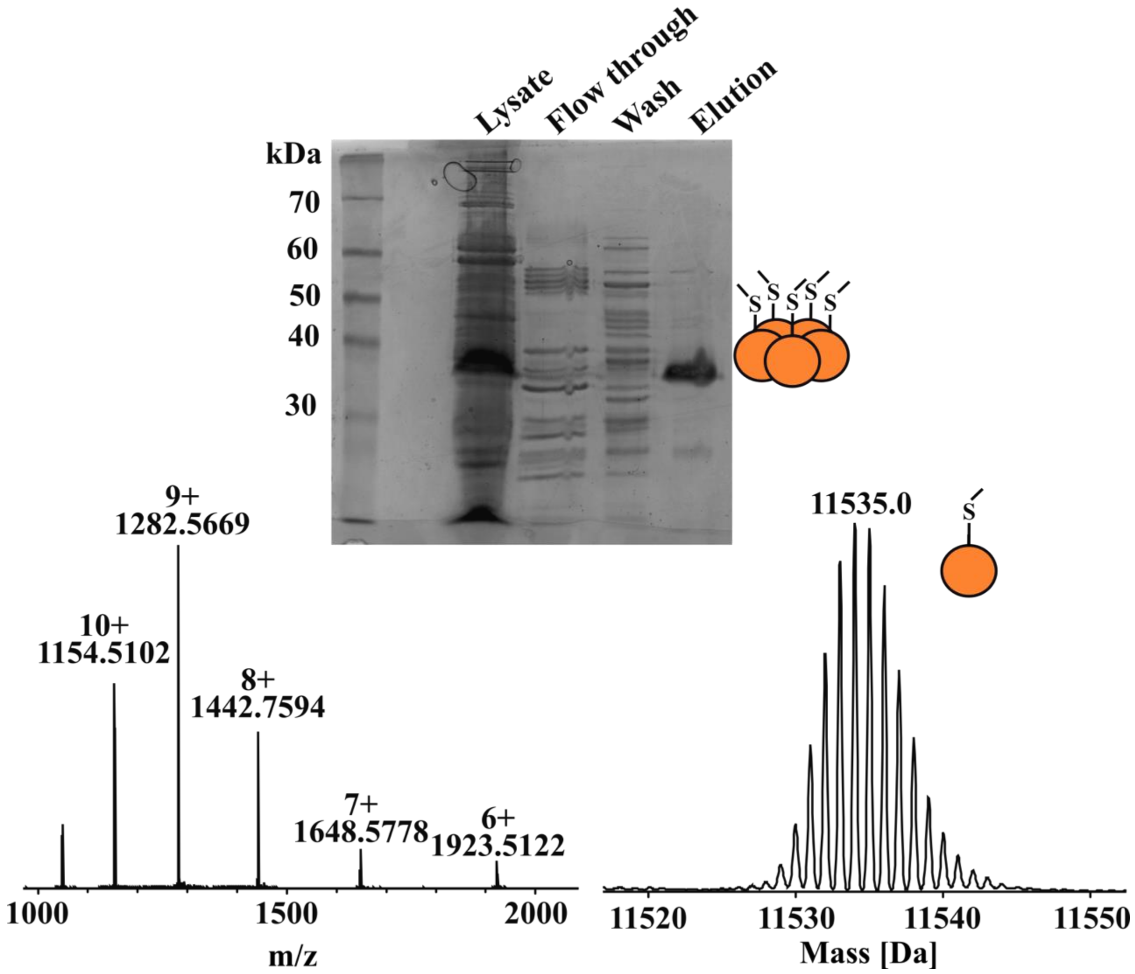

Figure S3. SDS-PAGE analysis from the purification of the non-binding Met-W88E mutant by Ni-NTA affinity chromatography. Charge state and deconvoluted mass spectra of the elution fraction are shown.

<u>Expression, purification, and characterization of CTB(M37L-M68L-M101L-K43Aha-W88E) aka N_3-W88E</u>

Methionine auxotrophic *E. coli* B834 (DE3) cells transformed with plasmid pSAB2.4 containing the gene for Met-W88E/N_3-W88E were used to inoculate 2 x 5 ml LB starter culture containing 100 µg/ml ampicillin (5 µl of 100 mg/ml stock), the starter cultures were incubated at 37 °C with shaking at 200 rpm overnight. The cells were harvested by centrifugation at 4500 x *g* for 10 minutes, and the supernatant was discarded. The pellet was then resuspended in new minimal media (NMM; Table S1) and 1 ml of the starter culture was used to inoculate 4 x 400 ml of NMM media containing 100 µg/ml

of ampicillin (400 μl of 100 mg/ml stock). The cultures were incubated at 37 °C until the OD_{600} indicated that cell growth had halted due to depletion of methionine in NMM, which was reached at around 3 hours. After adding azidohomoalanine (1 ml of a 35 mg/ml stock – final concentration ca. 0.24 mM), expression was induced by addition of IPTG (final concentration 0.5 mM), and the cultures were left at 30 °C overnight. The cells were harvested by centrifugation at 13,000 x *g* for 30 minutes. The cells were resuspended in PBS pH 7.2 (10 ml) and lysed by addition of 1 ml BugBuster (10x lysis reagent). The suspension was agitated on rocker at room temperature for 40 minutes. The cell fragments were pelleted by centrifugation at 13,000 x *g* for 10 minutes, and the pellet discarded. The supernatant was purified using Ni-NTA chromatography, washing in PBS pH 7.2 and eluting in PBS pH 7.2 containing 200 mM imidazole. The protein was further purified by gel filtration using a Superdex 75 16/60 in PBS.

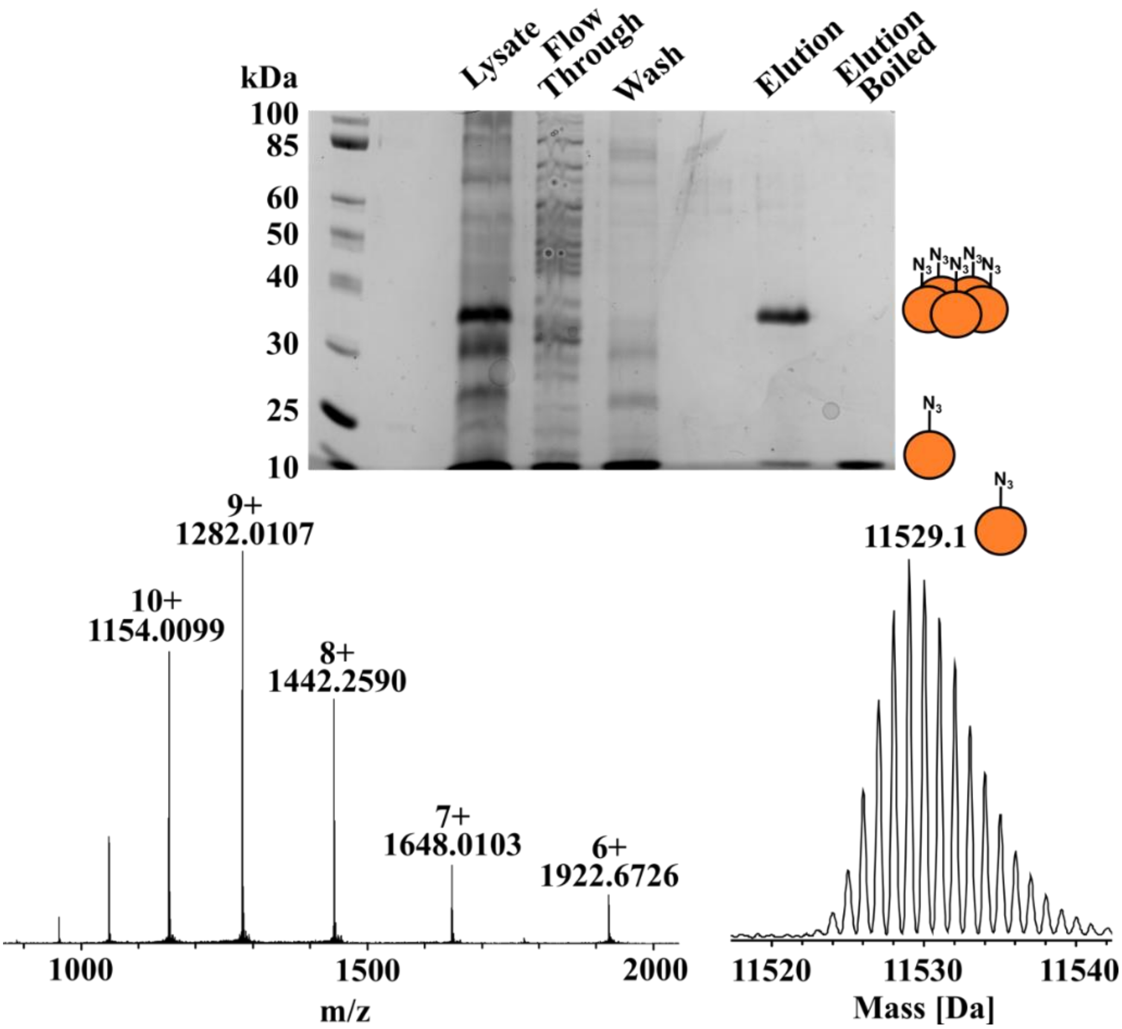

Figure S4. SDS-PAGE analysis of the purification of **N_3-W88E** using a Ni-NTA column. The elution fraction is shown both with the pentameric **N_3-W88E**, and the monomer after boiling. B) shows HRMS of the elution fraction from Ni-NTA column with the charge state and the deconvoluted spectra.

Following isolation of the **N_3-W88E** protein, the sample was compared to simulated isotopic distribution patterns to confirm the level of azidohomoalanine incorporation. The distribution was found to match the simulation for a 95:5 ratio of **N_3-W88E**:**Met-W88E** (Figure S8).

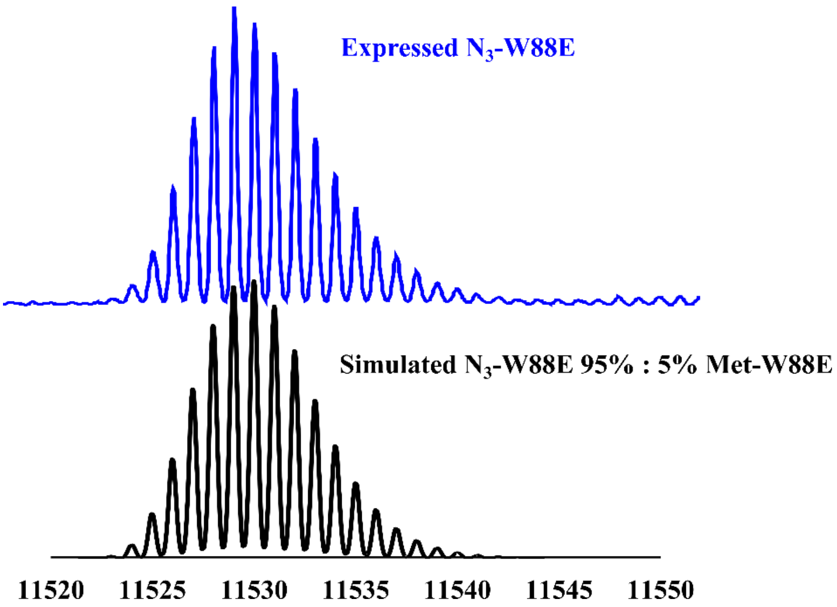

Figure S5. A comparison between the isotopic distribution of the expressed sample of **N_3-W88E** to a simulated isotope pattern for a protein consisting of 95:5 **N_3-W88E**:**Met-W88E**).

Following purification of the **Met-W88E** and **N_3-W88E** proteins, the two variants were compared to the wildtype (WT) CTB protein by SDS-PAGE and circular dichroism (CD), to assess their stability and confirm that the variants were correctly folded. SDS-PAGE of unboiled samples confirmed the presence of the pentameric protein in each case (Figure S5). The mutant proteins migrated faster through the gel than WT CTB despite having a similar mass; this can be explained by the change in the overall surface charge of the protein resulting from removal of five surface lysine residues. After boiling each sample, the pentamers dissociated into protomeric CTB with the expected mass. CD spectral analysis confirmed the folded structure of **N_3-W88E** was not significantly affected by the introduction of five mutations or azide incorporation. The small differences between the two CD spectra **N_3-W88E** and WT CTB can be accounted for by small discrepancies in protein concentration - **N_3-W88E**) contains no tryptophan residues, the extinction coefficient estimated by ProtParam (ExPASy) is unlikely to be as accurate as that for WT CTB.

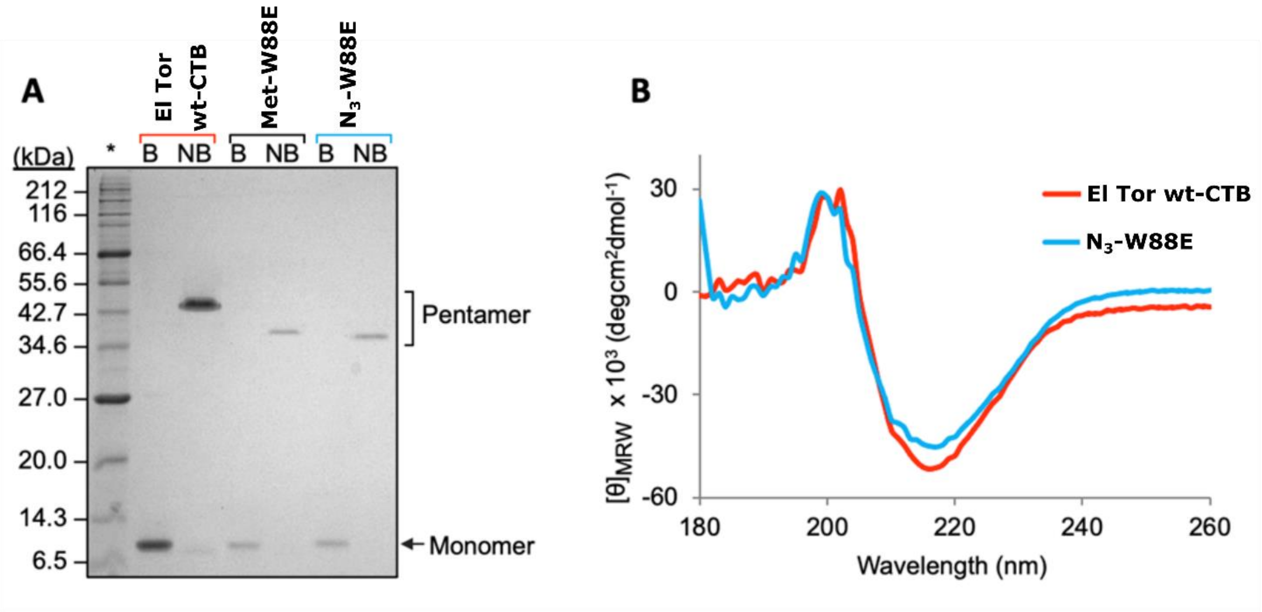

Figure S6. A) Comparison of the stability and migration rates of wild-type El Tor CTB and mutant CTB pentamers. All samples which were 'boiled' (B) (i.e. incubated for 10 min at 95 °C) ran as monomers, whereas 'non-boiled' samples (NB) ran as pentamers. The difference in the migration rate of the three CTB pentamers can be explained differences in their surface charge distribution. B) Comparison of the CD spectra for wild type CTB and **N_3-W88E**) suggests that the five mutations that were introduced have not affected the folded structure of the protein. CD measurements were performed on a Jasco J715 spectropolarimeter at 20 °C, over a wavelength range of 260-180 nm. The measurements were made using a cuvette of 1 mm path length, with a bandwidth of 2.5, a step of 1 nm, at 1 sec per step. Protein samples were prepared at 15 µM in PBS pH 8.0. Two scans were run for each sample, the PBS baseline was subtracted from each, and the data were averaged.

Expression and purification of EGCase II

A pET-28a plasmid purchased from Genscript containing the gene for EGCase II was transformed into BL21 (Gold) *E. coli* cells, and a single colony was used to inoculate 5 ml LB containing 50 µg/ml kanamycin. The culture was incubated at 37 °C with shaking at 200 rpm overnight, and a glycerol cell stock of the *E. coli* clone was then prepared. The glycerol stock was harbouring the plasmid for EGCase II was used to inoculate 2 x 100 ml TYP broth containing 50 µg/ml kanamycin. The culture was incubated at 37 °C with shaking at 200 rpm until saturation was reached, at which point the cultures were removed from the incubator and placed in ice for 10 minutes. The incubator was cooled to 20 °C, and protein expression was induced by addition of IPTG (final concentration 0.1 mM) and the culture was incubated at 20 °C with shaking at 200 rpm for 8 hours. The cells were then harvested by centrifugation at 17000 x *g*, and the cell pellet was stored at -20 °C. The pellet was then resuspended in 2 ml phosphate buffer (50 mM NaH_2PO_4, 500 mM NaCl, pH 7) and the cells were lysed by addition

of BugBuster. The cell debris was removed by centrifugation at 30,000 x *g*, and the supernatant was retained. The pellet was resuspended in the same buffer and centrifuged again to ensure that all soluble protein had been removed from the insoluble fraction. The supernatants were combined (totalling 5 ml) and flowed through a Ni-NTA column which had been equilibrated in the same buffer. The column was washed with phosphate buffer (50 mM NaH_2PO_4, 500 mM NaCl, pH 7) containing 20 mM imidazole (3 x CV) and the protein was eluted from the column using an elution gradient of imidazole concentration of 50, 100, 200, 300, 400 and 500 mM, taking 10 ml fractions at each concentration.

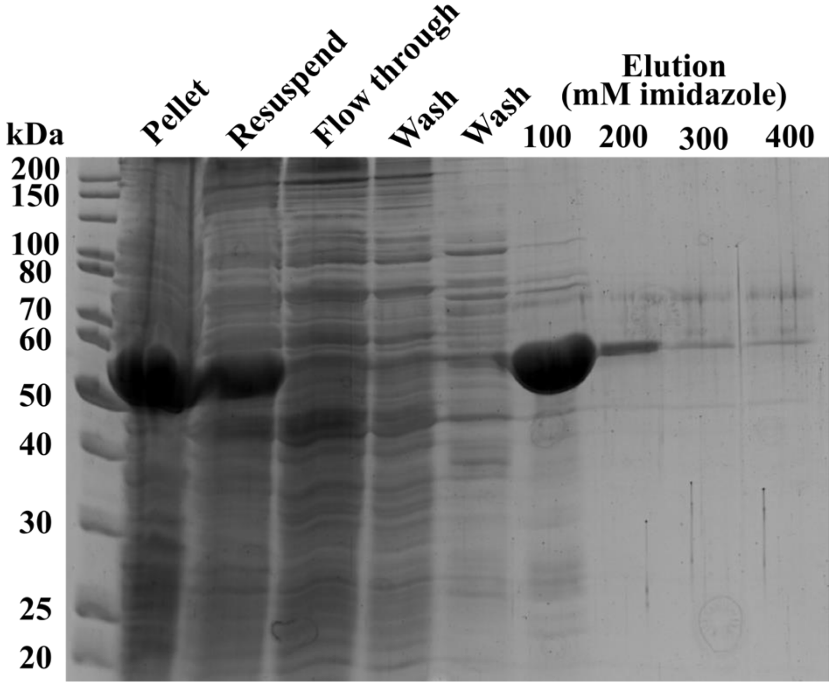

Figure S7. SDS-PAGE showing confirming expression of EGCase II, and purification by Ni-NTA column.

General Procedures for Protein Modification

Oxime Ligation with Linker 3

W88E (500 µL of a 384 µM stock) in sodium phosphate buffer pH 7.2, was combined with L-methionine (9.6 µL of a 200 mM stock, 10 eq) and $NaIO_4$ (4.8 µL of a 200 mM stock, 5 eq) (final concentrations: 3.84 mM L-methionine; 1.92 mM $NaIO_4$). **Note:** *It is important that the buffer does not contain potassium ions as reactions do not reach completion in PBS or other potassium containing buffers as a result of the lower solubility of potassium periodate*.[10] ESI-MS indicated that the reaction was complete after 10 minutes, yielding **oxidised-W88E**. The protein was purified using a PD-10 desalting column eluting in sodium phosphate buffer pH 6.8.

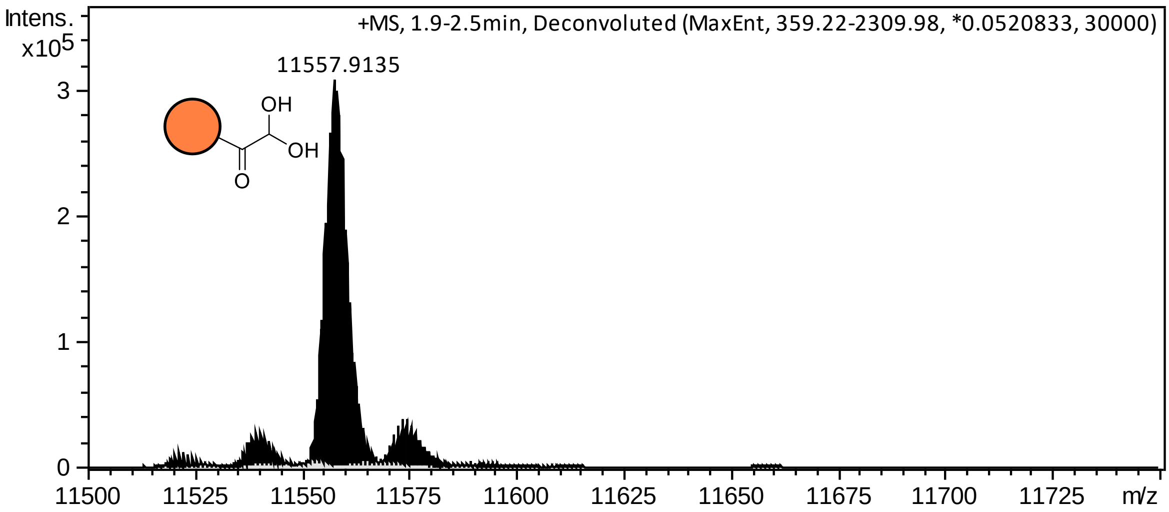

Figure S8. Deconvoluted mass spectrum of the W88E N-terminal oxidation reaction after 5 minutes (Theoretical mass **oxidised-W88E.hydrate** = 11558 Da).

Oxidised-W88E in sodium phosphate buffer pH 6.8, prepared in the previous step, was concentrated to ca. 450 µM using Amicon spin concentrator (MW = 10,000 Da cut off) and then used directly in the oxime ligation in the presence of aniline (1% *v/v*), and 4.5 mM BCN-oxyamine **3** (10 eq per protomer). The reaction was left agitating at 37 °C until ESI-MS showed the reaction was complete (typically 8-16 hours). The reaction mixture was then passed through a PD-10 desalting column, eluting the glycoprotein in sodium phosphate buffer pH 7.2.

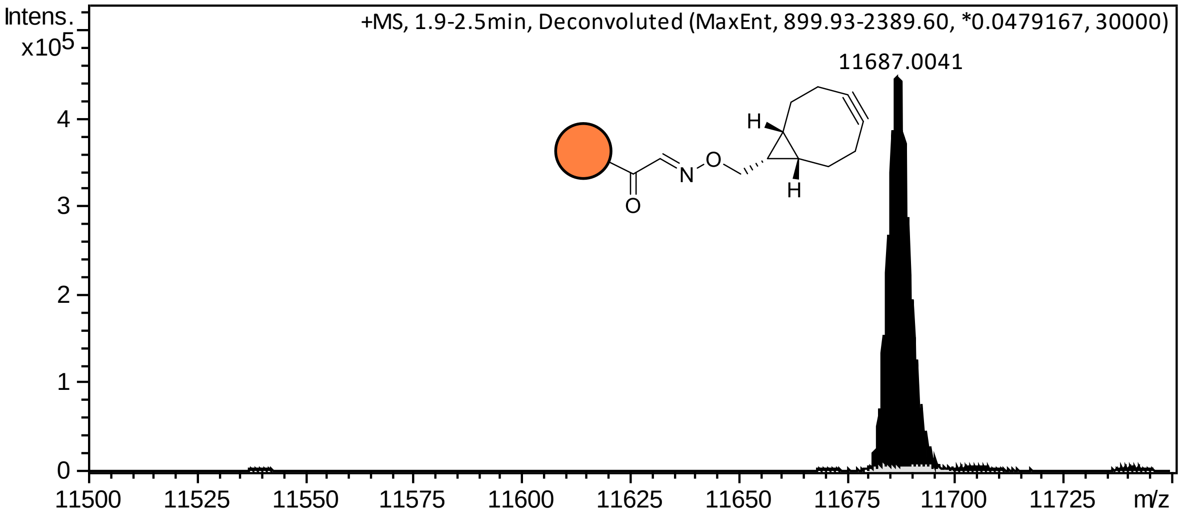

Figure S9. Deconvoluted mass spectrum showing the ligation of compound endo-**3** (4.5 mM) to **oxisised-W88E** (450 µM) to give **BCN-W88E** (Theoretical mass = 11687 Da).

__**SPAAC ligation to BCN-W88E**__

BCN-W88E in sodium phosphate buffer pH 7.2, prepared in the previous step, was concentrated to ca. 150 µM using Amicon spin concentrator (MW = 10,000 Da cut off). **BCN-W88E** (100 µL of a 150 µM

stock) was combined with glycosyl azide (7.5 µL of a 20 mM stock, 10 eq). The reaction was left agitating at 37 °C until ESMS showed the reaction was complete (typically 8-16 hours). The reaction mixture was then passed through a G-25 minitrap desalting column, eluting the glycoprotein in sodium phosphate buffer pH 7.2.

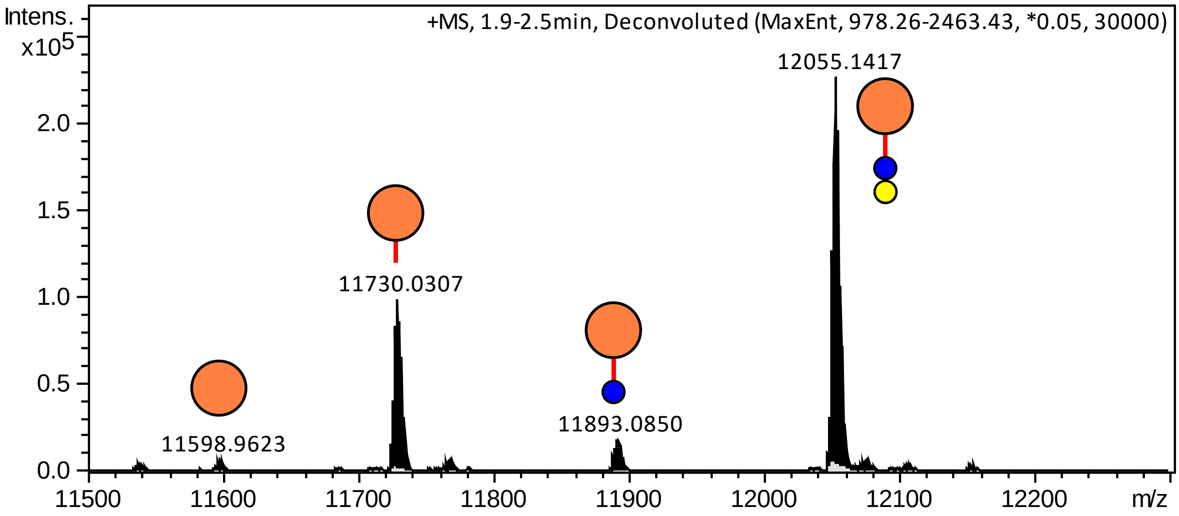

Figure S10. Deconvoluted mass spectrum showing the ligation of **BCN-W88E** (150 µM) to Lactosyl azide **9** (1.5 mM) to give **(Lac)BCN-W88E** (Theoretical mass = 12055 Da). Fragmentation within MS give rise to loss of terminal galactose (11893 Da) and lactose (11730 Da).

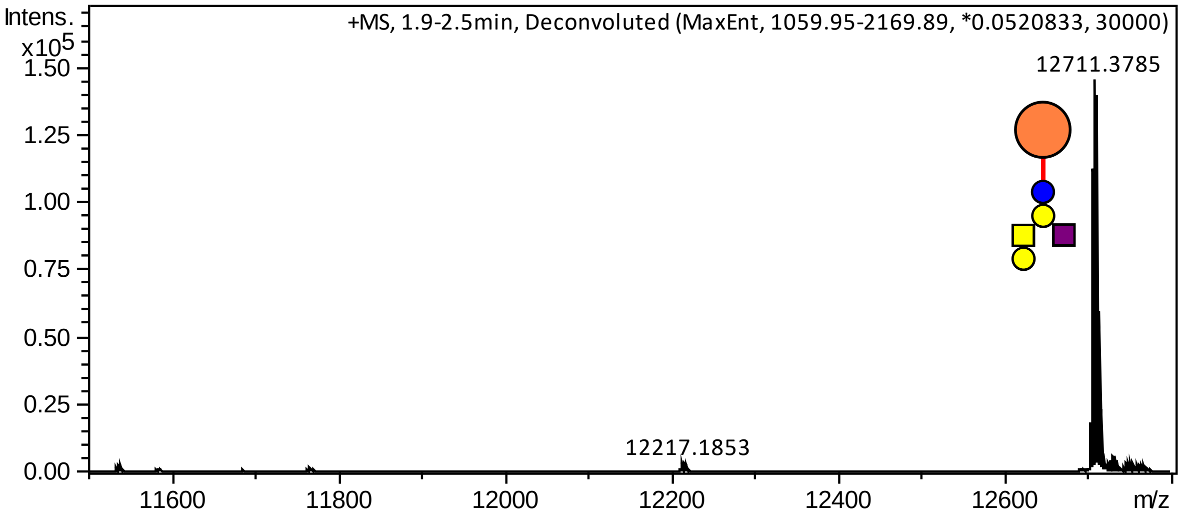

Figure S11. Deconvoluted mass spectrum showing the ligation of **BCN-W88E** (150 µM) to GM1 azide **13** (1.5 mM) to give **(GM1)BCN-W88E** (Theoretical mass = 12711 Da).

To verify additional signals observed in the HRMS of **(Lac)BCN-W88E** were caused by fragamentation within the MS and not due to NaN_3 contamination. **BCN-W88E** was reacted with 10eq of NaN_3 or 10 eq Lactosy azide **9**. No reaction or triazole formation was observed after 4 hrs for the reaction with

sodium azide confirming the appearance of peaks for **triazole-W88E** and **(Glc)BCN-W88E** is due to fragmentation of lactosyl azide moiety within the MS.

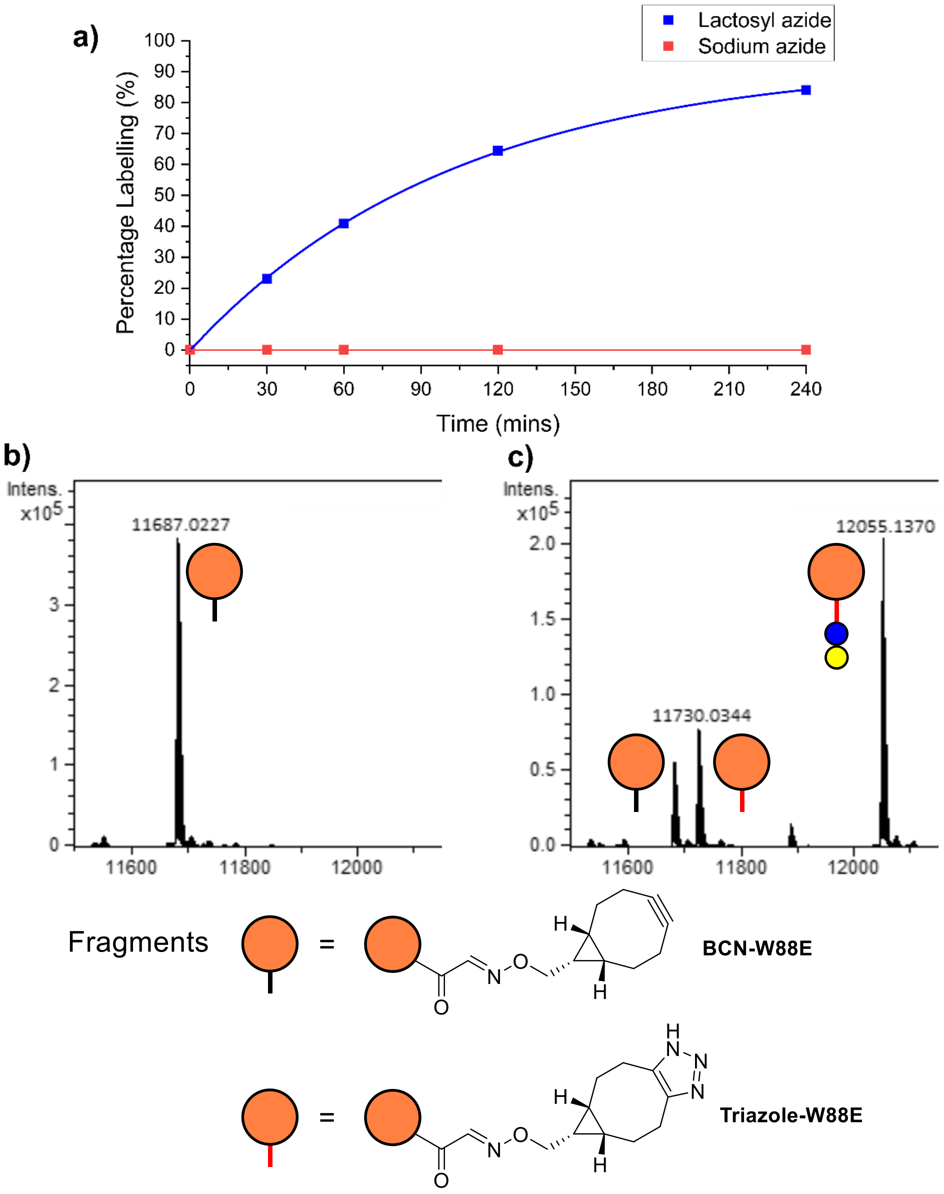

Figure S12. a) SPAAC labelling reaction progress over 4 hours for sodium azide and lactosyl azide (**4.8**). b) Deconvolved ES-MS of BCN-CTB treated with sodium azide (10 eq) for 4 hours. c) Deconvolved ES-MS of BCN-CTB treated with lactosyl azide (10 eq) for 4 hours.

<u>SPAAC ligation to N_3-W88E</u>

N_3-W88E (500 μM (protomeric concentration) in PBS pH 7.0 was combined with BCN glycan derivative compound **6/7** or **11** (1-20 eq in H_2O) and concentrated PBS (20 x). The reaction was incubated at 37 °C for between 4-8 hours, until ESMS showed the reaction was complete reaction. Once SPAAC conjugation was complete the reaction was purified using a G-25 desalting column, eluting the protein in PBS pH 7.2.

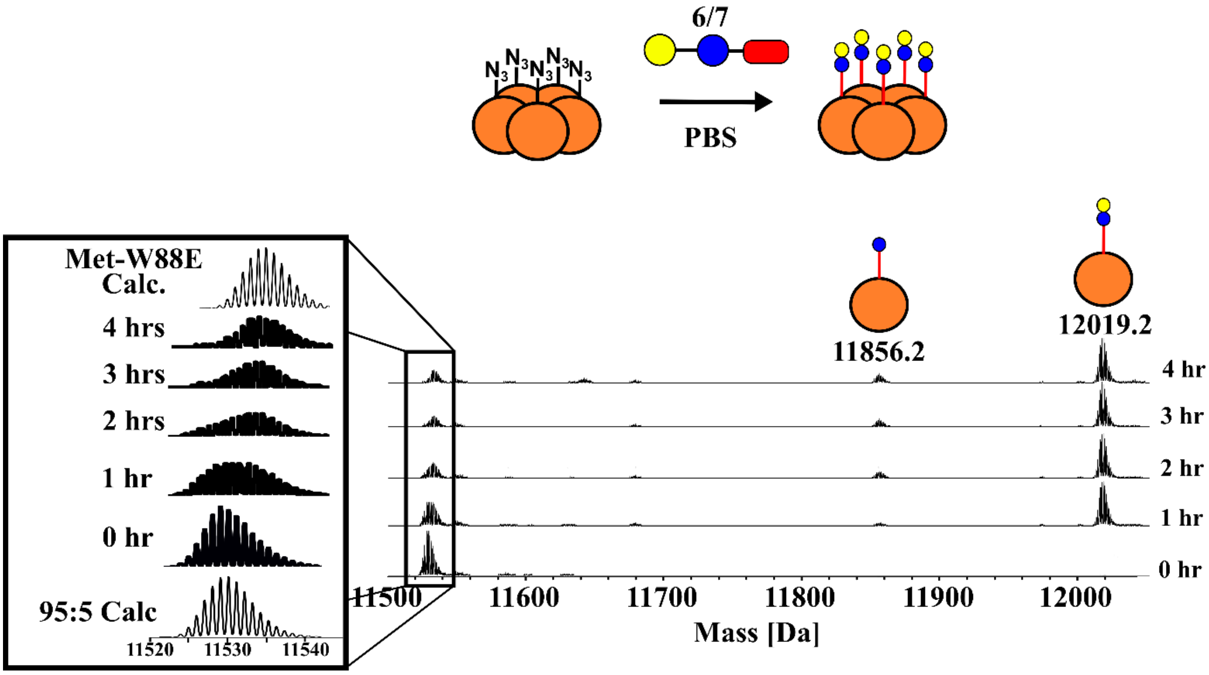

Figure S13. HRMS time course of SPAAC glycosylation of **N_3-W88E** (500 μM) with Lac-BCN (compound **6/7**, 10 mM) in PBS at 37 °C over seven hours. Stack plot of deconvoluted mass spectra show the conversion of **N_3-W88E** into the glycosylated protein. Fragmentation of the sugar is observed within the mass spectrometer. Simulated deconvoluted mass spectra ('calc') are also shown for **N_3-W88E** (95:5 **N_3-W88E**: **Met-W88E**) and unreactive **Met-W88E**.

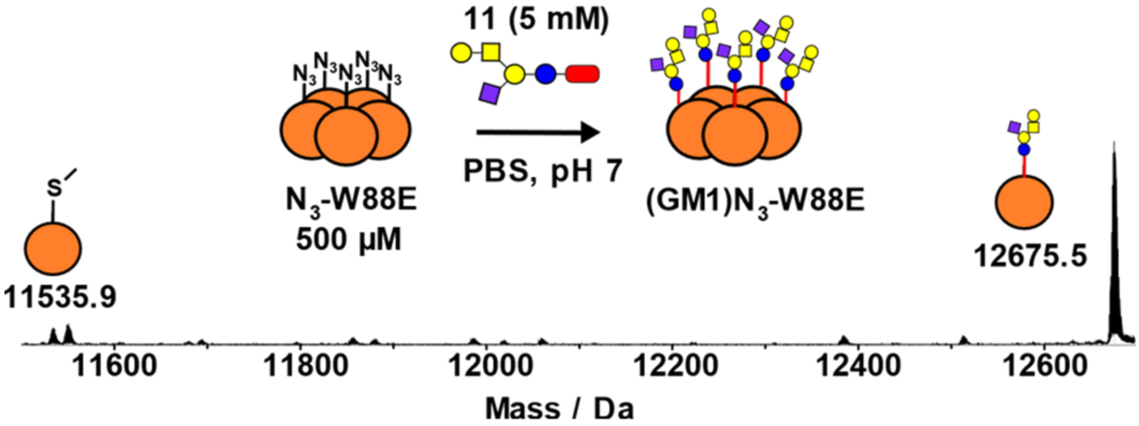

Figure S14. Deconvoluted HRMS of SPAAC glycosylation of **N_3-W88E** (500 μM) with GM1-BCN (compound **11**, 5 mM) in PBS at 37 °C over seven hours.

Following SPAAC ligation of both compounds **6/7** and **11** to **N_3-W88E**, the resulting neoglycoproteins were analysed by SDS-PAGE (figure S12).

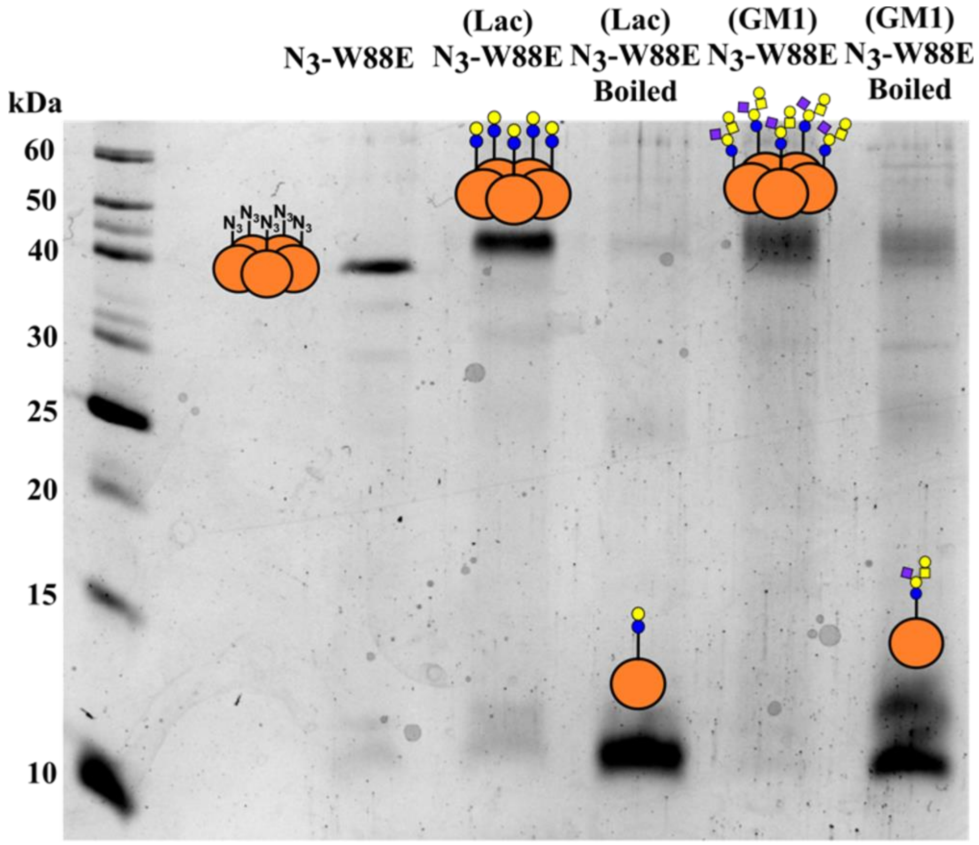

Figure S15. SDS-PAGE analysis comparing **N_3-W88E** with **(Lac)N_3-W88E** and **(GM1)N_3-W88E**. CTB non-boiled and boiled samples, showing bands around 40 kDa and 12 kDa, respectively. The folded structure of the pentamer has a tendency to migrate at a lower mass than expected for a pentamer as a result of its surface charge. Some hydrolysis of the GM1 oligosaccharides appears to have occurred upon boiling the samples giving partial conversion to a band that aligns with the (Lac)N3-W88E boiled lane.

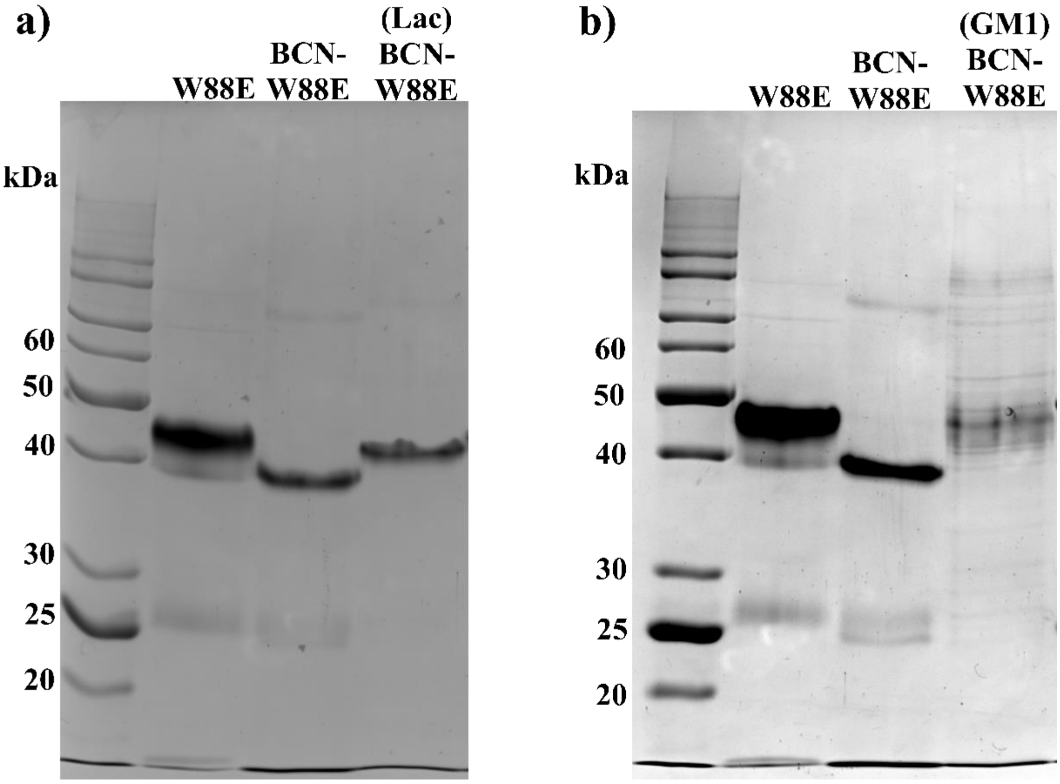

Figure S16. SDS-PAGE analysis comparing **W88E** & **BCN-W88E** with a) **(Lac)BCN-W88E** and b) **(GM1)BCN-W88E**. CTB samples not boiled prior to analysis. The folded structure of the pentamer has a tendency to migrate at a lower mass than expected for a pentamer as a result of its surface charge.

ELLA protocols

Lightning-Link® HRP conjugation to CTB

Using a 10-40 µg vial of the Lightning-Link® HRP conjugation kit, 10 µl of WT CTB (461 µM protomeric concentration, 1:1 pentamer:HRP) was added to the supplied vial and the solution was mixed to resuspend the lyophilised HRP. 1 µl of the LL-modifier reagent was added and the reaction was left for 3 hours at room temperature. The reaction was quenched with 1 µl of LL-quencher reagent and then quenching step was left for 30 minutes before use or storage. CTB-HRP was stored as 1 ml aliquots at 90 ng/ml in PBS which were flash frozen and stored at -20 °C.

96-well flat bottomed, high binding, polystyrene microtiter plates (Grenier 655077) were coated with ganglioside GM1 (100 µl, 1.3 M in methanol) and the solvent was allowed to evaporate at room temperature. The plate was washed with PBS (3 x 200 µl) to remove any remaining GM1, and any remaining binding sites on the well were blocked with BSA by incubating with a PBS solution containing 1% (w/v) bovine serum albumin (BSA, 100 µl) for 30 minutes at 37 °C. The wells were then washed again with PBS (3 x 200 µl). Inhibitor samples were prepared performing a 2-fold serial dilution in PBS

containing 0.1% (w/v) BSA, 0.05% (v/v) Tween-20 (PBS-T) in V-bottomed propylene 96-well plates (Grenier 651201) using a program developed on the Hamilton microlab STAR liquid handling system analysing each sample in triplicate. The samples were mixed with CTB-HRP in the same buffer to give a final concentration of 0.5 ng/ml. The mixture of inhibitor and toxin were incubated for 2 hours at room temperature before 100 µl was transferred to the GM1 coated well by the Hamilton microlab STAR liquid handling system. The limits of detection were analysed by control samples containing just CTB-HRP and no inhibitor, and a sample containing just buffer, used to obtain the maximum and minimum optical density values for CTB-HRP binding to the GM1 coated wells. The inhibitor toxin mixture was incubated for 30 minutes at room temperature before the plate was washed with PBS-T (3 x 200 µl) to remove any unbound CTB-HRP- inhibitor complex. After washing the plate, a solution of Amplex red® (100 µl, 5 µM Amplex Red®, 5 µM H_2O_2 in PBS) was added and fluorescence was measured at 25 °C for 30 minutes in a Perkin Elmer EnVision plate reader (excitation 531 nM; emission 595 nm). Initial rates were calculated if the data plots deviated from linearity.

Data processing

All samples were analysed in triplicate. The error of each sample was calculated as standard error (equation 1) where n equals the samples size, x is the observed initial rate value for each sample and x̃ is the mean rate value for each sample.

$$standard\ error = \frac{\sqrt{\frac{\sum(x-\bar{x})^2}{(n-1)}}}{\sqrt{n}}$$

Equation 1: Standard error in initial rate obtained for each sample.

The fluorescence was then converted to percentage binding by comparison with the maximum and minimum values obtained from the positive and negative controls and errors were propagated accordingly. This data was then plotted against log (inhibitor concentration) for each sample in origin and the curve fitting was performed using the non-linear curve fit, using the Origin logistic function (equation 2), where A_1 is the curve's maximum, A_2 is the curve's minimum, x_0 is equal to the IC_{50}, x is the log (inhibitor concentration), and p is the Hill slope parameter.

$$y = \frac{A_1 - A_2}{1 + (x/x_0)^p} + A_2$$

Equation 2: Equation for logistic curve fitting.

References

(1) Dommerholt, J.; Schmidt, S.; Temming, R.; Hendriks, L. J. A.; Rutjes, F. P. J. T.; van Hest,

J. C. M.; Lefeber, D. J.; Friedl, P.; van Delft, F. L. Readily Accessible Bicyclononynes for Bioorthogonal Labeling and Three-Dimensional Imaging of Living Cells. *Angew. Chemie Int. Ed.* **2010**, *49* (49), 9422–9425.
(2) Ong, R. L.; Yu, R. K. 1H-NMR Assignments of GM1-Oligosaccharide in Deuterated Water at 500 MHz by Two-Dimensional Spin-Echo J-Correlated Spectroscopy. *Arch. Biochem. Biophys.* **1986**, *245* (1), 157–166.
(3) Sabesan, S.; Bock, K.; Lemieux, R. U. The Conformational Properties of the Gangliosides G M2 and G M1 Based on 1 H and, 13 C Nuclear Magnetic Resonance Studies. *Can. J. Chem.* **1984**, *62* (6), 1034–1045.
(4) Kartha, K. P. R.; Jennings, H. J. A Simplified, One-Pot Preparation of Acetobromosugars from Reducing Sugars. *J. Carbohydr. Chem.* **1990**, *9* (5), 777–781.
(5) Seifried, B. M.; Qi, W.; Yang, Y. J.; Mai, D. J.; Puryear, W. B.; Runstadler, J. A.; Chen, G.; Olsen, B. D. Glycoprotein Mimics with Tunable Functionalization through Global Amino Acid Substitution and Copper Click Chemistry. *Bioconjug. Chem.* **2020**, *31* (3), 554–566.
(6) Lau, Y.; Spring, D. Efficient Synthesis of Fmoc-Protected Azido Amino Acids. *Synlett* **2011**, *2011* (13), 1917–1919.
(7) Wiltschi, B. Expressed Protein Modifications: Making Synthetic Proteins. In *Synthetic Gene Networks: Methods and Protocols*; Weber, W., Fussenegger, M., Eds.; Humana Press: Totowa, NJ, 2012; pp 211–225.
(8) Branson, T. R.; McAllister, T. E.; Garcia-Hartjes, J.; Fascione, M. A.; Ross, J. F.; Warriner, S. L.; Wennekes, T.; Zuilhof, H.; Turnbull, W. B. A Protein-Based Pentavalent Inhibitor of the Cholera Toxin B-Subunit. *Angew. Chemie Int. Ed.* **2014**, *53* (32), 8323–8327.
(9) Haigh, J. L.; Williamson, D. J.; Poole, E.; Guo, Y.; Zhou, D.; Webb, M. E.; Deuchars, S. A.; Deuchars, J.; Turnbull, W. B. A Versatile Cholera Toxin Conjugate for Neuronal Targeting and Tracing. *Chem. Commun.* **2020**, *56* (45), 6098–6101.
(10) Brabham, R. L.; Keenan, T.; Husken, A.; Bilsborrow, J.; McBerney, R.; Kumar, V.; Turnbull, W. B.; Fascione, M. A. Rapid Sodium Periodate Cleavage of an Unnatural Amino Acid Enables Unmasking of a Highly Reactive α-Oxo Aldehyde for Protein Bioconjugation. *Org. Biomol. Chem.* **2020**, *18* (21), 4000–4003.

NMR & MS Spectra

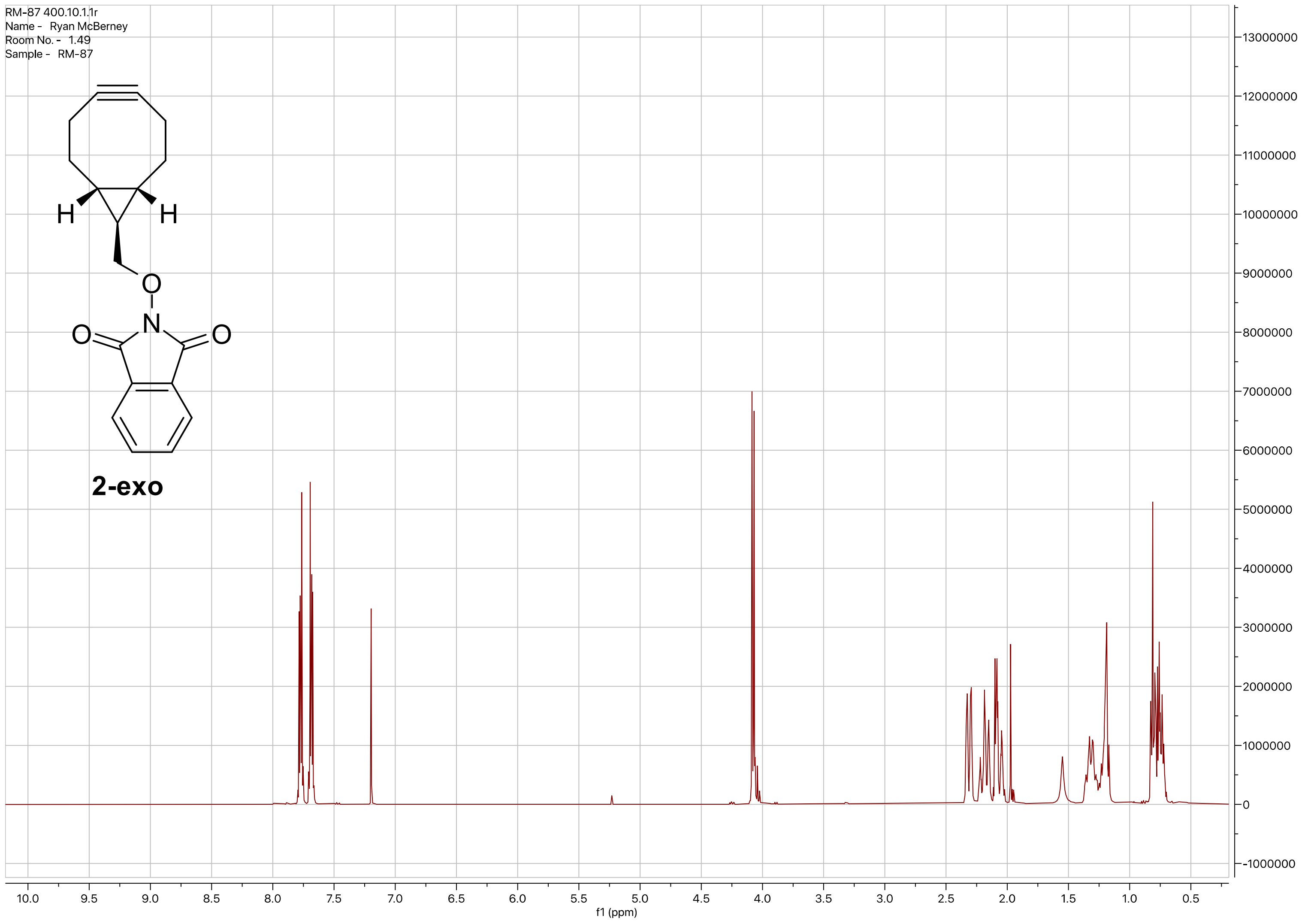

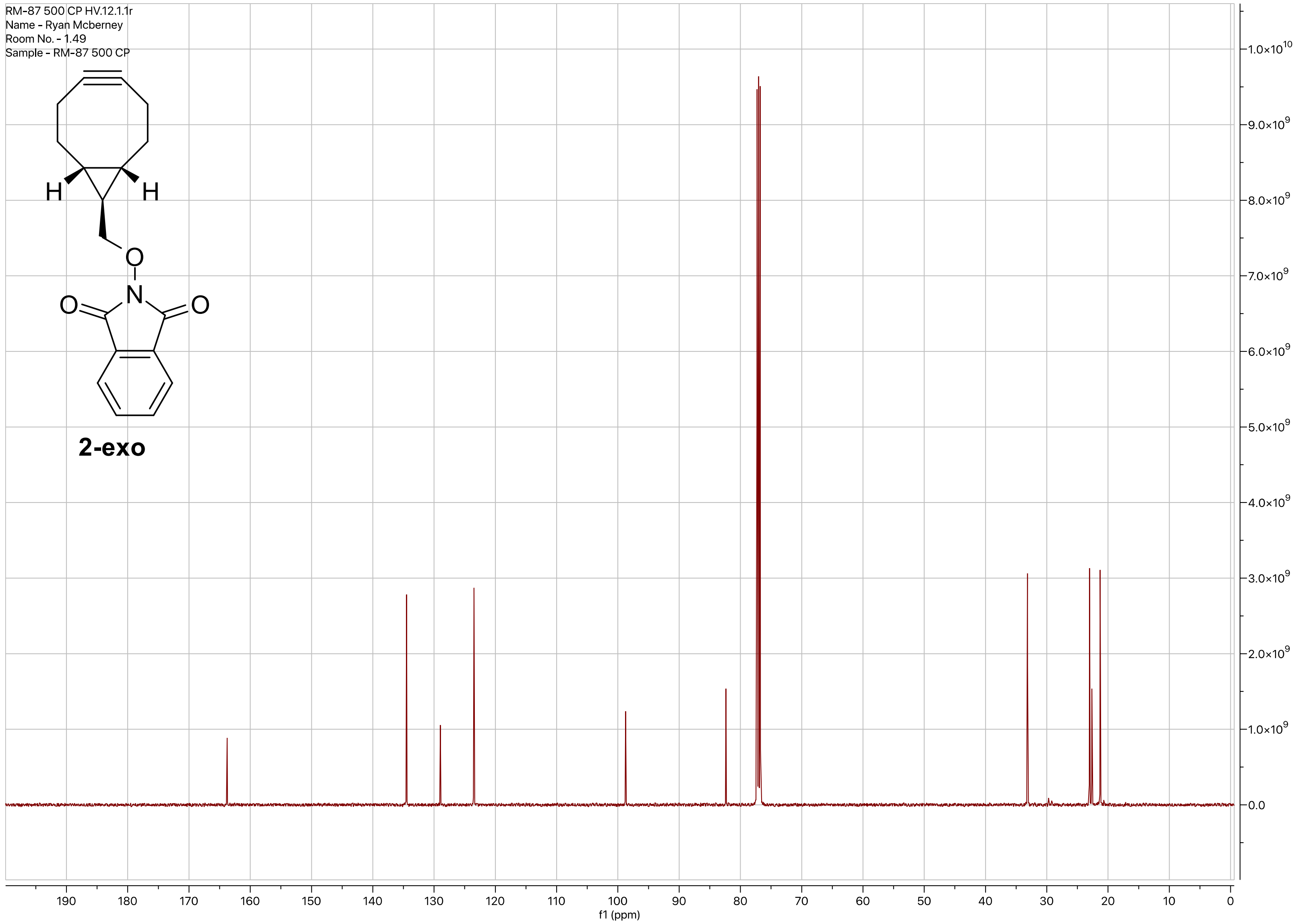
RM-87 500 CP HV.12.1.1r
Name - Ryan Mcberney
Room No. - 1.49
Sample - RM-87 500 CP
H
H
O
N
O
O
2-exo
1.0×10^10
9.0×10^9
8.0×10^9
7.0×10^9
6.0×10^9
5.0×10^9
4.0×10^9
3.0×10^9
2.0×10^9
1.0×10^9
0.0
190
180
170
160
150
140
130
120
110
100
90
80
70
60
50
40
30
20
10
0
f1 (ppm)

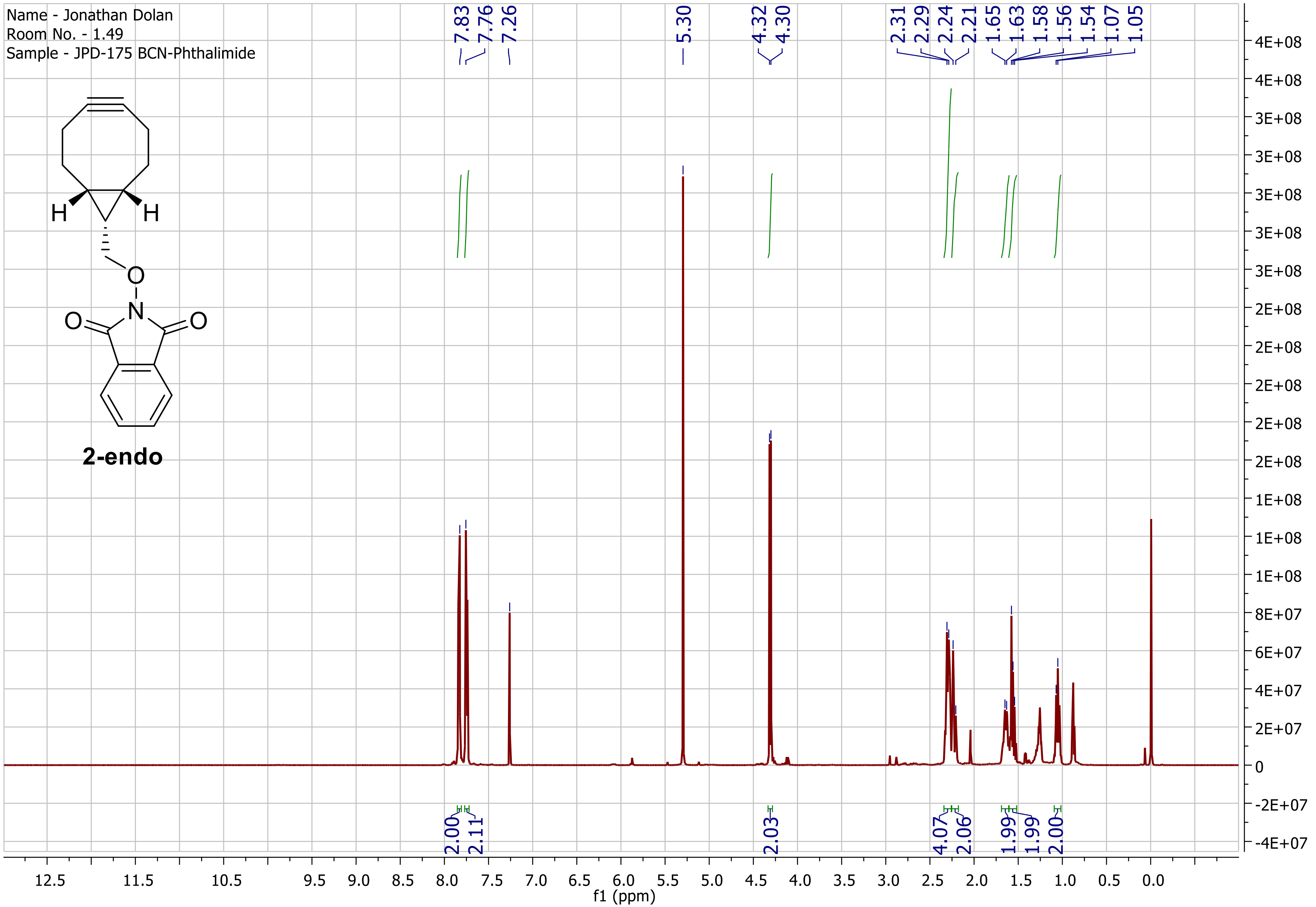

Name - Jonathan Dolan
Room No. - 1.49
Sample - JPD-175 BCN-Phthalimide
2-endo
7.83 7.76 7.26
5.30
4.32 4.30
2.31 2.29 2.24 2.21 1.65 1.63 1.58 1.56 1.54 1.07 1.05
2.00 2.11 2.03 4.07 2.06 1.99 1.99 2.00
f1 (ppm)

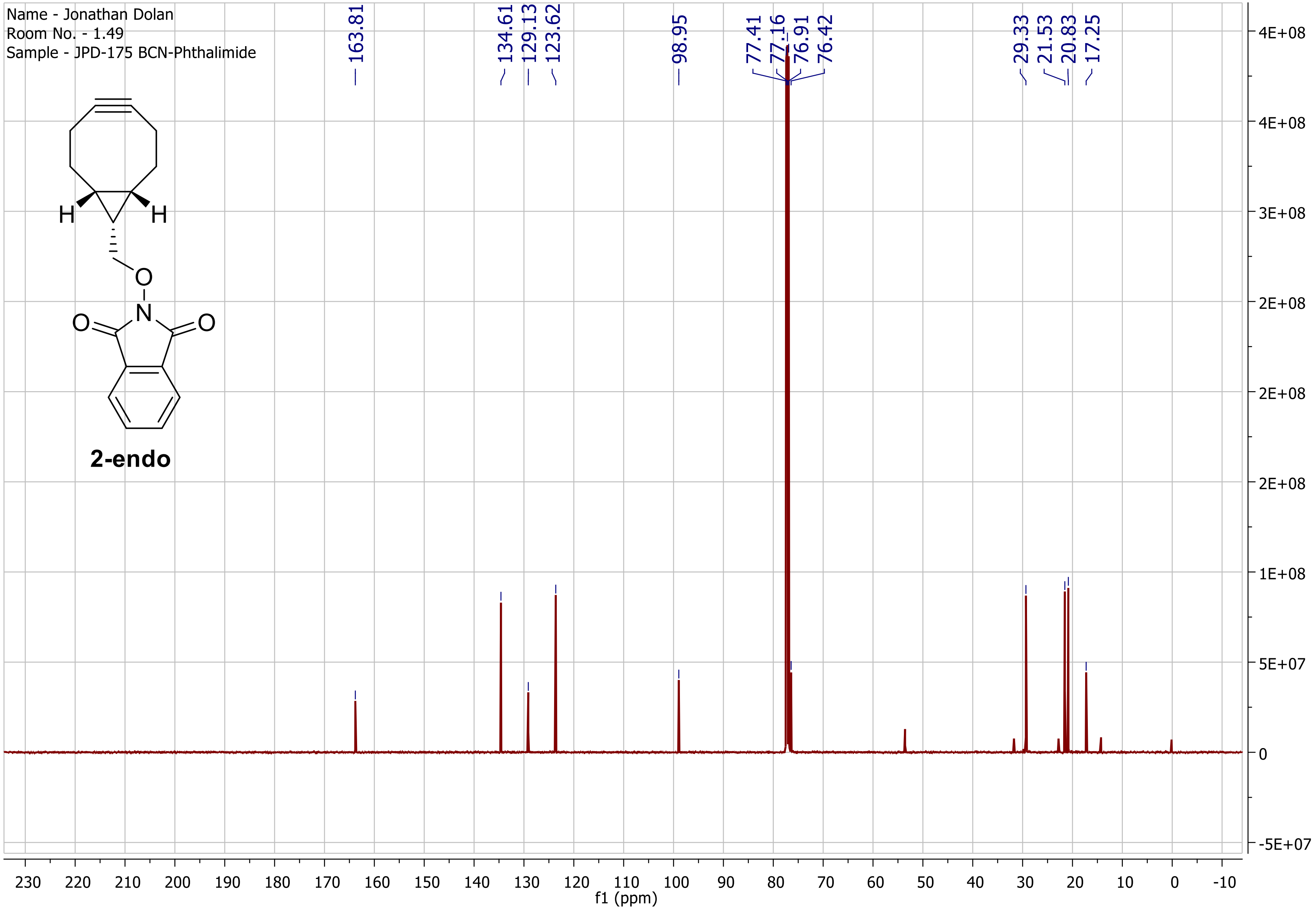
Name - Jonathan Dolan
Room No. - 1.49
Sample - JPD-175 BCN-Phthalimide
163.81
134.61
129.13
123.62
98.95
77.41
77.16
76.91
76.42
29.33
21.53
20.83
17.25
2-endo
f1 (ppm)

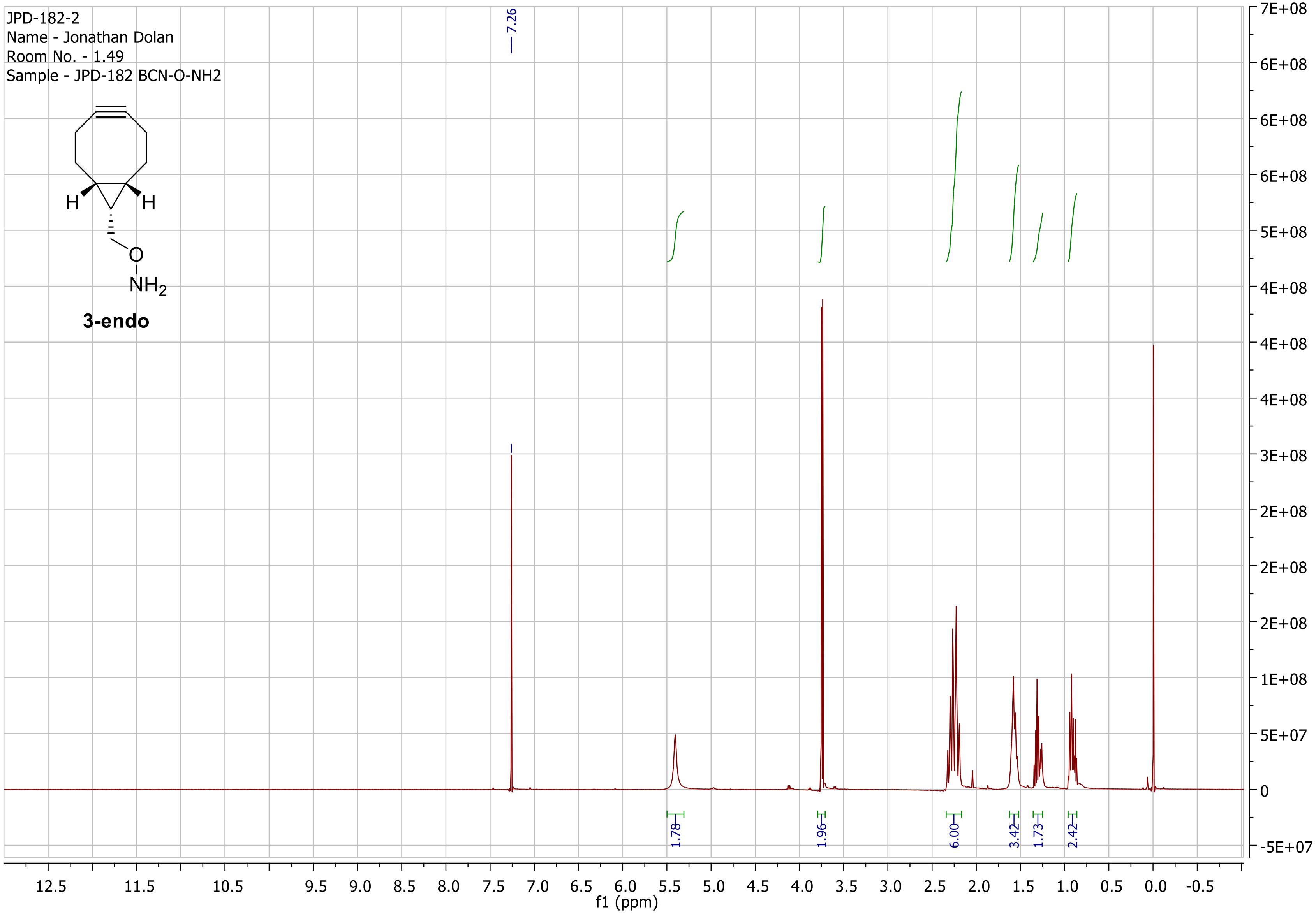

JPD-182-2
Name - Jonathan Dolan
Room No. - 1.49
Sample - JPD-182 BCN-O-NH2
3-endo
7.26
1.78
1.96
6.00
3.42
1.73
2.42
f1 (ppm)

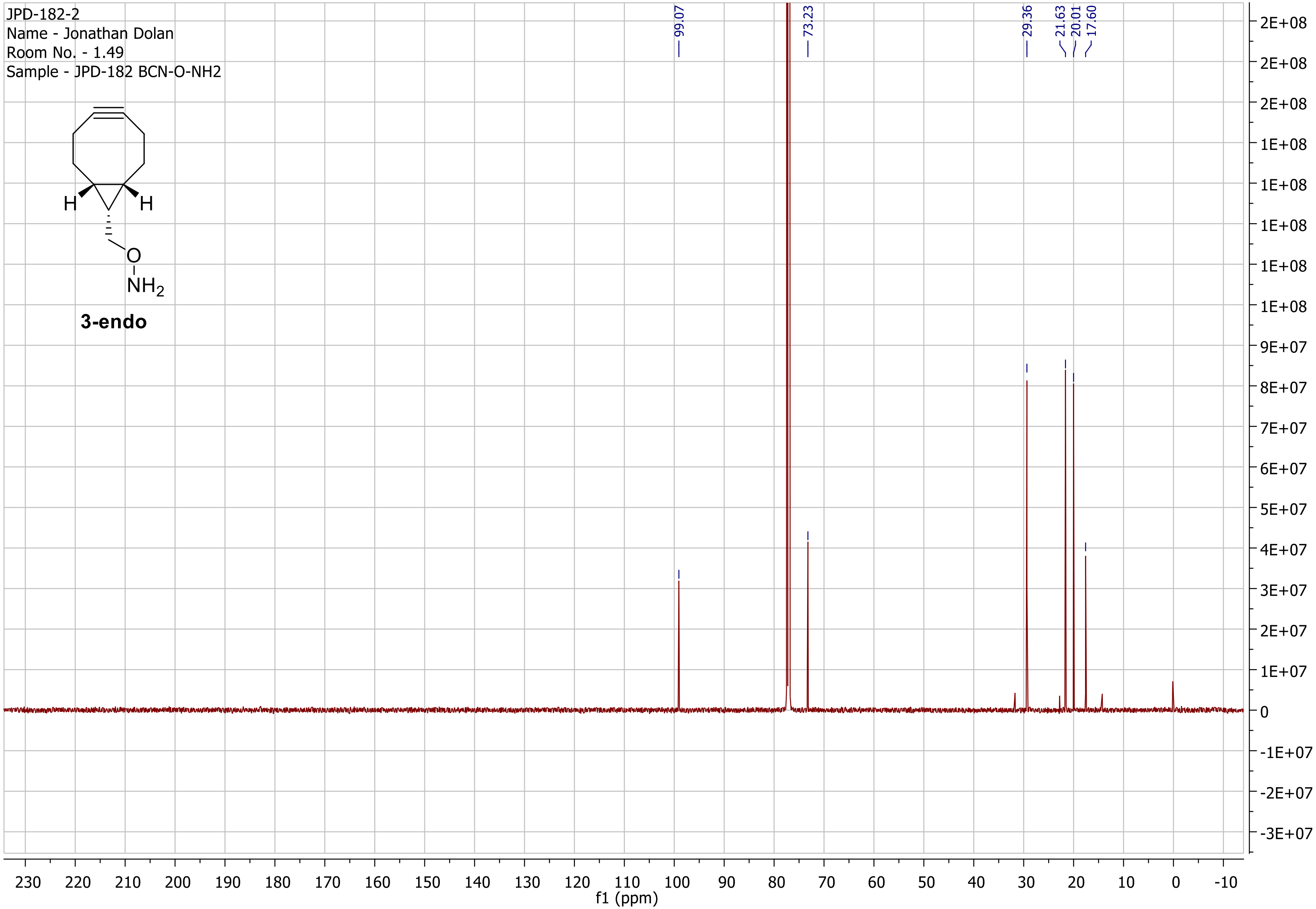
JPD-182-2
Name - Jonathan Dolan
Room No. - 1.49
Sample - JPD-182 BCN-O-NH2
3-endo
99.07
73.23
29.36
21.63
20.01
17.60
f1 (ppm)

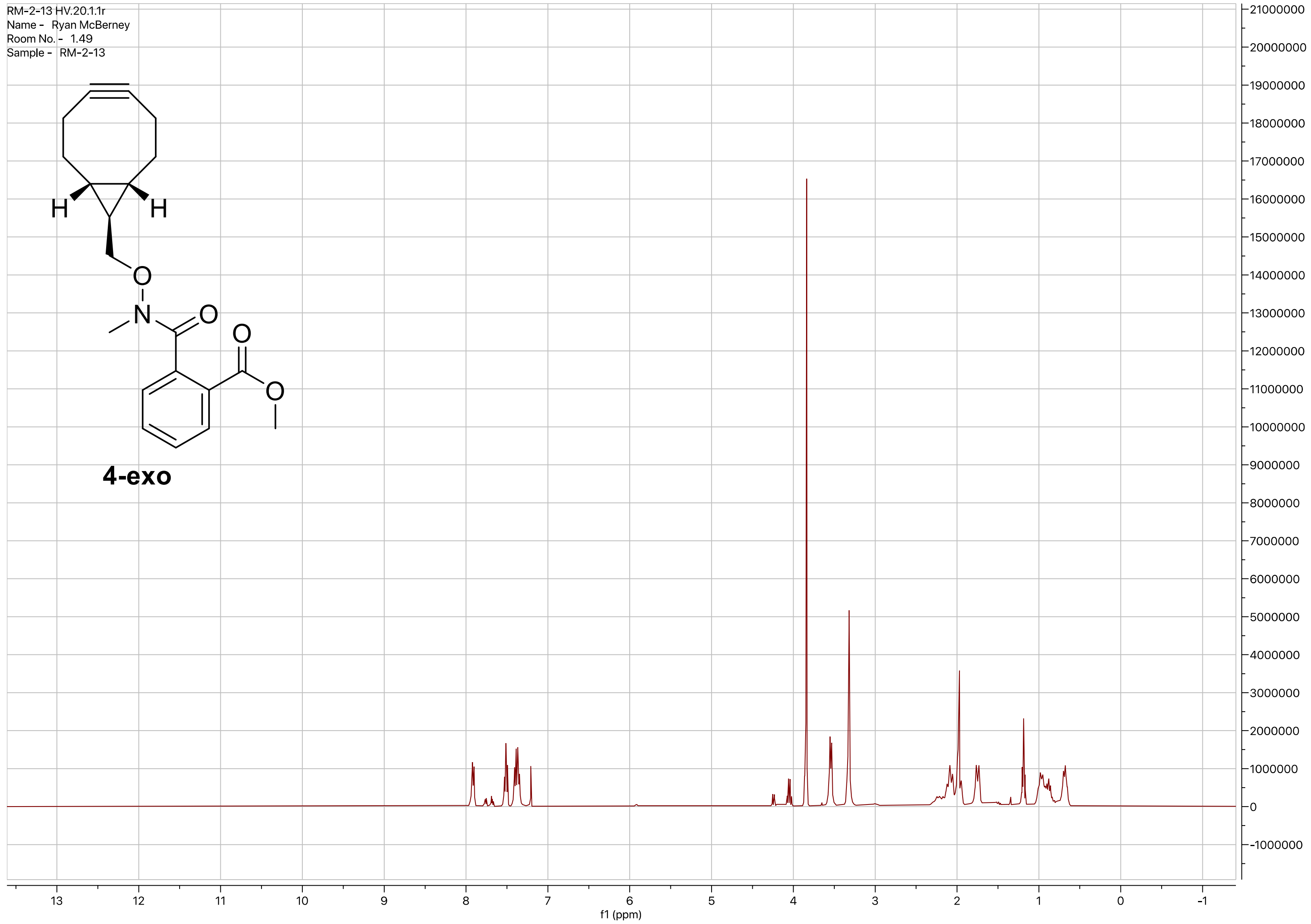
RM-2-13 HV.20.1.1r
Name - Ryan McBerney
Room No. - 1.49
Sample - RM-2-13
4-exo
f1 (ppm)

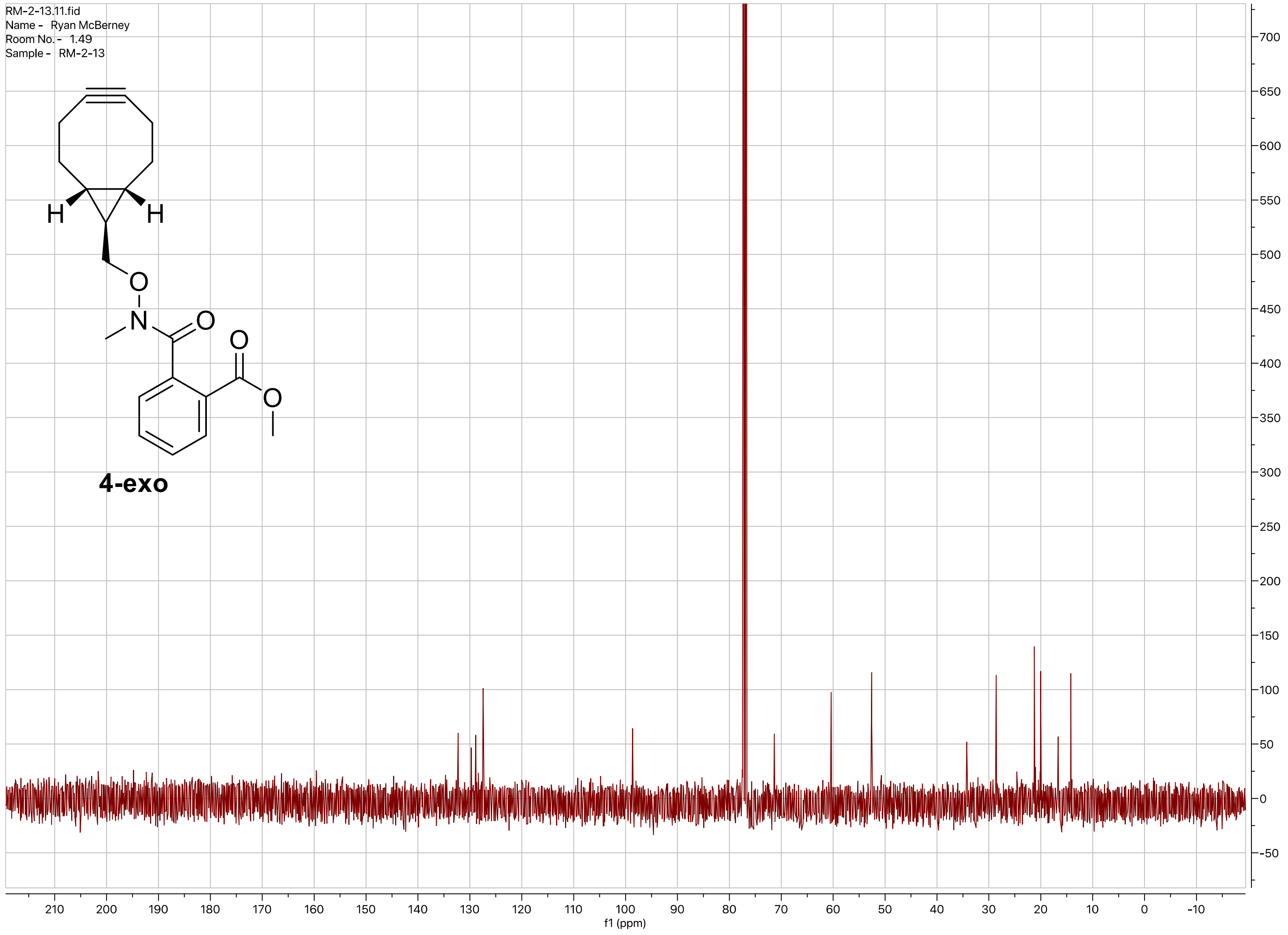

RM-2-13.11.fid
Name - Ryan McBerney
Room No. - 1.49
Sample - RM-2-13
4-exo
f1 (ppm)

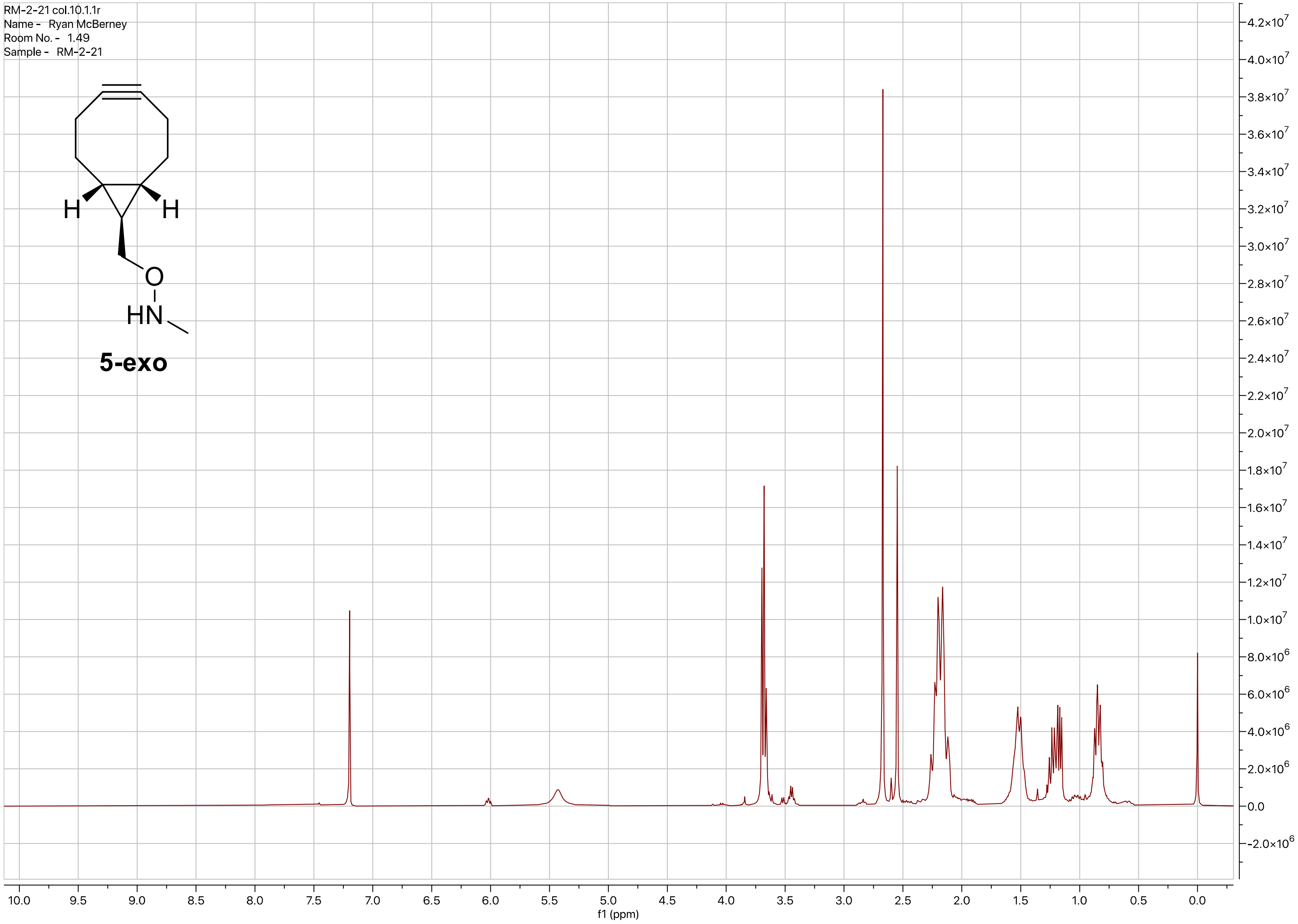
RM-2-21 col.10.1.1r
Name - Ryan McBerney
Room No. - 1.49
Sample - RM-2-21
H
H
O
HN
5-exo
f1 (ppm)

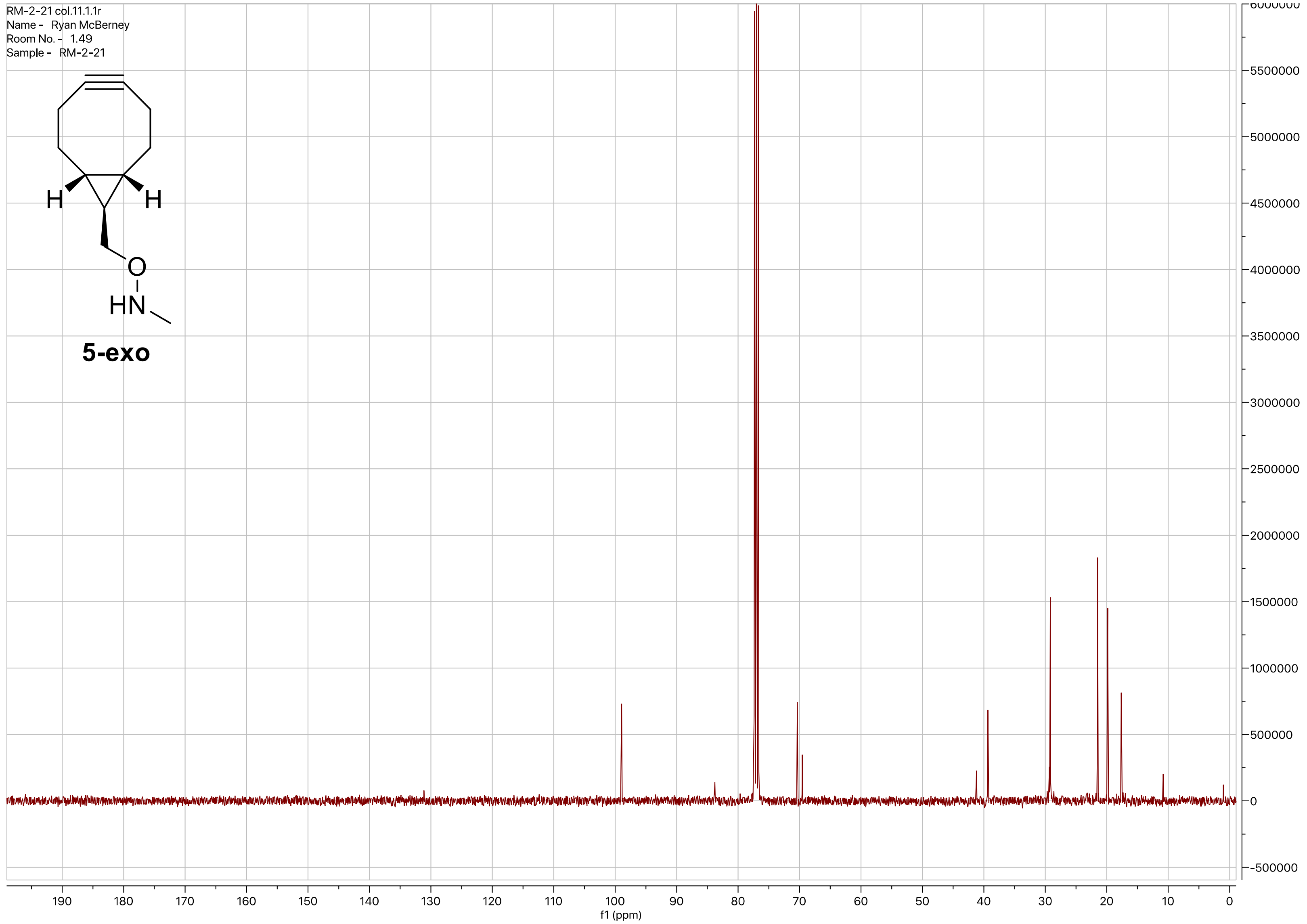
RM-2-21 col.11.1.1r
Name - Ryan McBerney
Room No. - 1.49
Sample - RM-2-21
5-exo
f1 (ppm)

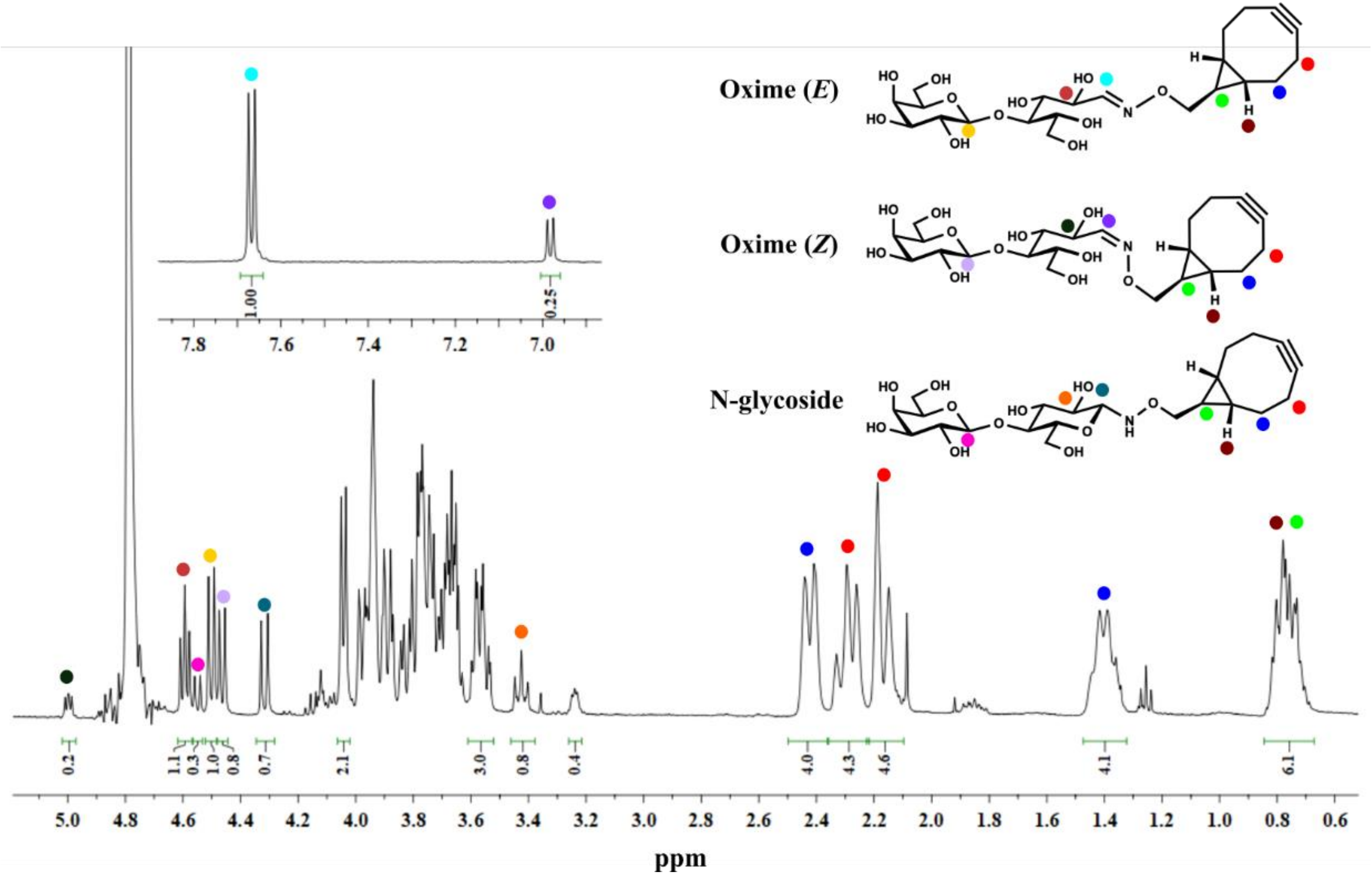
Oxime (*E*)
Oxime (*Z*)
N-glycoside
ppm

HRMS for compound **8** (LacNMeBCN)

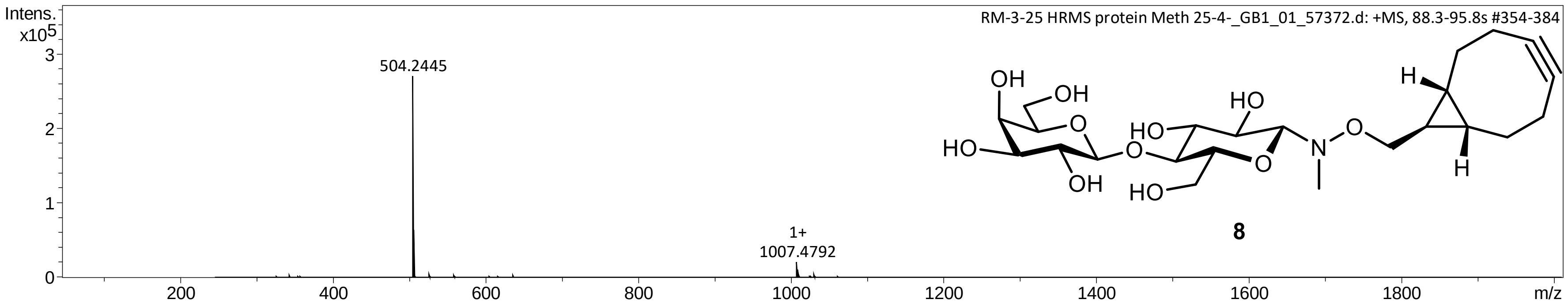

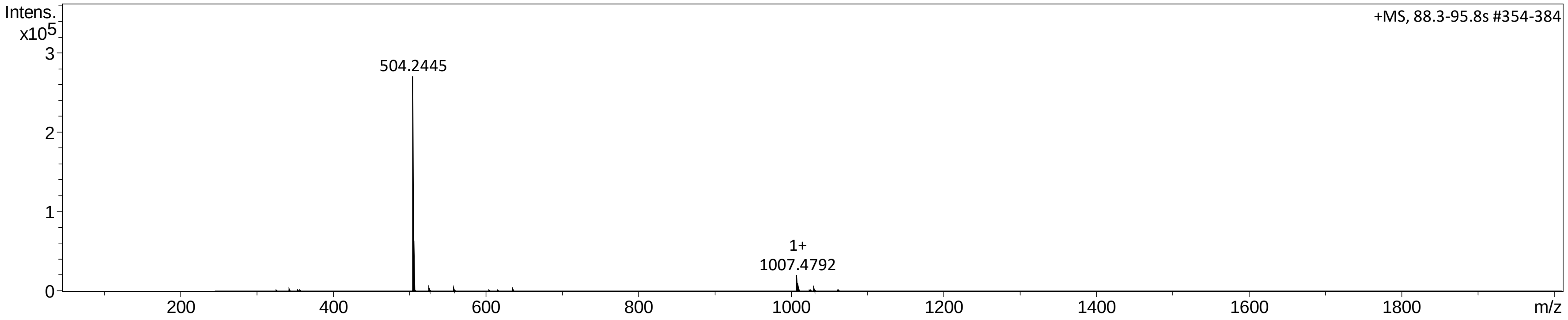

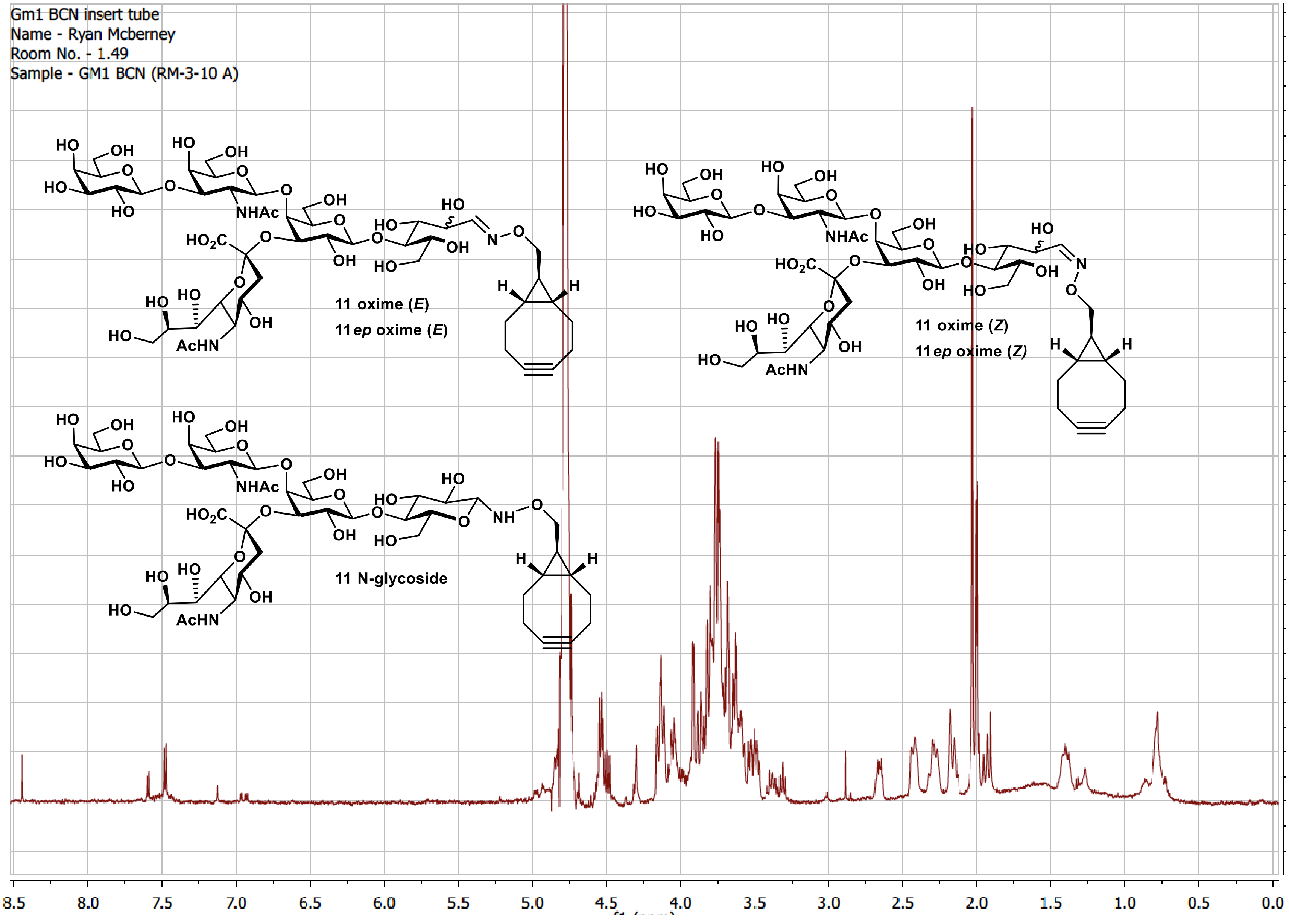

Gm1 BCN insert tube
Name - Ryan Mcberney
Room No. - 1.49
Sample - GM1 BCN (RM-3-10 A)
11 oxime (E)
11 ep oxime (E)
11 oxime (Z)
11 ep oxime (Z)
11 N-glycoside
8.5
8.0
7.5
7.0
6.5
6.0
5.5
5.0
4.5
4.0
3.5
3.0
2.5
2.0
1.5
1.0
0.5
0.0

HRMS for compound **11** (GM1-BCN)

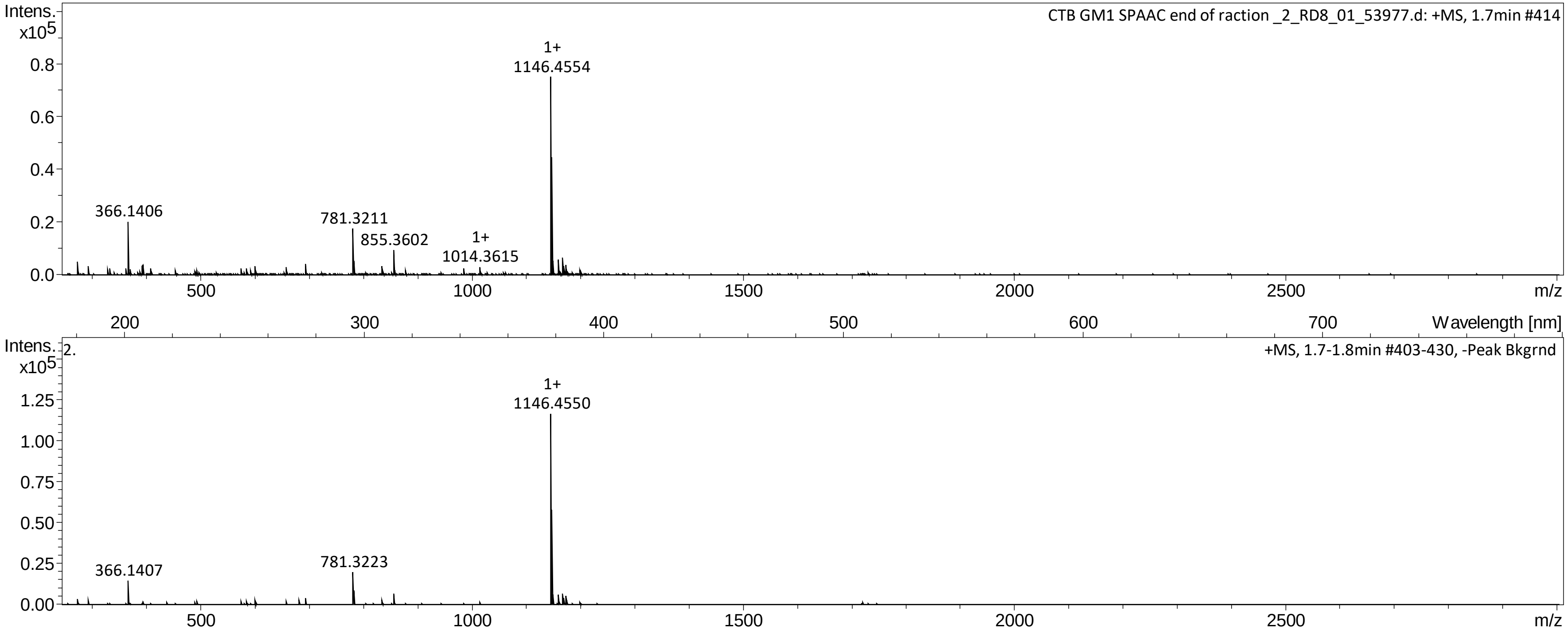

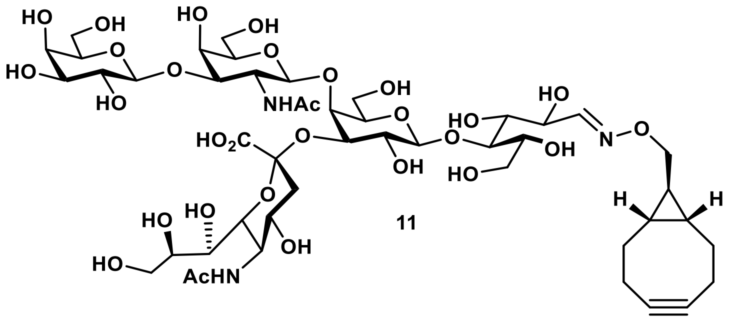

11

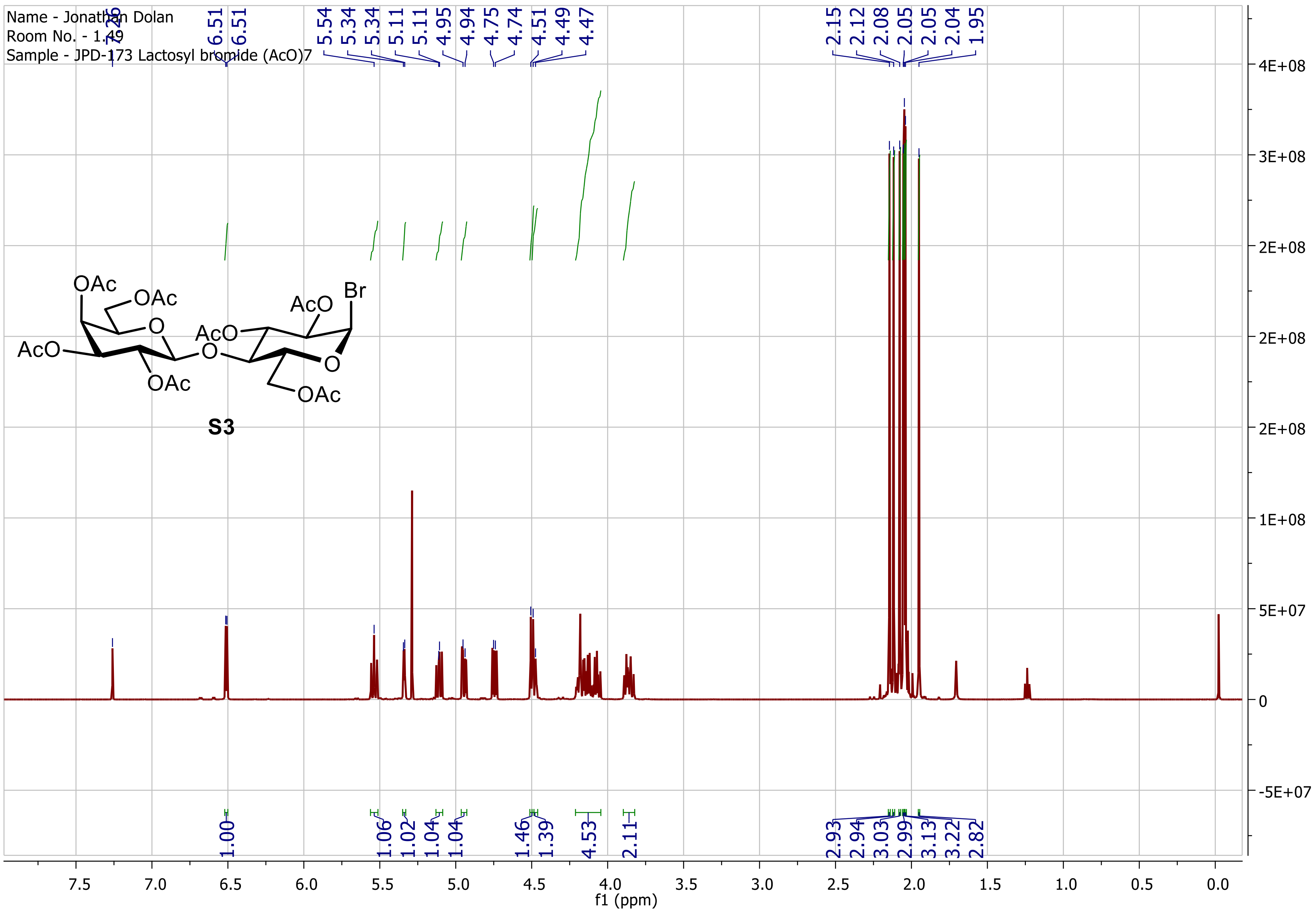
Name - Jonathan Dolan
Room No. - 1.49
Sample - JPD-173 Lactosyl bromide (AcO)7
S3
f1 (ppm)

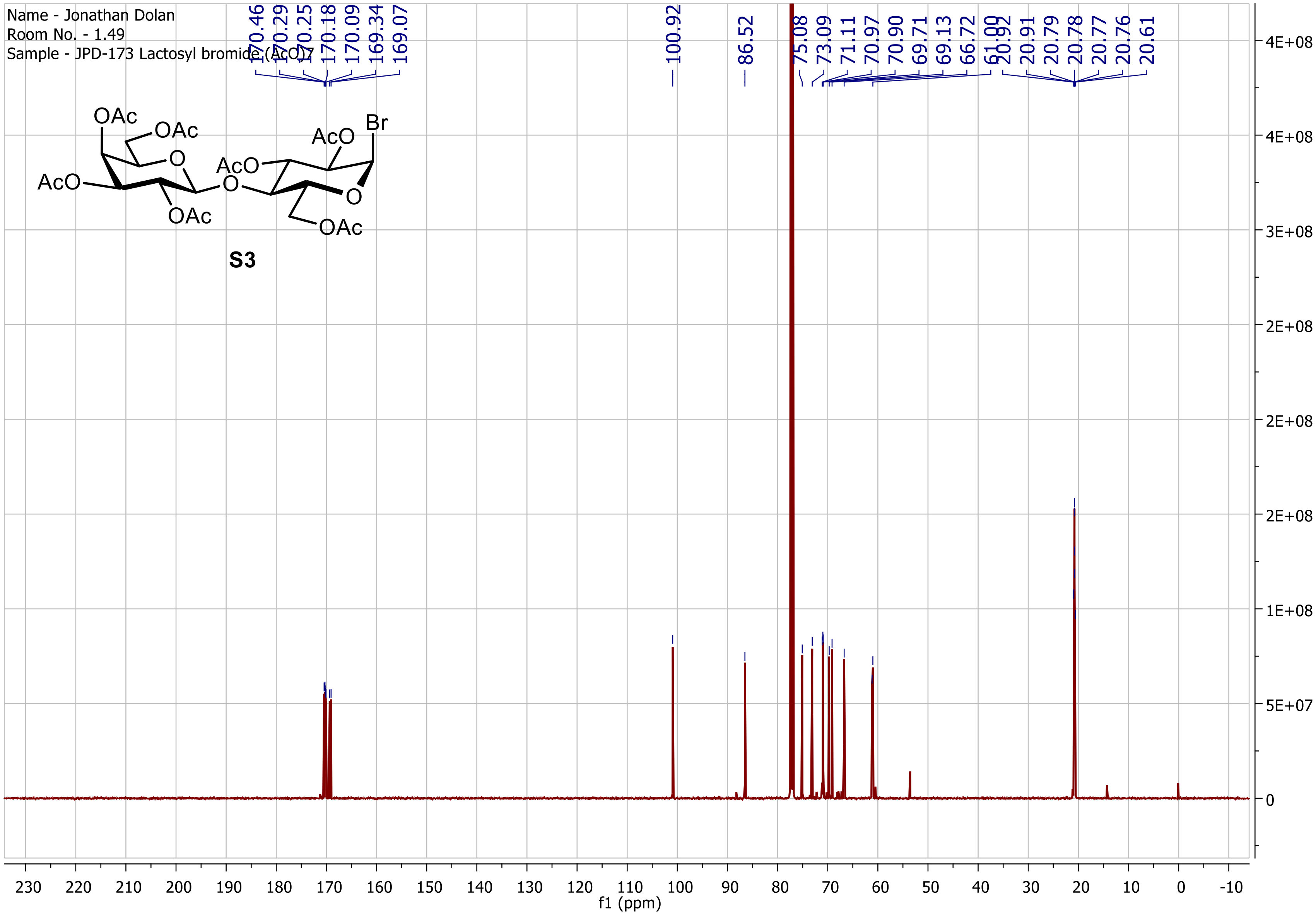

Name - Jonathan Dolan
Room No. - 1.49
Sample - JPD-173 Lactosyl bromide (AcO)7
170.46
170.29
170.25
170.18
170.09
169.34
169.07
100.92
86.52
75.08
73.09
71.11
70.97
70.90
69.71
69.13
66.72
20.91
20.79
20.78
20.77
20.76
20.61
OAc
OAc
AcO
O
AcO
O
OAc
AcO
Br
O
OAc
S3
4E+08
4E+08
3E+08
2E+08
2E+08
2E+08
1E+08
5E+07
0
230 220 210 200 190 180 170 160 150 140 130 120 110 100 90 80 70 60 50 40 30 20 10 0 -10
f1 (ppm)

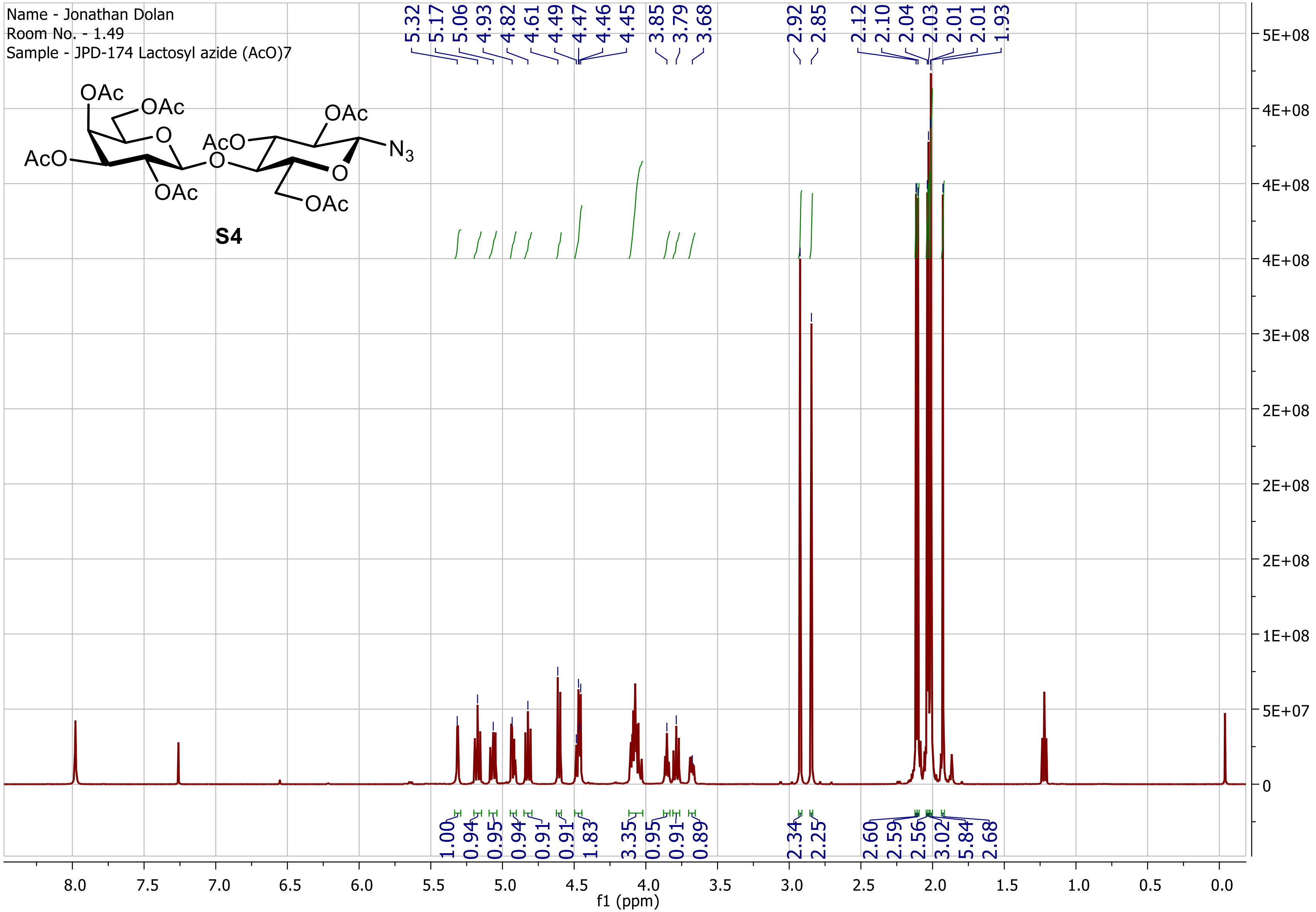

Name - Jonathan Dolan
Room No. - 1.49
Sample - JPD-174 Lactosyl azide (AcO)7
OAc
OAc
O
AcO
AcO
O
OAc
OAc
O
N_3
OAc
S4
5.32 5.17 5.06 4.93 4.82 4.61 4.49 4.47 4.46 4.45 3.85 3.79 3.68 2.92 2.85 2.12 2.10 2.04 2.03 2.01 2.01 1.93
1.00 0.94 0.95 0.94 0.91 0.91 1.83 3.35 0.95 0.91 0.89 2.34 2.25 2.60 2.59 2.56 3.02 5.84 2.68
f1 (ppm)

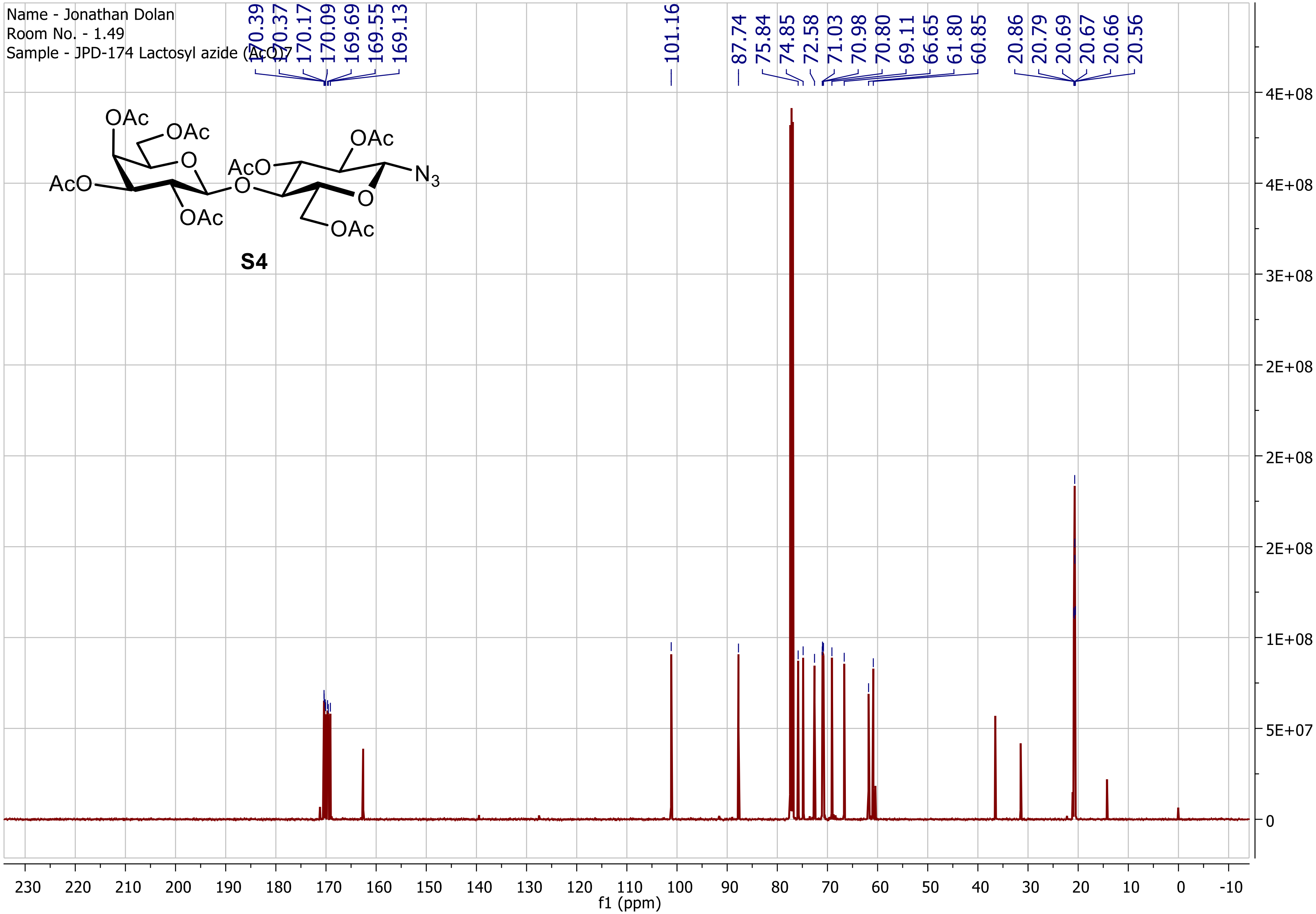

Name - Jonathan Dolan
Room No. - 1.49
Sample - JPD-174 Lactosyl azide (AcO)7
170.39
170.37
170.17
170.09
169.69
169.55
169.13
101.16
87.74
75.84
74.85
72.58
71.03
70.98
70.80
69.11
66.65
61.80
60.85
20.86
20.79
20.69
20.67
20.66
20.56
OAc
OAc
O
AcO
OAc
AcO
O
OAc
N3
O
OAc
S4
f1 (ppm)

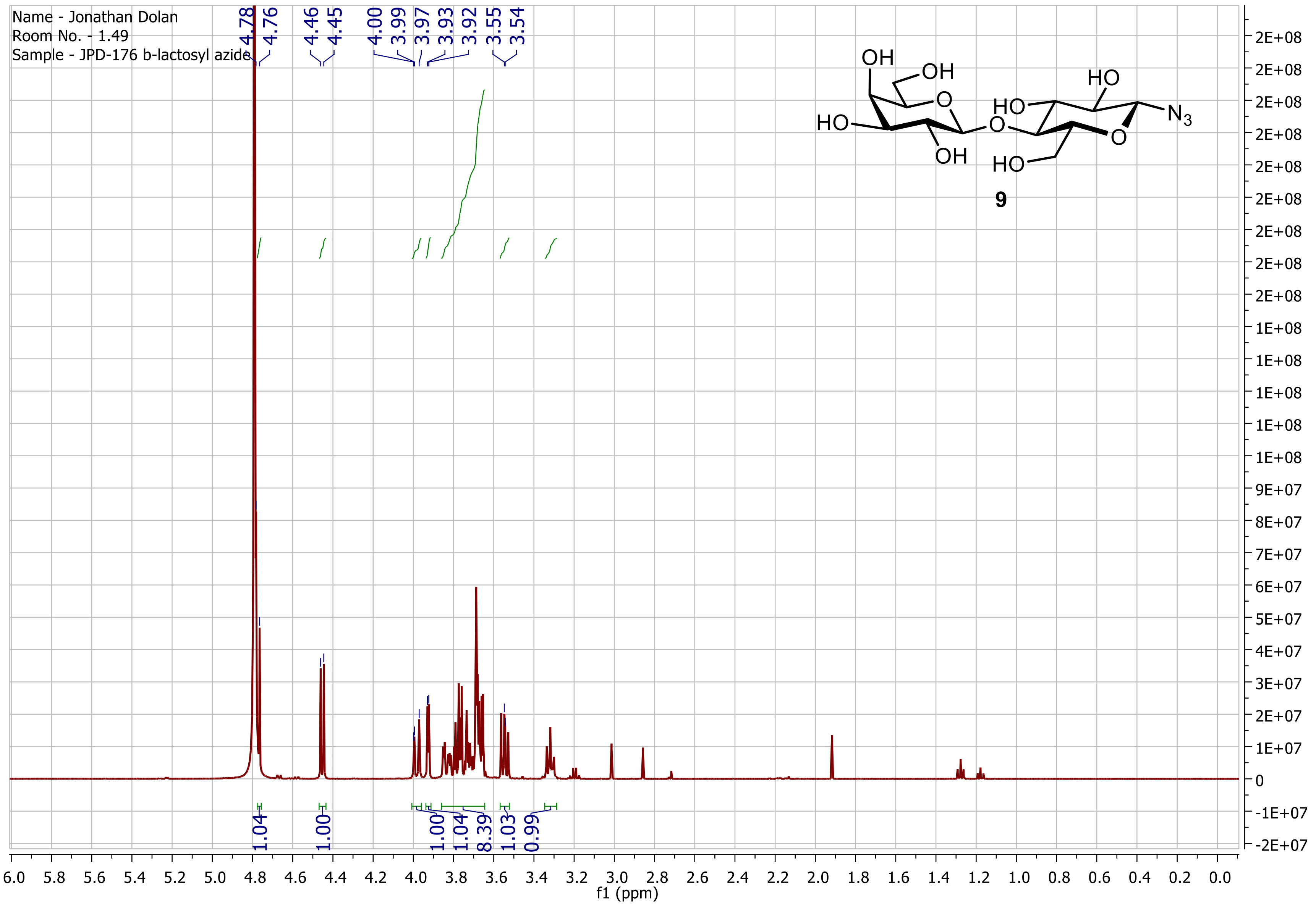
Name - Jonathan Dolan
Room No. - 1.49
Sample - JPD-176 b-lactosyl azide
4.78
4.76
4.46
4.45
4.00
3.99
3.97
3.93
3.92
3.55
3.54
1.04
1.00
1.00
1.04
8.39
1.03
0.99
f1 (ppm)
OH
OH
HO
O
HO
O
OH
HO
HO
N3
O
9

Name - Jonathan Dolan
Room No. - 1.49
Sample - JPD-176 b-lactosyl azide

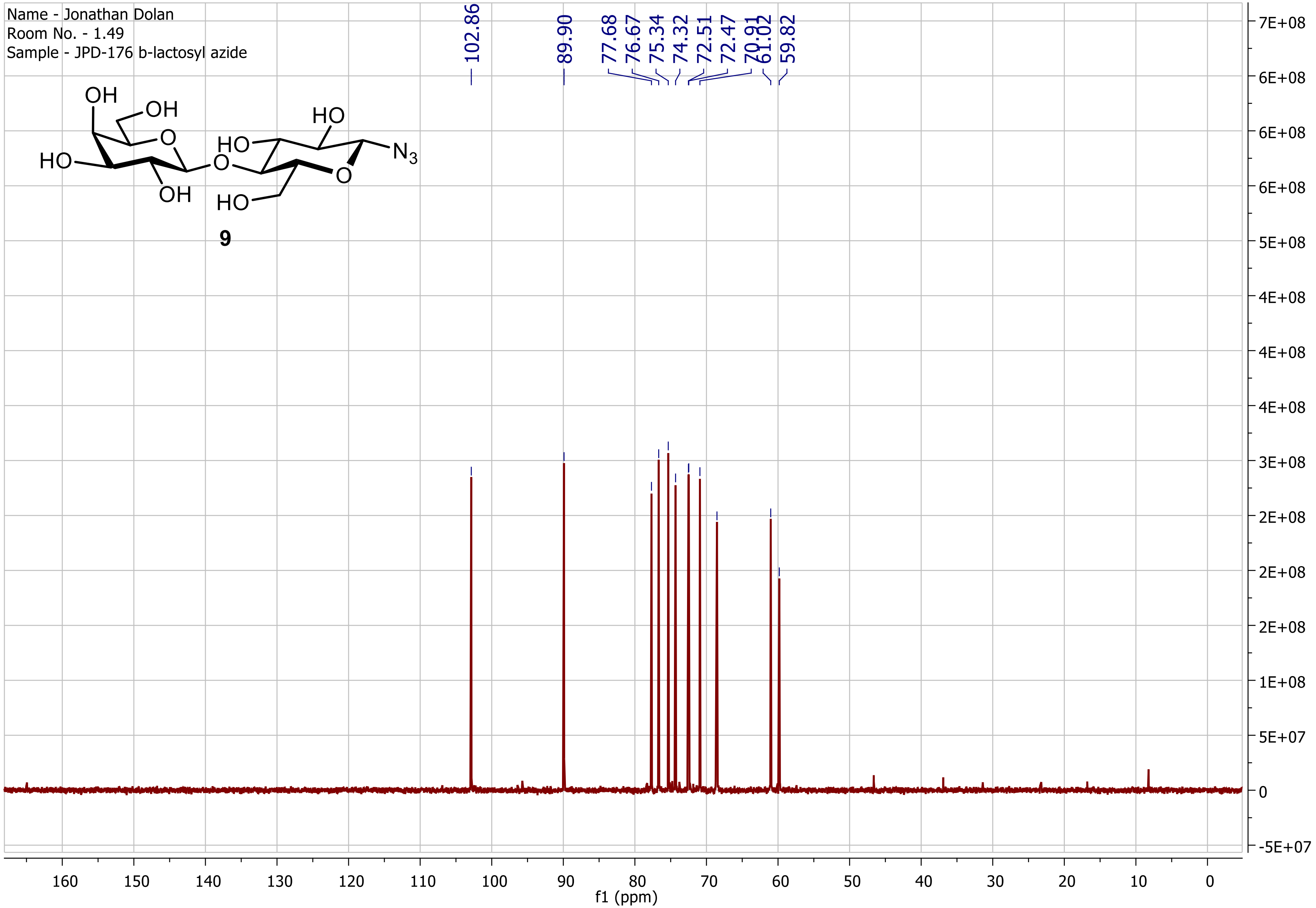

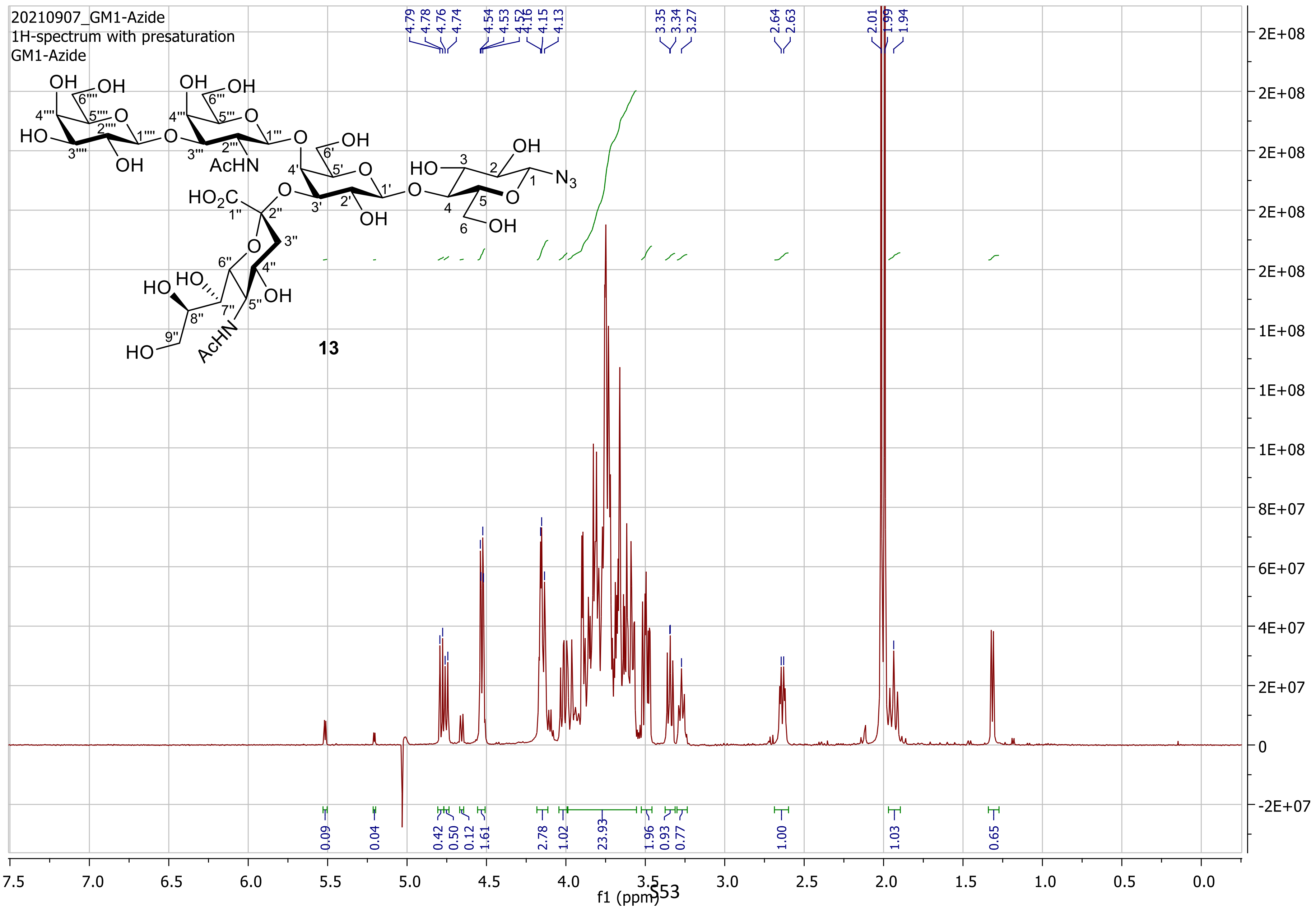
20210907_GM1-Azide
1H-spectrum with presaturation
GM1-Azide
13
f1 (ppm)

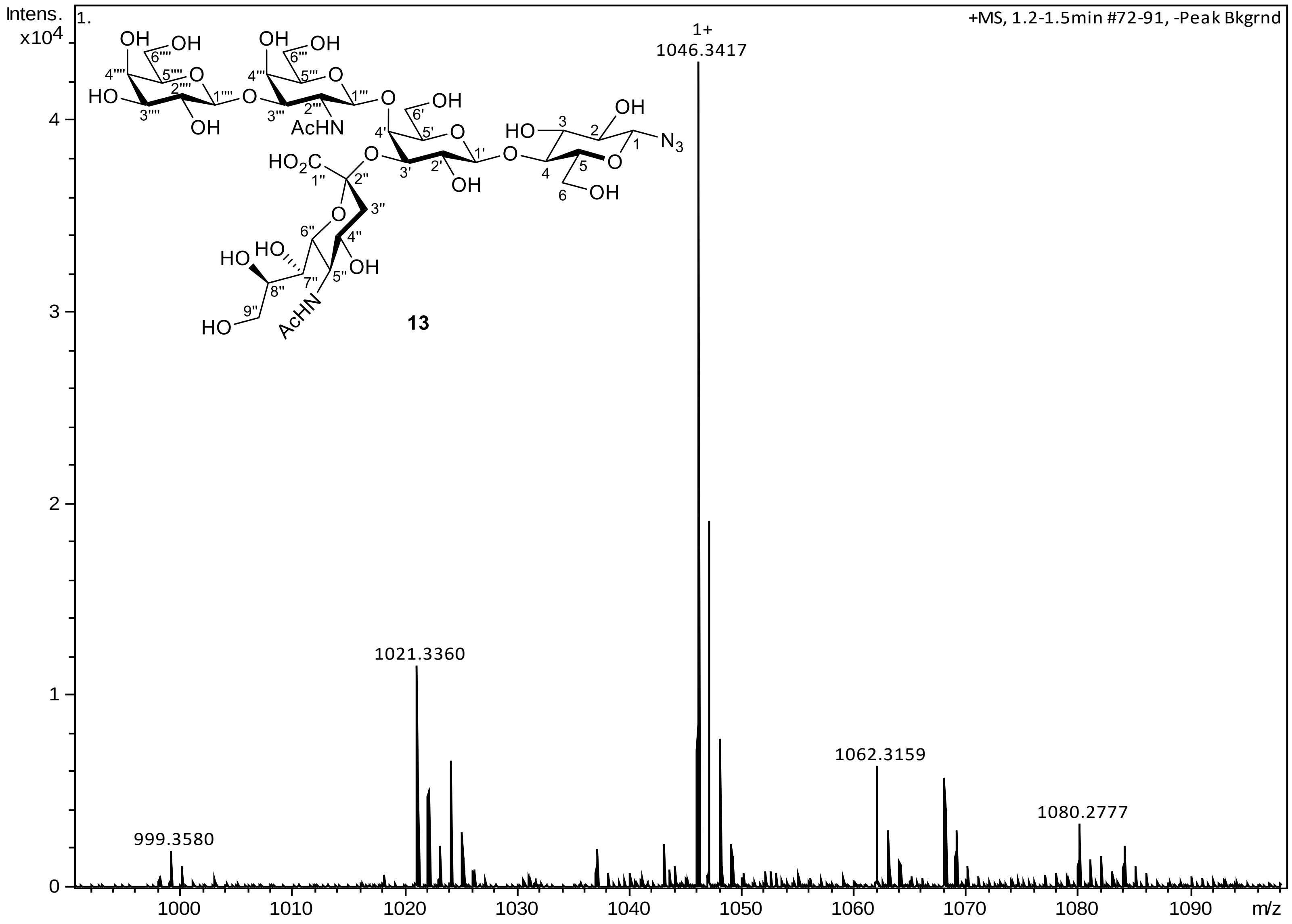

Intens.
x10^4
1.
+MS, 1.2-1.5min #72-91, -Peak Bkgrnd
1+
1046.3417
1021.3360
1062.3159
1080.2777
999.3580
13
m/z

Charge state and deconvoluted HRMS of W88E

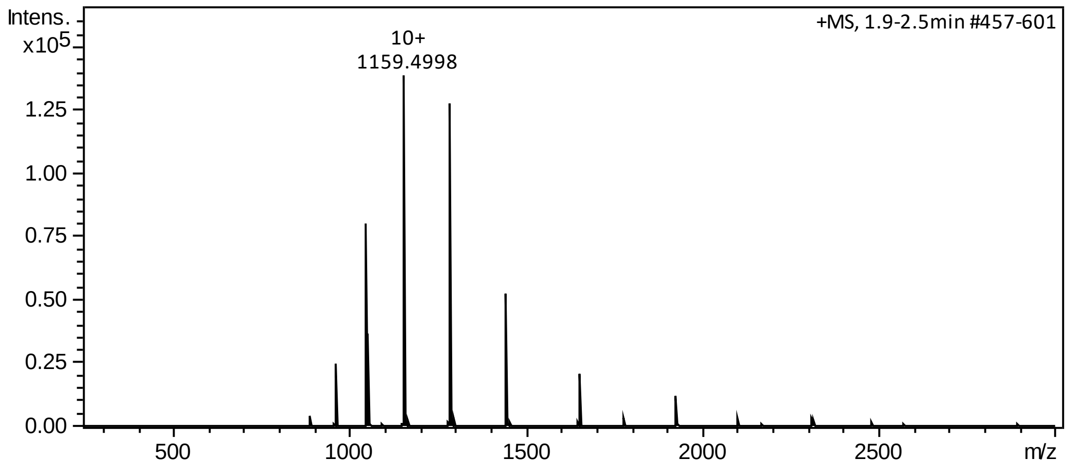

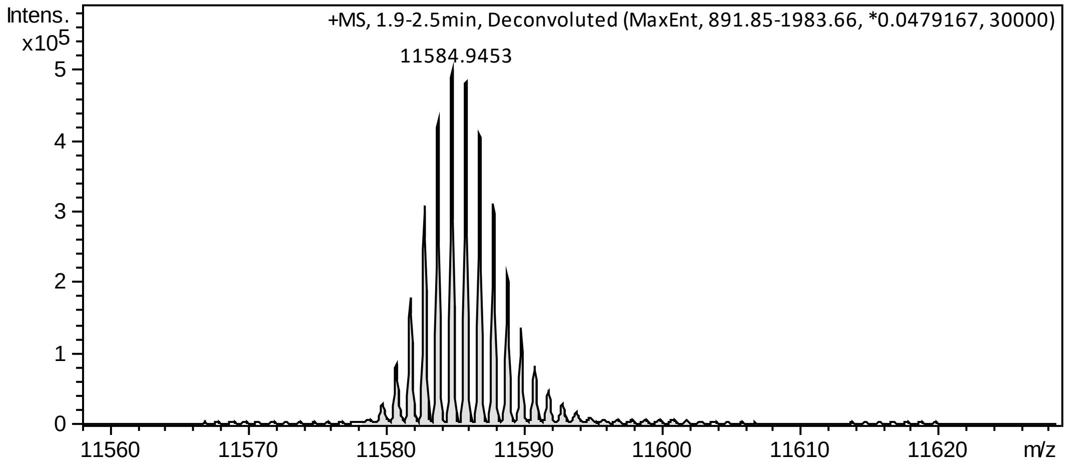

Charge state and deconvoluted HRMS of oxidised-W88E

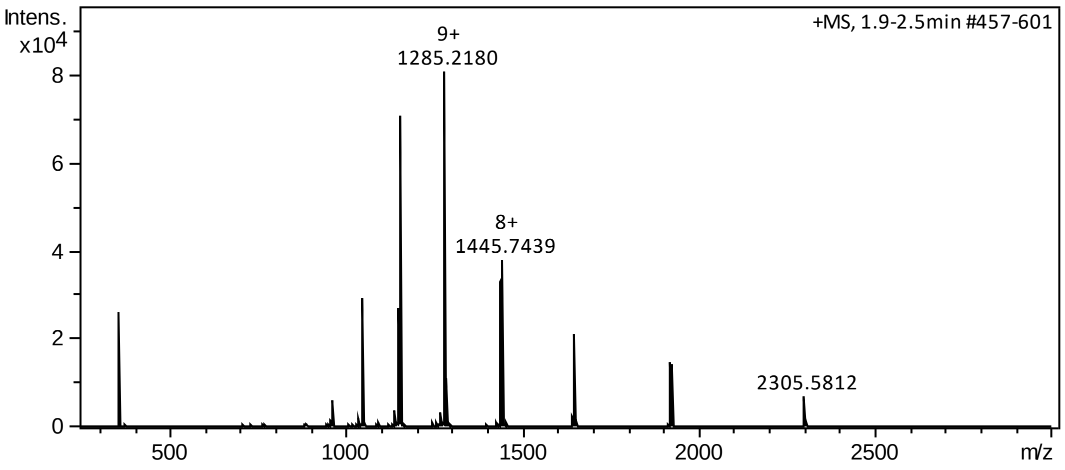

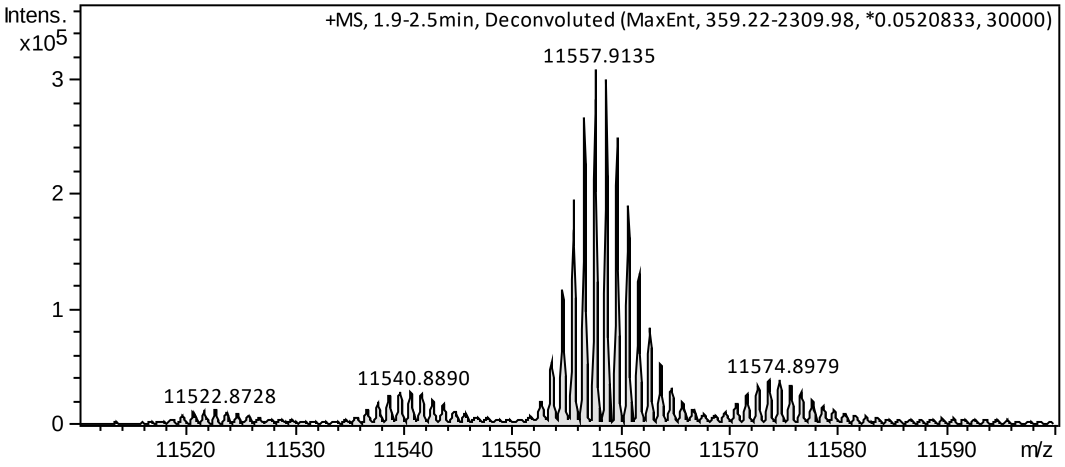

Charge state and deconvoluted HRMS of BCN-W88E

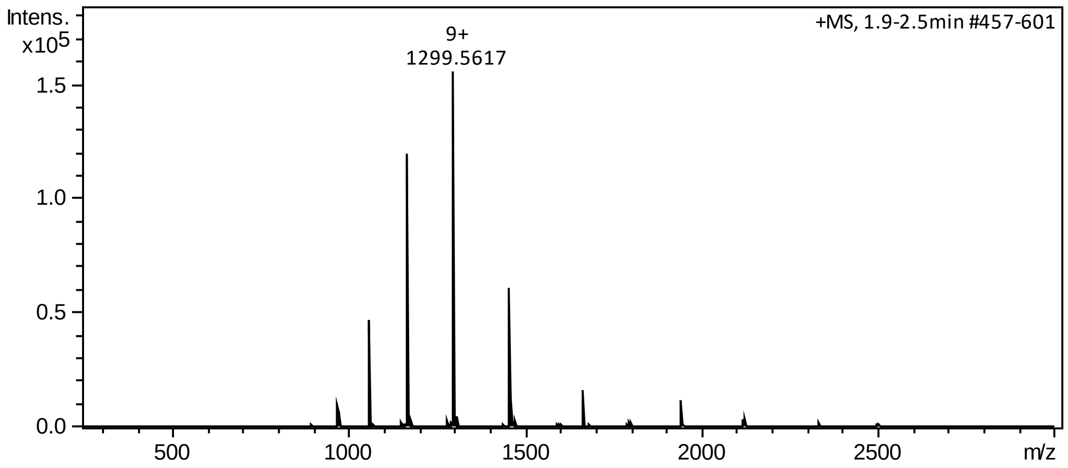

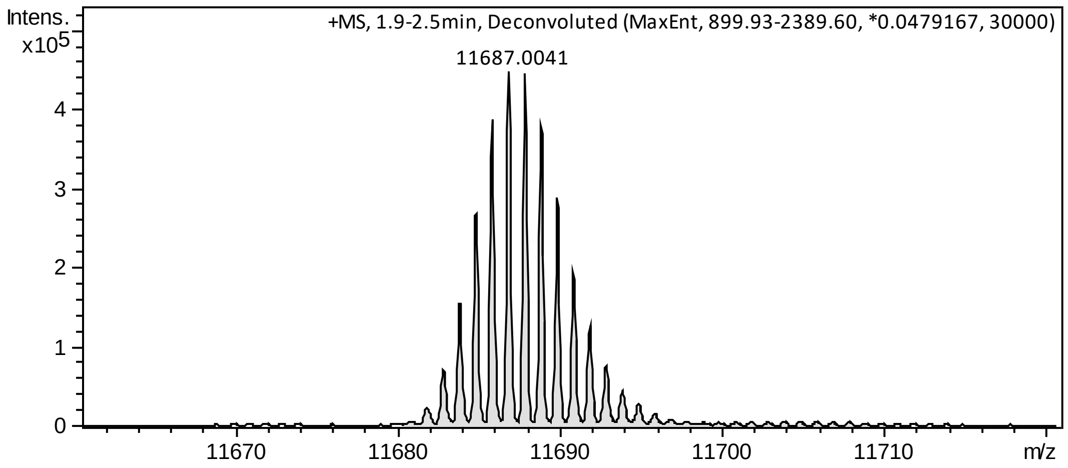

Charge state and deconvoluted HRMS of (Lac)BCN-W88E

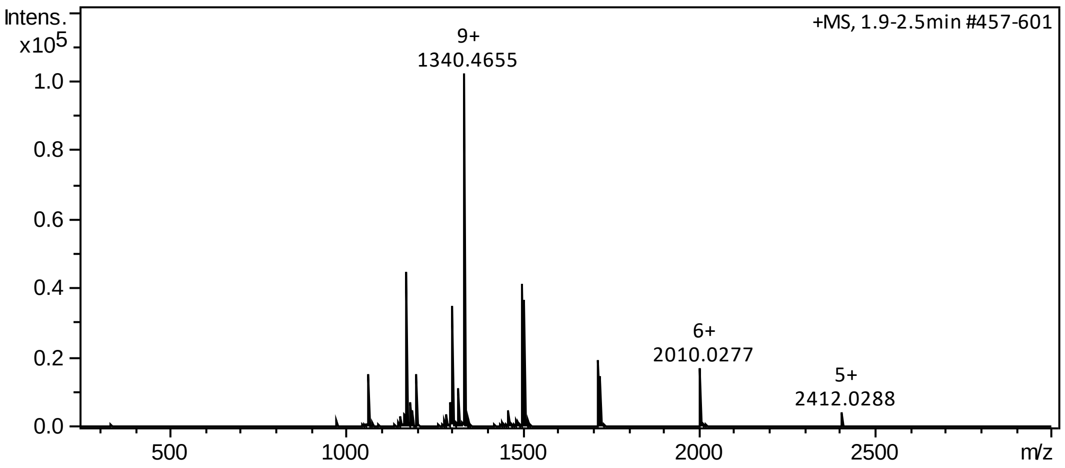

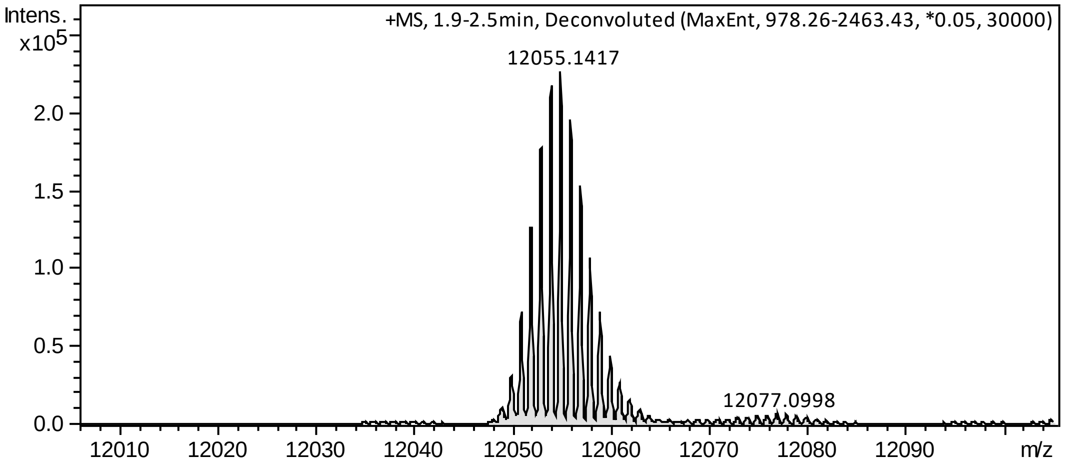

Charge state and deconvoluted HRMS of (GM1)BCN-W88E

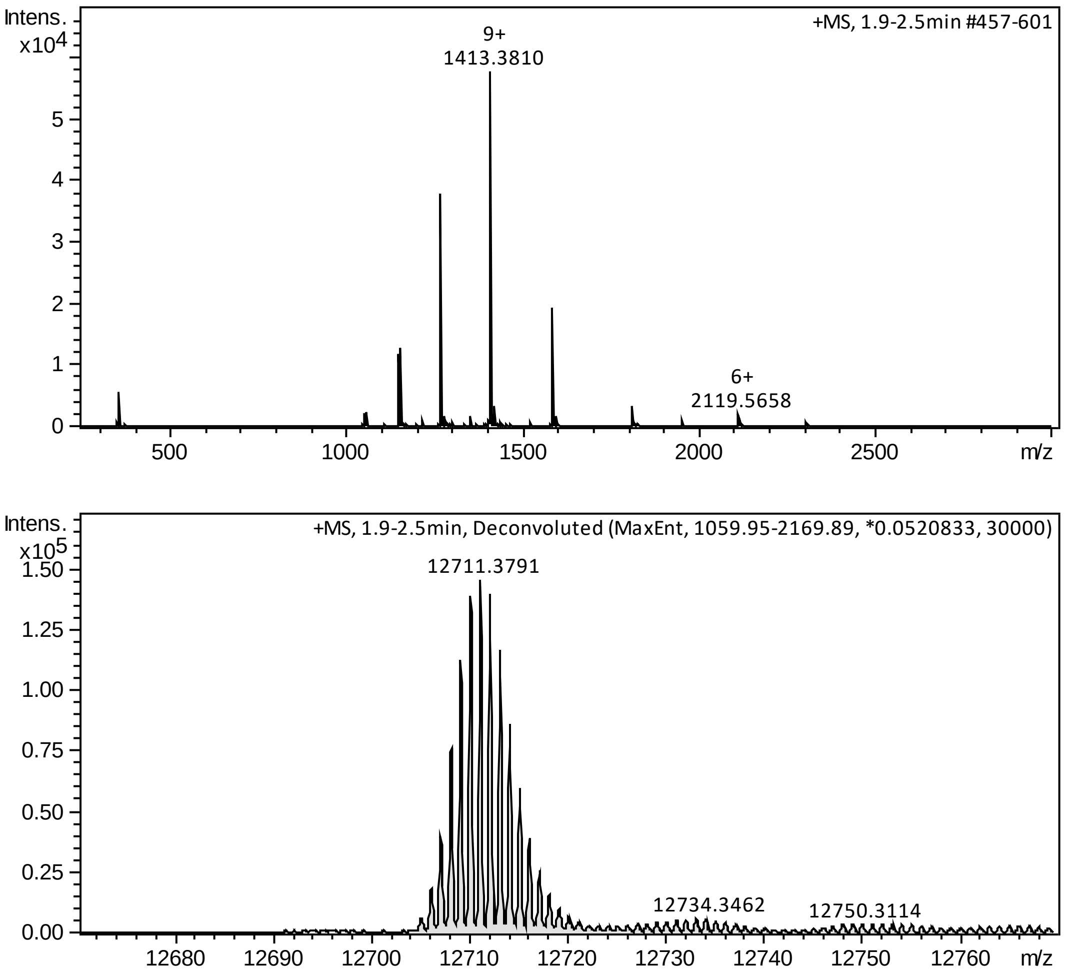

Charge state and deconvoluted HRMS of Met-W88E

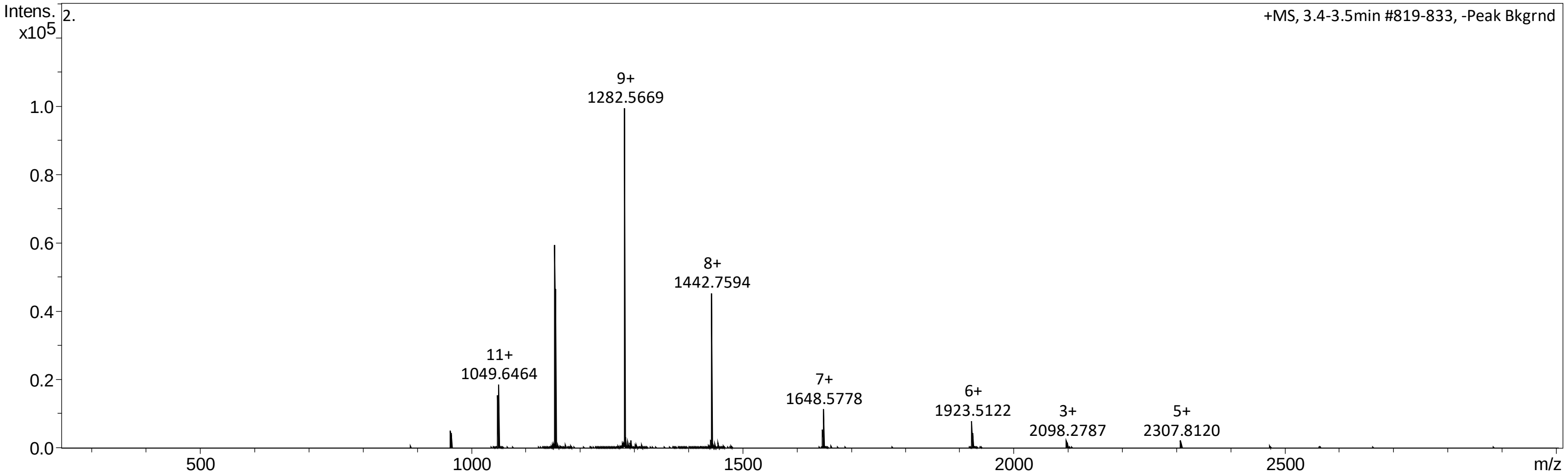

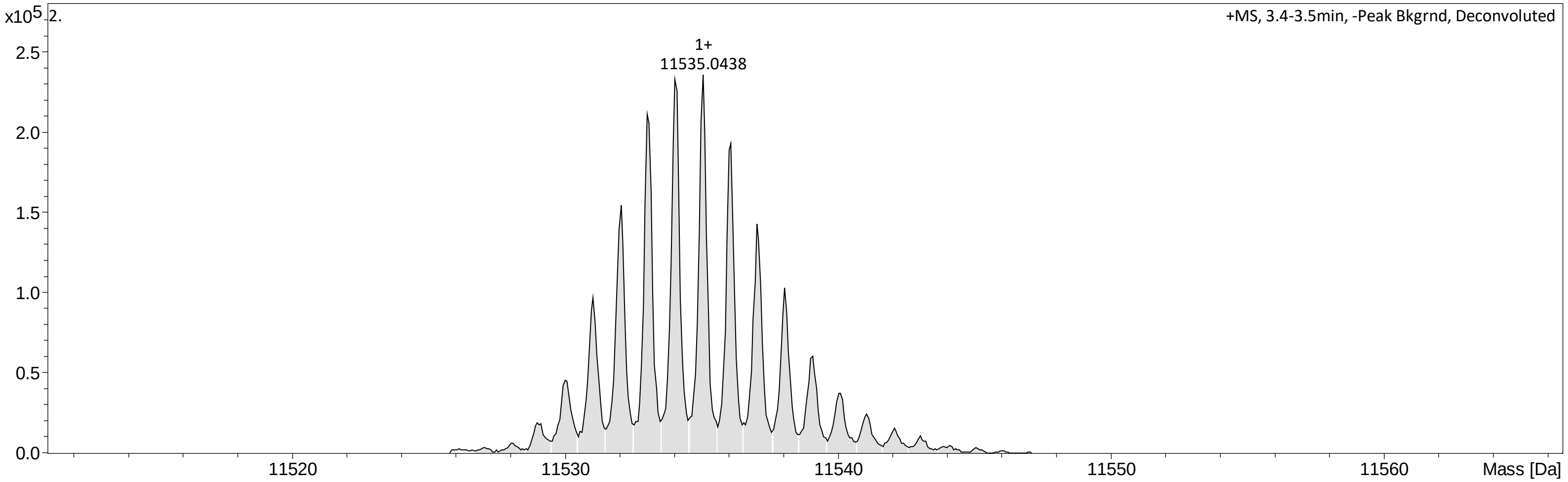

Charge state and deconvoluted HRMS of N_3-W88E

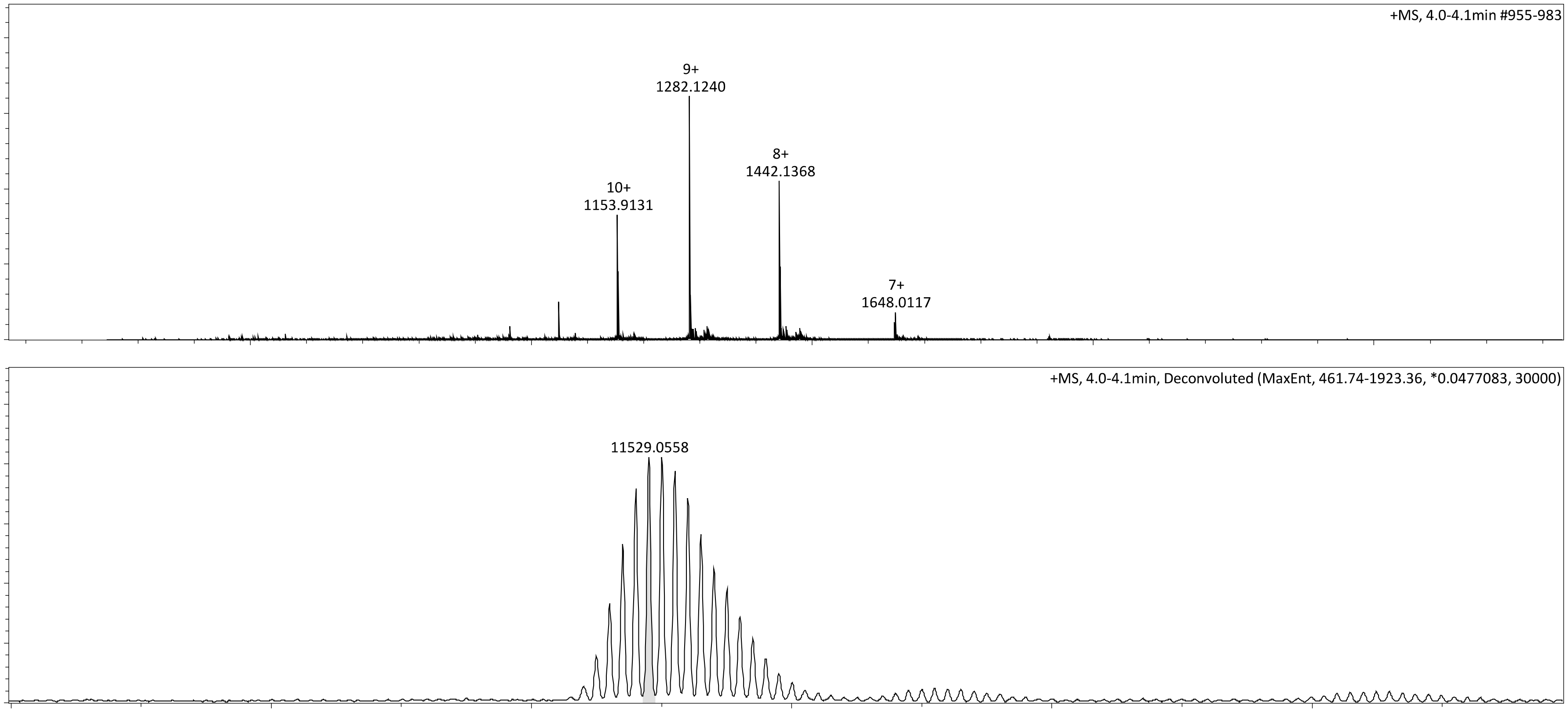

Deconvoluted HRMS time course for the SPAAC labelling of N_3-W88E with compound **6/7**

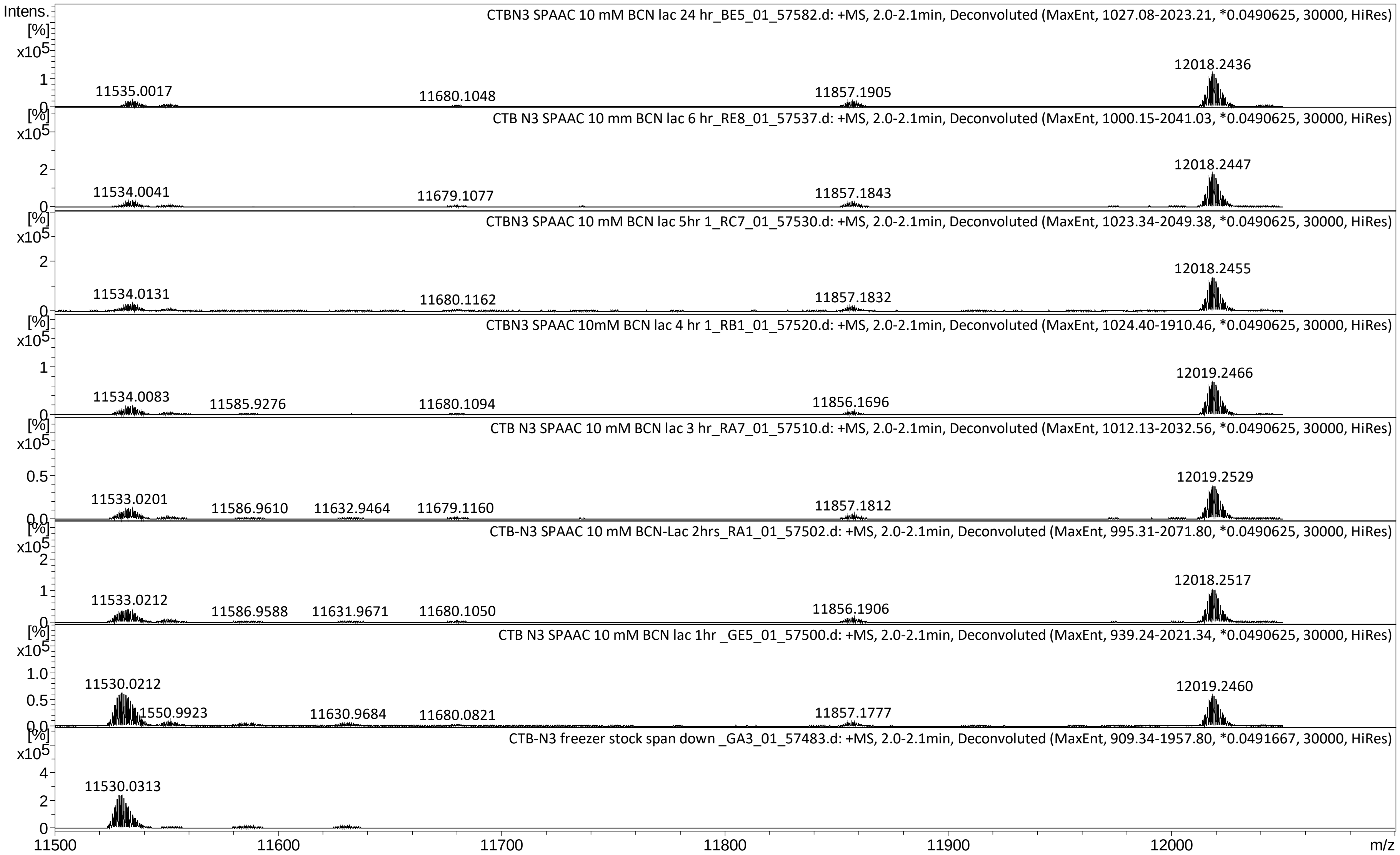

<u>Deconvoluted HRMS mass spectrum of (GM1)N_3-W88E</u>

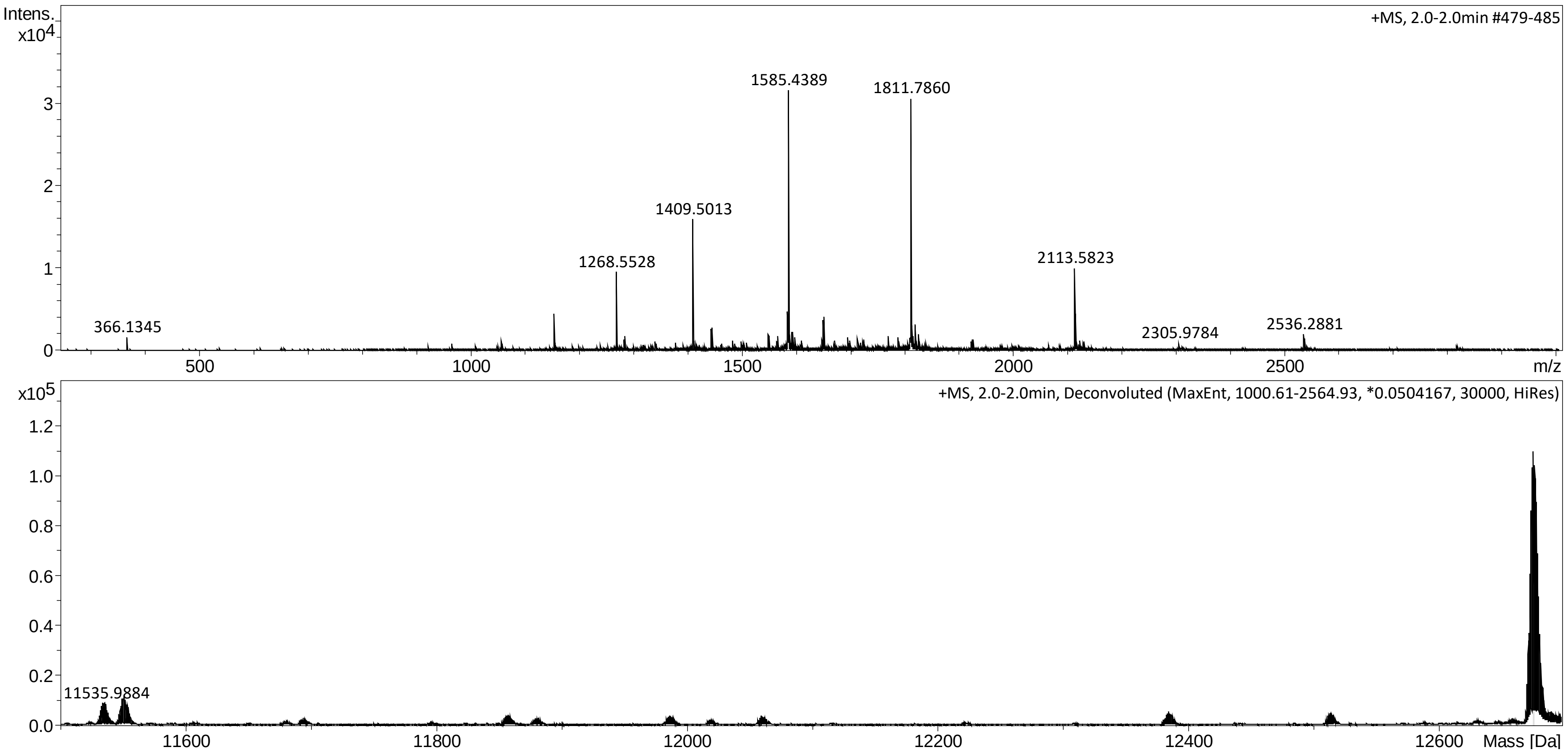

Supplement: Supplementary file 1 — au2c00312_si_001.pdf [file au2c00312_si_001.pdf]
